# A Comparative Analysis of the Temperature‐Mortality Risks Using Different Weather Datasets Across Heterogeneous Regions

**DOI:** 10.1029/2020GH000363

**Published:** 2021-05-01

**Authors:** Evan de Schrijver, Christophe L. Folly, Rochelle Schneider, Dominic Royé, Oscar H. Franco, Antonio Gasparrini, Ana M. Vicedo‐Cabrera

**Affiliations:** ^1^ Institute of Social and Preventive Medicine (ISPM) University of Bern Bern Switzerland; ^2^ Oeschger Center for Climate Change Research (OCCR) University of Bern Bern Switzerland; ^3^ Graduate school of Health Sciences (GHS) University of Bern Bern Switzerland; ^4^ Ф‐Lab European Space Agency (ESA/ESRIN) Frascati Italy; ^5^ Forecast Department European Centre for Medium‐Range Weather Forecast (ECMWF) Reading UK; ^6^ Centre on Climate Change and Planetary Health London School of Hygiene & Tropical Medicine, London (LSHTM) London UK; ^7^ Department of Public Health Environments and Society, London School of Hygiene & Tropical Medicine London UK; ^8^ Department of Geography University of Santiago de Compostela Santiago de Compostela Spain; ^9^ CIBER of Epidemiology and Public Health (CIBERESP) Spain; ^10^ Centre for Statistical Methodology London School of Hygiene & Tropical Medicine London UK

**Keywords:** Gridded climate dataset, spatiotemporal analysis, reanalysis, heat, cold, mortality, climate change

## Abstract

New gridded climate datasets (GCDs) on spatially resolved modeled weather data have recently been released to explore the impacts of climate change. GCDs have been suggested as potential alternatives to weather station data in epidemiological assessments on health impacts of temperature and climate change. These can be particularly useful for assessment in regions that have remained understudied due to limited or low quality weather station data. However to date, no study has critically evaluated the application of GCDs of variable spatial resolution in temperature‐mortality assessments across regions of different orography, climate, and size. Here we explored the performance of population‐weighted daily mean temperature data from the global ERA5 reanalysis dataset in the 10 regions in the United Kingdom and the 26 cantons in Switzerland, combined with two local high‐resolution GCDs (HadUK‐grid UKPOC‐9 and MeteoSwiss‐grid‐product, respectively) and compared these to weather station data and unweighted homologous series. We applied quasi‐Poisson time series regression with distributed lag nonlinear models to obtain the GCD‐ and region‐specific temperature‐mortality associations and calculated the corresponding cold‐ and heat‐related excess mortality. Although the five exposure datasets yielded different average area‐level temperature estimates, these deviations did not result in substantial variations in the temperature‐mortality association or impacts. Moreover, local population‐weighted GCDs showed better overall performance, suggesting that they could be excellent alternatives to help advance knowledge on climate change impacts in remote regions with large climate and population distribution variability, which has remained largely unexplored in present literature due to the lack of reliable exposure data.

## Key Points

- New products on spatially resolved weather datasets have become available but little is known on their suitability in health studies
- Here, different exposure datasets yielded similar patterns in temperature‐mortality impacts across heterogeneous areas
- Globally available modeled weather data could help advance knowledge on health impacts in areas with limited weather station data

## 1 Introduction

A large body of literature has linked exposure to nonoptimal ambient temperatures to adverse health outcomes (Achebak et al., 2019; Adeyeye et al., 2019; Bobb et al., 2014; Davis et al., 2016; Gasparrini et al., 2015; Hajat et al., 2016; Scovronick et al., 2018; Vicedo‐Cabrera et al., 2018). Thus far, most of these epidemiological studies have relied on weather data from stations as proxy of population exposure to outdoor temperature (Chen et al., 2019; Davis et al., 2016; Gasparrini et al., 2015; Habeeb et al., 2015; Wellenius et al., 2017). However, weather stations are unevenly distributed across regions or countries, with good spatial coverage around highly populated areas and regions in developed countries, unfortunately leaving out large parts of Asia and Africa where availability on daily climate data is limited (Alexander et al., 2006; Caesar et al., 2006; Donat et al., 2014). The sparse spatial coverage of observation networks throughout the world has therefore restricted the assessment of the temperature‐related health effects to urban areas and more developed countries only (Gasparrini et al., 2015; Guo et al., 2017; Sera et al., 2019; Vicedo‐Cabrera et al., 2018; Wellenius et al., 2017). Thus, rural, remote and developing regions have remained largely unexplored, although those regions are expected to be affected most by climate change (Watts et al., 2018).

Recently, new products on modeled weather data (gridded climate datasets, [GCD]) at global and local levels have been released for historical and future periods, which provide the opportunity to explore climate change impacts in different sectors across regions and countries (Rodríguez‐Vega et al., 2018). Global GCDs are usually a form of data reanalysis, which involves data assimilation of historic periods using modeled forecasts which are corrected by observations, to estimate historic temperatures across the full geographic extent. Conversely, local GCDs often incorporate mixed methods and spatial interpolation to more accurately derive temperatures estimates at a much higher resolution (Bosilovich et al., 2013; Parker, 2016; Perry et al., 2009; Rodríguez‐Vega et al., 2018).

Although GCDs have shown to be excellent tools in climate science, these can present important limitations that should be accounted for in epidemiological assessments. For example, global GCDs are prone to measurement error, particularly in areas proximal to the sea and/or with large differences in elevation, such as mountainous regions, due to the resolution and mixed pixel coverage (land‐sea mask), or in areas with a sparse monitor network (Donat et al., 2014; Rodríguez‐Vega et al., 2018; Soares et al., 2012; Zhao et al., 2020). This can be particularly important when using GCDs with coarser spatial resolution (e.g., 30 km grid), as modeled temperature could be highly influenced by factors (e.g., orography) that eventually only affect specific areas, possibly less populated. As a way to minimize the potential bias, population‐weighted estimates have been used in previous assessments on air pollution and temperature series estimated by weather stations (Balakrishnan et al., 2019; Bell & Ebisu, 2012; Ivy et al., 2008; Schaeffer et al., 2016; Shaddick et al., 2020; Spangler et al., 2019). Utilization of population‐weighted GCDs series could be particularly useful when conducting ecological analyses in large regions with large topographic heterogeneity and differences in population distribution since they could more closely reflect the temperature experienced by populations on average. It is particularly critical in ecological study designs to more accurately capture exposure to temperature variation, as it would help reduce exposure misclassification (classical measurement error) and ultimately increase the precision of the association (Armstrong, 1998; Zeger et al., 2006).

However, to date, no study has critically assessed the benefits of using population‐weighted temperature series from GCDs of variable spatial resolution in an epidemiological context. It is therefore imperative to explore whether application of different exposure datasets with different characteristics, such as spatial resolution could yield similar results across areas with different characteristics (Rodríguez‐Vega et al., 2018; Zhao et al., 2020).

In this study, we aimed to critically assess the differences in temperature‐related mortality risks and impacts derived from GCDs of variable characteristics and weather stations across two heterogeneous regions. We additionally aimed to explore the relevance of using population‐weighted area‐level temperatures, compared to unweighted average estimates from two GCDs (i.e., global and local GCDs) with different spatial resolutions. We compared the GCDs across the 10 regions in England and Wales and the 26 cantons (i.e., provinces) in Switzerland, representing areas with different geomorphological characteristics, temperature range, and population distribution.

## 2 Materials and Methods

### 2.1 Study Setting

The assessment was performed across the 26 cantons of Switzerland (i.e., provinces) and the 10 regions in England and Wales, which is the highest tier of subnational division in the United Kingdom. We considered these two sets of regions because of their heterogeneity in terms of size, geographical distribution of the population, orographic characteristics, and climate. This would allow us to evaluate patterns in temperature‐mortality associations and impacts across temperature datasets, which could potentially depend on the characteristics of the region.

Switzerland is considered a country with a particularly sparse population density and with an unequal population distribution mostly dominated by its irregular orography (Figure s1). The majority of the population resides in northern (Zürich, Basel) and western Switzerland (Geneva, Vaud), mainly in the big cities and their agglomerations, which are close to the major lakes and surrounded by vast extensions of green lands. In the south and east of Switzerland, the Alpine mountains crossing the country create stark differences in elevation, climate, and population distribution with inhabited regions mostly located in the valleys.

England and Wales have a more homogenous orography and population distribution compared to Switzerland since most regions are a mix between urban and rural areas (Figure s1, Table s1). Although, England and Wales do have some mountainous regions (Northeast and Northwest England and Wales with variation in altitude up to 1,000 m), the elevation differences are not as evident as in the Swiss Alps and most cities are located proximal to the coast and distant to these mountainous areas (Figure s1, Table s1). Conversely, Greater London is in clear contrast with surrounding regions as it is considered a metropolitan area with high population density.

### 2.2 All‐Cause Mortality Data

For Switzerland, we collected all‐cause daily mortality in each canton between 1989 and 2017 from the Federal Statistical Office. The study period was based on the availability of consistent temperature data from the same weather stations in Switzerland (see further details in section 2.3.1). We used data on daily all‐cause mortality for each region in England and Wales between 1993 and 2006, which is publicly available on: http://www.ag-myresearch.com/2015_gasparrini_lancet.html. It was originally obtained from the Office for National Statistics and has been described elsewhere (Armstrong et al., 2011) and used in previous assessments (e.g., Gasparrini et al., 2015).

### 2.3 Temperature Data

We gathered mean daily temperature data for each region in Switzerland, England, and Wales from three different types of sources or exposure datasets: (1) weather station data, often used as the gold standard in environmental epidemiology, (2) local high‐resolution GCDs, and (3) global GCD with coarser resolution. As explained below, we used country‐specific local GCDs and a unique global GCD for both sets of regions. For each GCD and region, we derived population‐weighted and unweighted mean daily temperatures through geographic information system techniques (for more details, see section 2.4). In sum, we created five temperature series per geographical unit in each country, one corresponding to weather station data and two pairs for each GCD.

#### 2.3.1 Weather Station Data

For Switzerland, we extracted data on daily mean temperatures between 1989 and 2017 from all stations of the MeteoSwiss ground‐level monitoring network from the IDAweb repository (Federal Office of Meteorology and Climatology MeteoSwiss, 2020). Although IDAweb provides data for all weather stations of the national network, most stations did not cover the full period between 1989 and 2017. In total, we selected 35 weather stations spread throughout Switzerland which covered the full study period. For the five cantons which did not have any weather station, we assigned the closest one of a neighboring canton. When more than one monitor was available per canton, we derived population‐weighted daily temperature series by defining 10‐km buffers around each station and assigned a weight based on the population density within that buffer (for further information on population data, see section 2.4). For England and Wales we gathered daily mean temperature data in each region between 1993 and 2006 and, which is freely available on: http://www.ag-myresearch.com/2015_gasparrini_lancet.html. Weather station temperature data was originally derived from the British Atmospheric Data Center (BADC) (British Atmospheric Data Centre, 2008) and used in earlier studies (Armstrong et al., 2011; Armstrong et al., 2019; Gasparrini et al., 2015). As described in Armstrong et al. (2011) region‐specific daily mean temperatures were estimated from 24‐h average measurements from, on average, 28 weather stations per region (Figure 2(a)). Then the population‐weighted average estimates were derived across all weather stations by using weights equal to the population residing closest to each station, which was based on Thiessen polygons surrounding each station (Armstrong et al., 2011). As the temperature data was derived from the BADC, the monitor data has undergone thorough quality control and validation before becoming publicly available. Weather stations with more than 25% of the days having missing data between 1993 and 2006 were excluded and the missing days were imputed as described in previous studies (Armstrong et al., 2011; Rückerl et al., 2007). Therefore, the total amount of missing data used in the time series analysis amounted to 0.00%. Since average temperature data from weather stations in both countries have been population weighted, we refer to them simply as weather stations instead.

**Figure 1:**
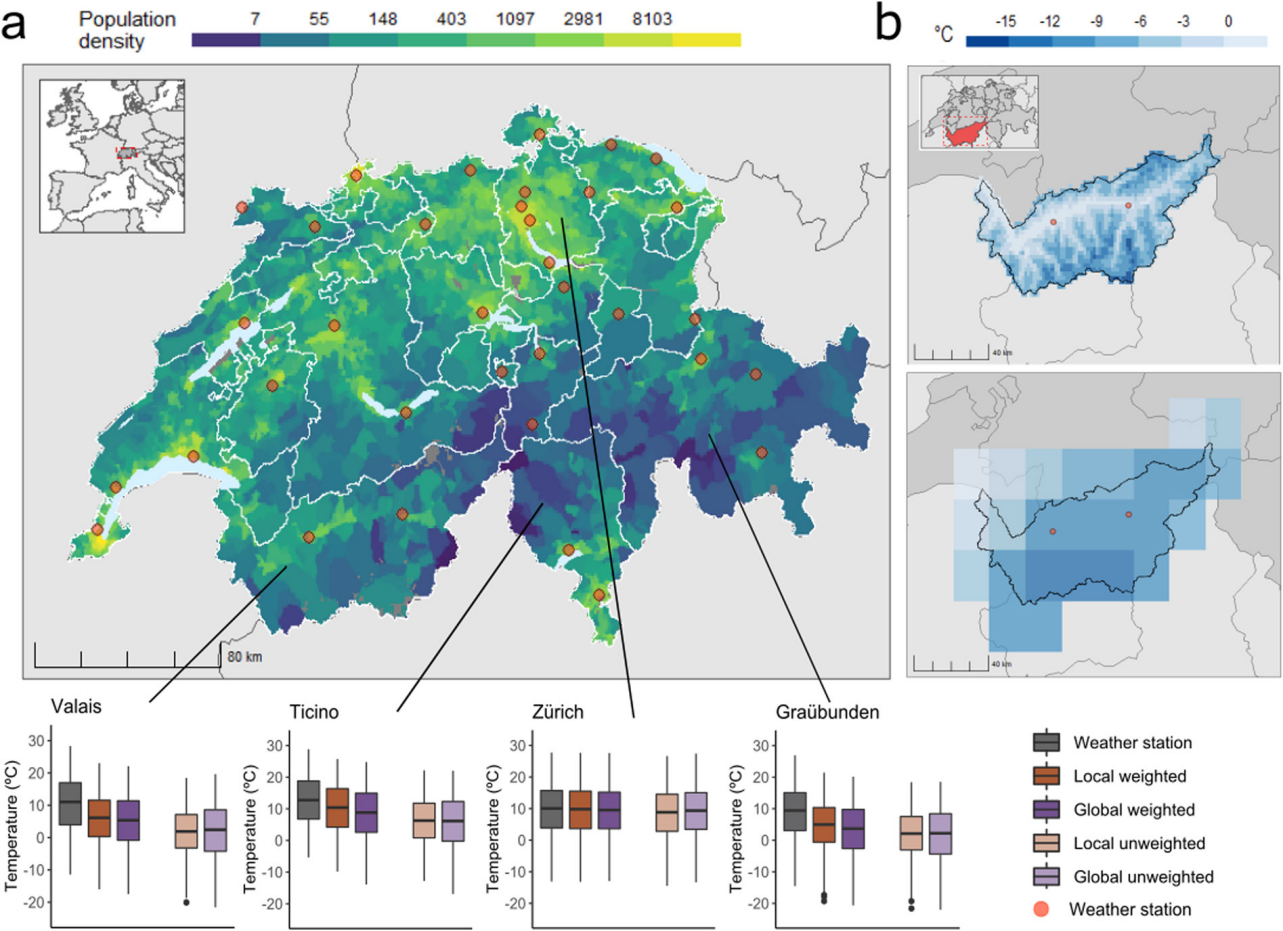
Maps showing the population density (inhabitants per squared kilometer for each corresponding grid cell (for the year 2010) and the location of the selected weather stations (*red dots*) in Switzerland. (a) Regional boxplots show the distribution of each mean daily temperature series for a set of regions in Switzerland (1989–2017). The local GCD is represented by the Meteoswiss‐product at a 1.6 × 2.3 km resolution (b, top panel) and the global GCD by the ERA5 at a 18 × 28 km resolution for Valais (b, bottom panel), a mountainous canton of Switzerland.

#### 2.3.2 GCD Data

##### 2.3.2.1 Local High‐Resolution GCD

For Switzerland, we used daily mean temperatures from the MeteoSwiss grid‐data product derived by MeteoSwiss with a 1.6 × 2.3 km grid resolution, publicly available on: https://www.meteoswiss.admin.ch/home/climate/swiss-climate-in-detail/raeumliche-klimaanalysen.html (Federal Office of Meteorology and Climatology MeteoSwiss, 2020). This temperature data is based on a combination of modeled weather forecast and observations of temperature stations covering the full Swiss geography at a high resolution.

For England and Wales, we used daily mean temperature data from the HadUK‐grid UKPC‐09 climate dataset produced by the UK MetOffice (Met Office UK, 2020). This data is based on multiple measurements of monitors throughout the country, which have been interpolated using mixed methodologies, resulting in a GCD covering the 10 regions in England and Wales at a 5‐km resolution (Perry et al., 2009). This data is publicly available on: https://www.metoffice.gov.uk/research/climate/maps-and-data/data/haduk-grid/haduk-grid.

##### 2.3.2.2 Global GCD

We used the ERA5 reanalysis GCD which provides worldwide temperature data on a spatial grid of 0.25° × 0.25° which corresponds to a horizontal resolution of approximately 28 km, for Switzerland and England and Wales. GCDs are temperature datasets based on a combination of observations (varying from ground monitors and aircrafts, to sea buoys and satellite imagery) and modeled forecasts to estimate temperatures on an hourly basis (Copernicus Climate Change Service Climate Data Storage (CDS), 2017; Herschbach et al., 2018; Rodríguez‐Vega et al., 2018). This spatially resolved temperature dataset is freely available from Copernicus Climate Data Storage provided by the European Centre for Medium‐Range Weather Forecasts and includes atmospheric variables, such as temperature, humidity as well as other variables such as snow cover (Copernicus Climate Change Service Climate Data Storage (CDS), 2017; Herschbach et al., 2018).

### 2.4 GCD Data Processing

We extracted hourly (global GCD) or daily (local GCDs) mean temperatures for each grid cell for the corresponding period covering a specific region/canton. For the former, we aggregated hourly temperature observations and created daily mean temperature averages for all grid cells throughout the regions and cantons. As mentioned before, we created two pairs of population‐weighted and unweighted temperature series for each GCD and region. For the unweighted series (i.e., without accounting for population distribution), we estimated the average values across the cell‐specific daily mean temperatures of those grid cells intersecting the boundaries of the corresponding region. Additionally, we created a single population‐weighted daily mean temperature for each region and GCD using EOSDIS gridded population data in 2010 on a 1 km horizontal grid resolution (UN WPP‐Adjusted Population Count, v4.11 – 2010) (Center for International Earth Science Information Network – CIESIN – Columbia University, 2018). Population estimates have been created using national census and population registries based on the highest national administrative boundary available (which corresponds to the municipality level in Switzerland and lower super output areas level in the England and Wales). We computed the weights in each GCD‐specific cell using the ratio between the population residing in the corresponding grid cell and the total population within that region. Finally, we computed weighted‐mean daily series for each region using mean daily temperatures of all cells in that region and the derived weights. Thus, the contribution of the cell‐specific temperature data to the total region‐specific daily mean temperature was dependent on the population residing in the grid cell relative to the total population of a region.

### 2.5 Statistical Analysis

For each temperature series and region, we performed separate quasi‐Poisson regression time series analyses with distributed lag nonlinear models to estimate the corresponding temperature‐mortality association (Gasparrini et al., 2015). The selection of models specifications was based on a previous study, which used a similar temperature dataset for England and Wales (Gasparrini et al., 2015). To account for long‐term trends and seasonality, we included a natural cubic spline of time with 8 degrees of freedom per year, together with an indicator term for day of the week. We modeled the temperature‐mortality curve with a quadratic B‐spline with three internal knots placed at the 10^th^, 75^th^, and 90^th^ percentile of region‐specific temperature distributions in the exposure dimension of the so‐called cross‐basis function of temperature (Gasparrini et al., 2010). To model the lagged‐response association, we applied a natural cubic spline with three internal knots at equally spaced values on the log‐scale up to 21 days, which captures short‐term harvesting and the long lagged associations, as was done in previous studies (Gasparrini et al., 2015). Then, we reduced the bidimensional exposure response lag‐response association into a one‐dimensional overall cumulative exposure‐response association. We plotted the region‐specific exposure‐response curves (ERC) expressed as relative risks (RR) for each temperature in the observed range, versus the minimum mortality temperature (MMT) used as the reference. This corresponds to the temperature value for which the temperature mortality risks are minimum (Gasparrini et al., 2015).

Additionally, for each GCD and region we quantified the heat‐ and cold‐related impacts in terms of excess number of deaths and mortality fractions (%) (Gasparrini & Leone, 2014). We computed the corresponding heat and cold mortality contributions by summing the daily number of temperature‐related deaths on days above the ≥75^th^ percentile or below the ≤25^th^ percentile of the temperature distribution, respectively. Differently to previous assessments, we decided to not use the MMT as threshold to define heat/cold days (Gasparrini et al., 2015), because this value changes depending on the exposure dataset. By using percentiles rather than specific MMT, we ensure that differences between impact estimates across datasets are not driven by the potentially different number of heat and cold days, but only due to deviations in mortality risk, as was done in a previous study (Achebak et al., 2019). It would ease interpretability and comparability of estimates across temperature datasets. We calculated the corresponding 95% empirical confidence interval using Monte Carlo simulations for each region and temperature series (Gasparrini & Leone, 2014). We used quasi‐Akaike Information Statistic (qAIC) to formally examine the ability of the different temperature series to predict all‐cause mortality. As an additional analysis, we quantified the impacts for very cold days (≤10^th^ percentile) and very hot days (≥90^th^ percentile), which have been attached to the supplementary file.

In a final step, we aimed to explore whether area‐level characteristics were associated with larger deviations in impact estimates obtained in each GCD, versus the weather station series. To do so, we plotted the differences between the estimated region‐specific excess mortality from the weather station series and for each GCD against regional characteristics (i.e., population density, absolute population per region, weather station density, number of GCD cells per region, and climate).

## 3 Results

### 3.1 Data Description

**Figure 2:**
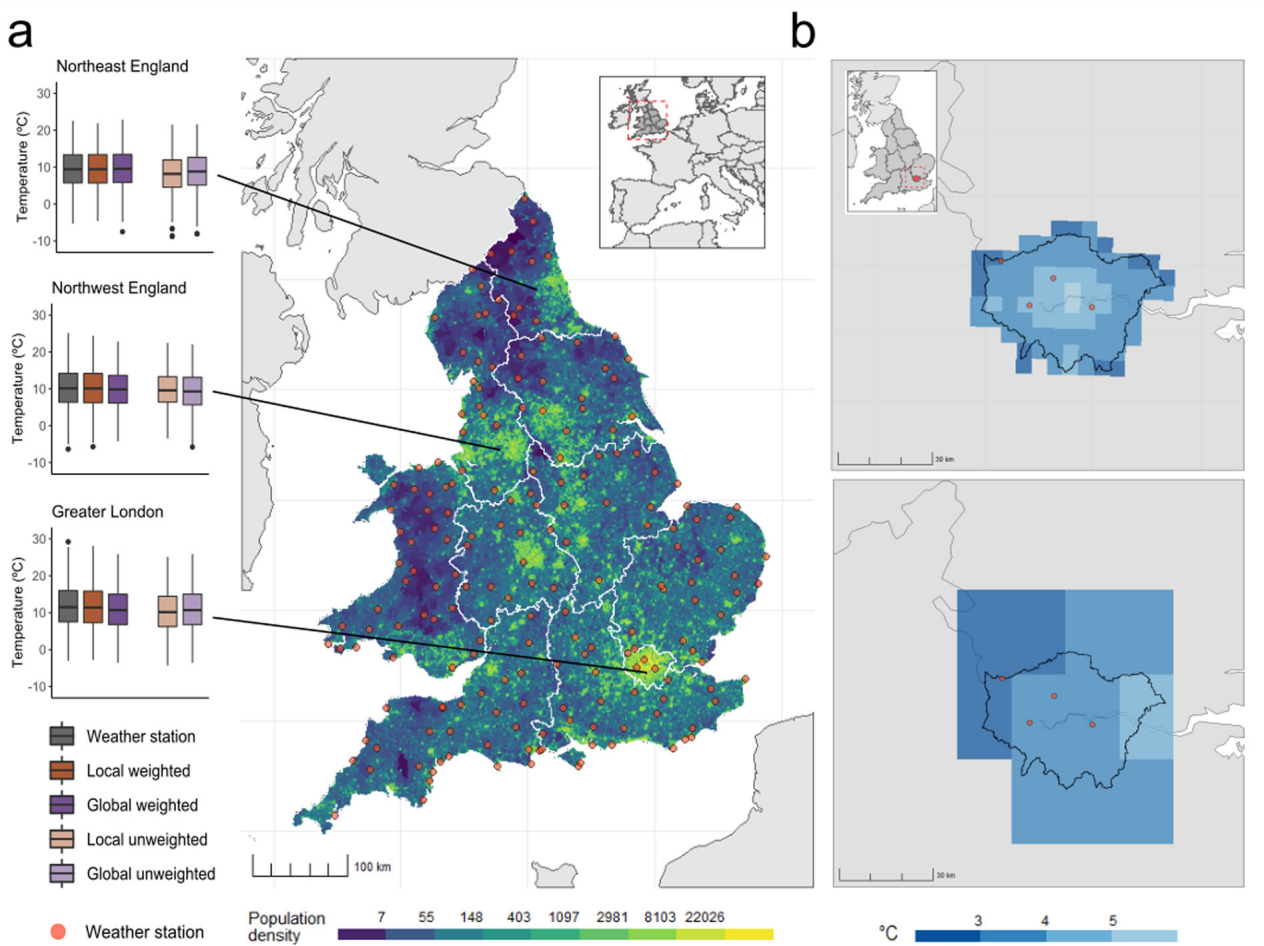
Maps showing the population density (inhabitants per squared kilometer for each corresponding grid cell (for the year 2010) and location of the selected weather stations (*red dots*) in England and Wales. (a) Regional boxplots show the distribution of each mean daily temperature series for a set of regions in England and Wales (1993–2006). For England & Wales, the local GCD is represented by the HadUK‐grid UKPC‐09 at a 5 × 5 km resolution (b, top panel) and the global GCD by the ERA5 at a 18 × 28 km resolution for Greater London (b, bottom panel).

We included 7,573,716 deaths in England and Wales between 1993 and 2006, and 1,822,622 deaths between 1989 and 2017 in Switzerland (Table s1). Figures 1(a) and 2(a) illustrate the distribution of the population, in terms of population density, and location of the selected weather stations (*red dots*), along with the temperature distribution of each exposure dataset for a set of regions for Switzerland and in England and Wales, respectively. Tables s2 and s3 and Figures s2 and s3 show the temperature distribution by dataset for Switzerland and for England and Wales. Figures 1(b) and 2(b) illustrate the spatial resolution of the local GCD and the global GCD for Valais (a mountainous canton in Switzerland) and Greater London (an urban region in England), respectively. As shown in Figure 1(a), most weather stations in Switzerland are located proximal to densely populated areas, whilst for England and Wales (Figure 2(a)) these are more equally distributed throughout rural and urban regions while having a similar monitor density as Switzerland (Table s1). For Switzerland, we observe large discrepancies across exposure datasets, in particular in the more mountainous regions (i.e., Valais, Ticino, Graubünden), where the temperature series from weather stations and population‐weighted GCDs show considerably warmer temperatures than unweighted GCD series (Ticino: Mean temperature weather station = 12.8°C, mean local unweighted GCD = 6.3°C) (Figure 1(a)) and Table s2). In urban areas and less mountainous regions, the different exposure datasets show more agreement (Figure s2 and Table s2). For England and Wales, the different exposure datasets yielded more similar distributions with only minor deviations for the weather station and two population‐weighted series which were shifted toward warmer temperature ranges, compared to the unweighted GCDs (Figure 2(a)).

### 3.2 Temperature‐Mortality Associations

**Figure 3:**
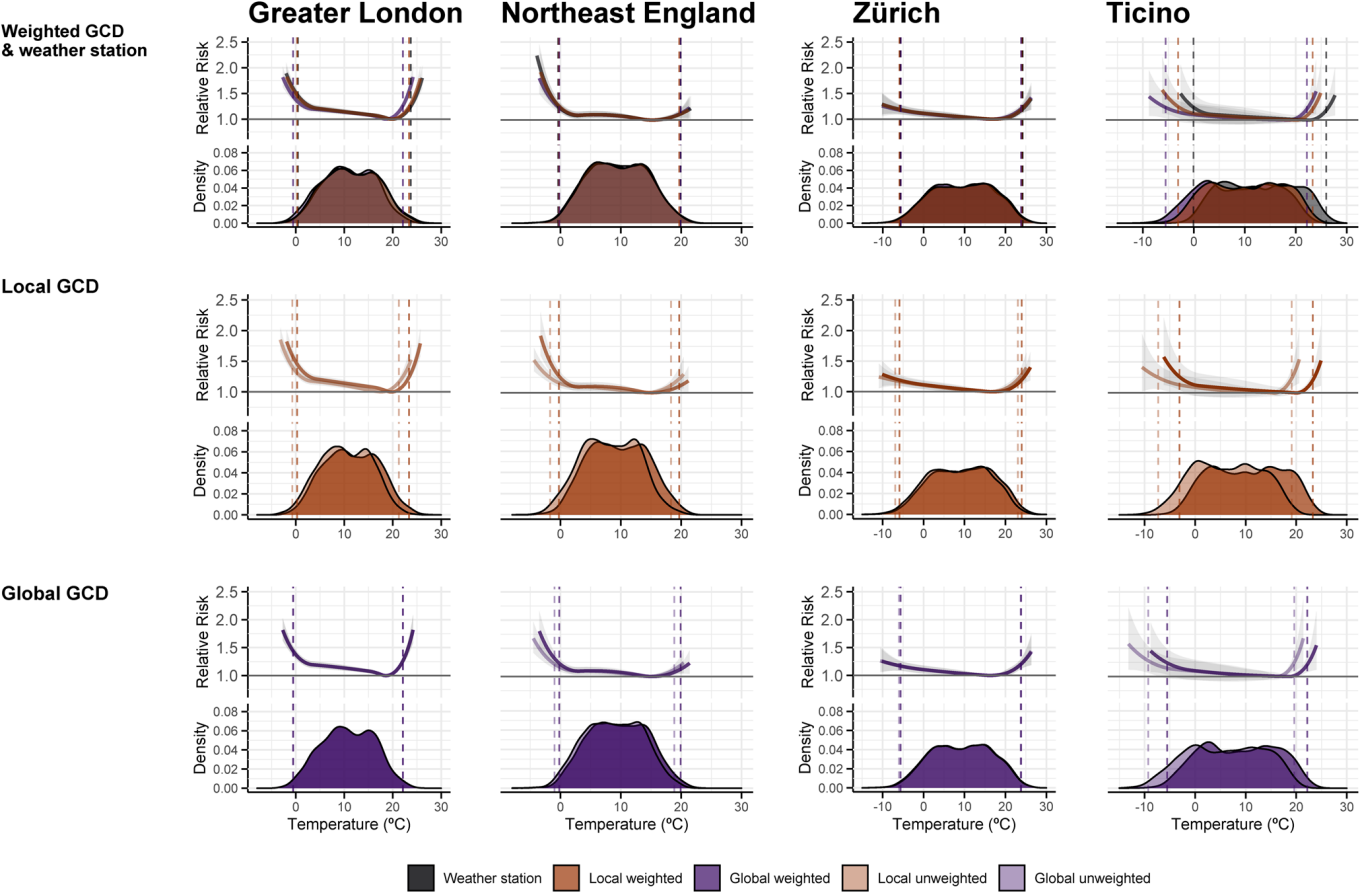
Exposure‐response curve representing the temperature‐mortality association in terms of relative risk and 95% confidence interval (shaded area) and corresponding temperature distribution (°C) for four selected regions. The *dashed line* represents the temperature at the 1^st^ and 99^th^ percentile by weather dataset. For Greater London, the local gridded climate dataset (GCD) is represented by the HadUK at a 5 km horizontal resolution. For Switzerland, the local GCD is represented by the MeteoSwiss‐grid‐product at a 1.6 × 2.3 km resolution.

Figure 3 shows the temperature‐mortality associations obtained using the five temperature series in four case‐study regions, two in England and Wales (Greater London and Northeast England) and two in Switzerland (Zürich and Ticino). To ease the comparison between exposure datasets, we selected two pairs of regions with different characteristics in terms of population distribution, orography, and climate. Greater London and Zürich are densely populated regions, while Northeast England and Ticino are good examples of large rural regions, with irregular orography and a sparse population distribution. The ERC with the corresponding RR for all regions is shown in Figures s4–s9, Tables s4–s7.

When comparing across population‐weighted series, the weather stations and local and global GCD yielded very similar ERCs. For example, the different exposure datasets provided almost identical ERCs in Zürich and Northeast England, with only slight deviations in the latter in coldest temperature ranges. Conversely, in Greater London and Ticino, we observe slight deviations in the ERCs due to differences in the absolute temperature distribution (Figure 3 top panel). However, these deviations did not translate in large differences in RR, as these are reported based on series‐specific percentiles and not on absolute temperature values. For example, RR estimates for heat in Greater London were 1.25 (95% CI; 1.19–1.30), 1.24 (95% CI; 1.18–1.29), and 1.26 (95% CI; 1.20–1.33) for the weather station and local and global GCD, respectively. When comparing the population‐weighted and unweighted local GCDs, we observe that the ERC for the former is displaced toward warmer temperatures due to the shift of the distribution (Figure 3, bottom and middle panel), although again these deviations translated into small‐to‐null differences with no consistent pattern across regions and GCDs. Interestingly, however, we found a considerably larger RR for heat in Greater London for the population‐weighted local GCD series (1.24 (95% CI; 1.18–1.29)) versus unweighted (1.16 (95% CI; 1.10–1.22)), while for the other three regions estimates were more alike (e.g., Zurich 1.19 (95% CI; 1.11–1.28) versus 1.21 (95% CI; 1.12–1.30)). For pairs of global GCDs, we found considerably smaller differences and more homogenous ERCs and corresponding RRs between the weighted and unweighted series.

According to qAIC (Figures s10 and s11, Tables s10 and s11), in England and Wales, the models using weather station series and population‐weighted local GCD series had the best predictive ability, followed by the two global GCDs, and the unweighted local series. In Switzerland, the population‐weighted series of local GCD showed higher goodness of fit compared to weather stations and the unweighted series.

### 3.3 Heat and Cold Related Excess Mortality

Table 1 shows the overall annual excess number of deaths and fractions for cold (days with mean temperature ≤25^th^ percentile) and heat (days with mean temperature ≥75^th^ percentile) for all regions in England and Wales and Switzerland. Consistent with the patterns observed across ERCs, local and global population‐weighted GCD series yielded very similar excess mortality estimates to the weather stations across all regions (Tables s10–s16). For cold, the two global GCD series and the unweighted local GCD yielded slightly lower excess mortality fractions, compared to weather stations, with larger discrepancies in England and Wales than in Switzerland. For heat, estimates from the local‐unweighted GCD for England and Wales were substantially lower compared to the other four series (e.g., excess fractions local population‐unweighted GCD = 0.38% (95% CI; 0.29–0.47) versus weather station = 0.55% (95% CI; 0.45–0.65)). While for Switzerland differences were minimal. Overall, the population‐weighted and unweighted global GCD series provided very similar mortality impacts.

**Table 1:** *Annual Excess Number of Deaths and Mortality Fractions (%) Related to Cold (≤25* ^*th*^ *Percentile) and Heat (≥75* ^*th*^ *Percentile) Estimated With Each Temperature Dataset for England and Wales and Switzerland*

		England & Wales		Switzerland	
		Cold (95% CI)	Heat (95% CI)	Cold (95% CI)	Heat (95% CI)
Weather station	Excess deaths (N)	23,036 (21,091, 25,015)	2,979 (2,419, 3,493)	1,991 (1,557, 2,365)	404 (245, 547)
	Excess fractions (%)	4.26 (3.90, 4.62)	0.55 (0.45, 0.65)	3.17 (2.48, 3.76)	0.64 (0.39, 0.87)
Local weighted	Excess deaths (N)	23,204 (21,216, 25,093)	2,943 (2,404, 3,465)	1,905 (1,509, 2,293)	409 (250, 560)
	Excess fractions (%)	4.29 (3.92, 4.64)	0.54 (0.44, 0.64)	3.03 (2.40, 3.65)	0.65 (0.40, 0.89)
Global weighted	Excess deaths (N)	20,941 (19,008, 22,822)	2,906 (2,425, 3,401)	1,853 (1,448, 2,243)	438 (270, 596)
	Excess fractions (%)	3.87 (3.51, 4.22)	0.54 (0.45, 0.63)	2.97 (2.34, 3.65)	0.70 (0.43, 0.96)
Local unweighted	Excess deaths (N)	21,033 (19,092, 22,799)	2,064 (1,550, 2,559)	1,815 (1,440, 2,176)	423 (261, 565)
	Excess fractions (%)	3.89 (3.53, 4.21)	0.38 (0.29, 0.47)	2.89 (2.29, 3.46)	0.67 (0.42, 0.90)
Global unweighted	Excess deaths (N)	20,899 (19,013, 22,770)	2,842 (2,349, 3,350)	1,871 (1,445, 2,255)	437 (268, 604)
	Excess fractions (%)	3.86 (3.51, 4.21)	0.53 (0.43, 0.62)	2.97 (2.35, 3.60)	0.69 (0.43, 0.96)

*Note*. Cold‐related mortality contributions are defined by days below the ≤25^th^ percentile of the temperature distribution, while heat‐related mortality contributions are defined by days above the ≥75^th^ percentile. N: annual number of deaths

**Figure 4:**
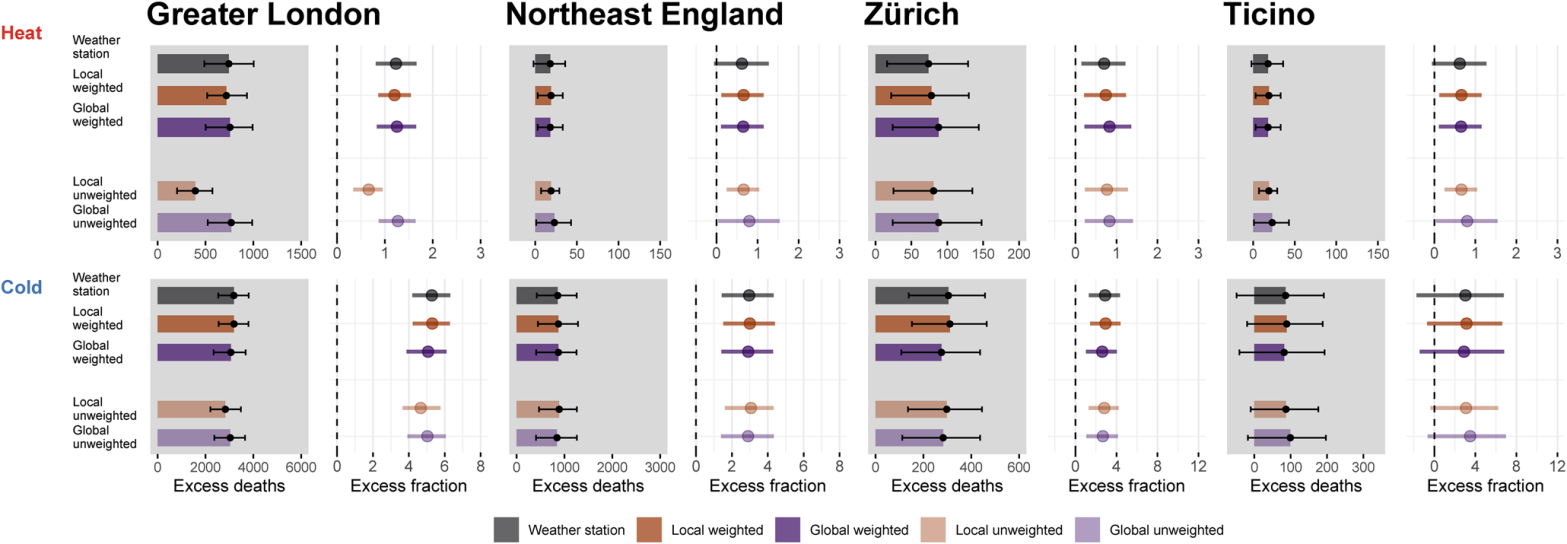
Annual excess number of deaths and mortality fractions (%) for cold‐related (≤25^th^ percentile) and heat‐related (≥75^th^ percentile) mortality estimated using the five temperature series in four selected regions. The *barplots* represent the annual excess number of deaths with associated 95% confidence interval by temperature series, together with the excess mortality fraction (%), represented by the dots, by exposure dataset for the four selected regions.

Additionally, to explore potential patterns across areas, Figure 4 shows the annual excess number of deaths and mortality fractions (%) for heat and cold for the four selected regions. While in the two Swiss regions and in Northeast England, the five temperature series provided very similar excess mortality estimates, large discrepancies can be observed only for heat in Greater London. Specifically, the population‐unweighted local GCD series reported a substantially smaller excess mortality fraction for heat (0.66% (95% CI; 0.34–0.95)) than the other GCDs and the weather station (1.23% (95% CI; 0.81–1.66)), consistent with the pattern observed in Table 1 for England and Wales. Additionally, we found similar patterns when considering only extreme cold days (days ≤10^th^ percentile of the temperature distribution) and extreme heat days (days ≥90^th^ percentile of the temperature distribution) (Tables s10–s16).

Lastly, we aimed to explore area‐level variables (e.g., absolute population per region, population density, weather station density, number of GCD cells, and mean temperature) which might be associated with difference in risk estimates (i.e., RR and excess mortality fractions) obtained from the GCD temperature series, versus the ones from weather stations. For both heat‐ and cold‐related mortality, all GCDs show random variation for the estimated measures of mortality impact (excess fractions) and association when plotting against explanatory variables (Figures s12–s15).

## 4 Discussion

Our findings suggest that temperature data from local and global GCDs can be a promising alternative to the usual weather station data for the assessment of health impacts associated with nonoptimal temperatures in ecological studies. Although the five exposure datasets yielded different average area‐level temperature estimates, these deviations did not result in substantial variations in the temperature‐mortality association or impacts, as the RR is defined on a relative scale (i.e. over the corresponding temperature distribution). More specifically, we observed that population‐weighted average levels derived from high resolution GCDs could provide a better approximation of the true exposure of the population compared to unweighted GCDs, especially in densely populated urban areas with large intracity temperature variability.

Overall, local high‐resolution population‐weighted GCDs yielded very similar risk and excess mortality estimates compared to the weather station series. Additionally, models using the former provided better predictability, suggesting that the high‐resolution population‐weighted GCDs could be a more suitable alternative to weather station data in epidemiological analyses. Furthermore, global population‐weighted GCDs showed similar risk estimates as the local GCD and weather stations. To date, weather station data have been treated as the gold standard in the assessment of temperature‐related health effects. Since these are usually placed near populated areas, they are supposed to closely follow the true average temperature exposure of the study population in ecological studies (Lee et al., 2016). The fact that population‐weighted series (weather station, local GCD, and global GCD) provided similar patterns, even in areas with a sparse population distribution, indicates that GCDs in combination with population density data can be particularly useful in studies performed in remote areas with limited and/or low‐quality weather station data.

Furthermore, our findings illustrate the relevance of accounting for the uneven population distribution across large regions when computing the regional‐level daily mean temperatures from GCDs.

Accounting for the population distribution is particularly critical in vast areas with irregular orography, which drive large variations in climate (i.e., due to differences in altitude) and highly heterogeneous distribution of the population as well as densely populated areas with large intracity temperature variability (i.e., urban heat island), which we observed for Greater London. Specifically, our results on model behavior (i.e., goodness‐of‐fit estimates) show that population‐weighted GCDs tend to more accurately predict the temperature‐mortality association and impacts compared to the unweighted temperature series. Temperature distributions of the population‐weighted series for both GCDs consistently shifted toward warmer temperatures, as opposed to the unweighted counterparts, because these are expected to better capture population exposure, usually residing in the warmer valleys. If no weighting is applied, area‐level temperature estimates in large regions will be partly influenced by measurements from cells covering vast mountainous surfaces characterized by colder climates but less populated. This shift was less evident in the global GCD due to the coarser resolution, as these were not able to sufficiently capture the large spatial variability in population density, which is particularly evident for Greater London (Donat et al., 2014; Soares et al., 2012; Spangler et al., 2019; Stone & Rodgers, 2001). Additionally, other studies found that complex terrains with large elevation change and areas proximal to the sea might also lead to an underestimation of the temperature since GCDs are more prone to measurement error in these regions (Donat et al., 2014; Lompar et al., 2019; Rodríguez‐Vega et al., 2018; Soares et al., 2012; Spangler et al., 2019; Zhao et al., 2020).

Our findings are consistent with a previous study which found that using a local population‐weighted GCD yielded largely similar RR and also concluded that no difference in mortality impacts was observed (Weinberger et al., 2019). Conversely, Royé et al. (2020) found slightly lower RR estimates for heat and cold when using the ERA5 dataset, which is the same global GCD applied to this study, compared to weather station data in a study across 52 Spanish cities. However, it should be noted that they did not compute average area‐level estimates but used the modeled temperature in the cell over the weather station to compare it with the corresponding measurement (Royé et al., 2020). Another study using different exposure approximations than GCDs found that the choice of temperature exposure definition (values from a single weather station or population‐weighted average from 25 monitors), did not yield different temperature‐mortality relationships (Schaeffer et al., 2016). Methodologies applied in temperature exposure studies are in contrast with the majority of air pollution studies, where utilization of population‐weighted exposure datasets are considered the norm (Balakrishnan et al., 2019; Brauer et al., 2016; Shaddick et al., 2020). Air pollutants have large spatial heterogeneity, therefore, using a single monitor for a region‐wide average concentration is more likely to introduce measurement error (Bell et al., 2011). Conversely, as the temperature‐mortality association is often studied at regional or city level, a more homogenous spatial distribution is expected which might explain the limited application of population weighting the exposure series. However, few studies have examined the associated impact of population weighting pollutant series. For example, a study found that weighting air pollution grid cells for population distribution led to a lower bias in the risk estimates as opposed to using population‐weighted/unweighted air pollution station series (Strickland et al., 2015).

The application of different exposure datasets resulted in minor differences in the estimated temperature‐mortality association and excess mortality impacts. The deviations in temperature distribution between exposure datasets previously described did not affect the estimation of the RRs as these are defined at relative scale (i.e., at common temperature percentiles). Notably, the shape of the ERC was similar across temperature series but shifted toward warmer temperatures when using population‐weighted counterparts or weather station. Large deviations were only visible at the very extreme ranges (below 1^st^ and above 99^th^ percentiles) which can be attributed to the instability of the boundaries of the curves due to the low statistical power (i.e., low number of days in such extreme ranges). Likewise, this deviation in temperature distributions did not substantially affect the quantification of excess mortality, as we usually use specific temperature percentiles for defining heat and cold contributions. Moreover, given that most temperature‐related deaths could be attributed to moderate ranges (Gasparrini et al., 2015), the differences at extreme temperatures do not affect the overall temperature‐related excess mortality burden. When using different definitions for heat and cold, such as very hot days (days above the 90^th^ percentile) or very cold days (days below the 10^th^ percentile), the pattern of mortality fractions did not change compared the definition used in this study (Tables s17–s20). Larger discrepancies were observed in England and Wales with slightly lower excess mortality estimates when using the local unweighted GCD (Table 1), particularly evident for heat, which seems to be mostly driven by the estimates in Greater London (Figure 3). As mentioned before, this could be attributed to the coarse GCDs resolution as well as the methodology underlying the temperature derivative of the local GCD, which did not allow the unweighted high‐resolution series to capture the spatial variability of temperature in extremely densely populated cities (Perry et al., 2009). When population weighting the local GCD for Greater London, the intraurban temperature variability (Urban Heat Island) is captured, as it does not smooth out the observations with the surrounding cells above the 99^th^ percentile of the temperature distribution, unlike the nonweighted counterpart.

Thus far, the sparse spatial coverage of observation networks throughout the world has often impeded the assessment of the temperature‐mortality association in developing countries and consequently, most temperature‐related studies have been conducted in urban settings and developed countries alone (Gasparrini et al., 2015; Guo et al., 2017; Sera et al., 2019; Vicedo‐Cabrera et al., 2018; Wellenius et al., 2017). As the ERA5 GCD is globally available, the results of this study provide the opportunity to explore the temperature‐mortality associations in more rural locations, or regions which have largely remained understudied due to a lack of exposure data, such as large parts of Asia and Latin America (Alexander et al., 2006; Caesar et al., 2006; Donat et al., 2014). This is important for public health since it is known that the temperature‐mortality association largely varies by region and country (Gasparrini et al., 2015) and more importantly, it is expected that climate change will be affecting developing regions the most (Watts et al., 2018; Xu et al., 2020).

Some limitations should be acknowledged. First, although GCDs provide a promising alternative to weather stations, particularly in areas with a sparse monitor network, we should be cautious with interpreting results from rural areas. Since reanalysis datasets are still model‐based data, therefore, in areas where we have less data coverage we would still expect more bias in the GCD estimated temperature compared to more densely monitored regions. Furthermore, we did not control for potential confounders such as air pollution concentrations, relative humidity, and influenza epidemics when estimating the temperature‐mortality impact. However, the role air pollution and humidity play as a confounding variable is still debated and studies found that controlling for these variables would only have a minimal to no effect on the overall temperature mortality estimates (Armstrong et al., 2019; Buckley et al., 2014; von Klot et al., 2012). Moreover, it is not expected that the influence of these confounders would differ across weather datasets. Furthermore, we used different study periods for Switzerland (1989–2017) and a shorter period for England and Wales (1993–2006) due to different availability of the weather station data. However, we consider that this would not affect our findings as these were based on within‐region comparisons where the same study period was used. Lastly, our analyses have only been applied to the daily mean temperatures of each dataset and not to other definitions of temperature (i.e., minimum or maximum temperature). Neither did we study the role of seasonality in the performance of GCDs, although there is some evidence that the success of the global GCD is also depended on seasonality and exposure metrics used (Cornes & Jones, 2013).

## 5 Conclusion

Although the five exposure datasets yielded different average area‐level temperature estimates, these deviations did not result in substantial variations in the temperature‐mortality association or impacts as the temperature‐mortality association and impacts are defined at a relative scale. Additionally, our findings suggest that population‐weighted high resolution GCD could be a promising alternative to weather station data and could be particularly useful in assessments in areas with large variation in climate and population distribution. More importantly, this study has shown that global GCDs could help advance knowledge on health‐related climate change impacts in remote regions which have remained largely unexplored in present literature due to the lack of reliable exposure data.

## Conflict of Interest

The authors declare no conflicts of interest relevant to this study.

## Supporting information

Supporting Information S1

## Data Availability

The data supporting the conclusions of this study can be found on the protected public repository “BORIS” of the University of Bern. The data is available after signing the data agreement and is attached in the DOI: http://doi.org/10.7892/boris.149875 (https://boris.unibe.ch/149875/).
