# Supplementary material for: A Comparative Analysis of the Temperature‐Mortality Risks Using Different Weather Datasets Across Heterogeneous Regions
Source: Geohealth. 2021 May 1;5(5):e2020GH000363. doi: 10.1029/2020GH000363 (PMC8143899; doi:10.1029/2020GH000363)
Supplement: Supplementary file 1 — Supporting Information S1 [file GH2-5-e2020GH000363-s001.pdf]

AGU PUBLICATIONS

GeoHealth

Supporting Information for

A comparative analysis of the temperature-mortality risks using different weather datasets across heterogeneous regions

Evan de Schrijver[1-3], Christophe L. Folly[1,3], Rochelle Schneider[4-6], Dominic Royé[7,8], Oscar H. Franco[1], Antonio Gasparrini[6,9,10], Ana M. Vicedo-Cabrera[1,2]

1. Institute of Social and Preventive Medicine (ISPM), University of Bern, Bern, Switzerland
2. Oeschger Center for Climate Change Research (OCCR), University of Bern, Bern, Switzerland.
3. Graduate school of Health Sciences (GHS), University of Bern, Bern, Switzerland
4. Φ-Lab, European Space Agency (ESA-ESRIN), Frascati, Italy
5. Forecast Department, European Centre for Medium-Range Weather Forecast (ECMWF), Reading, United Kingdom
6. Centre on Climate Change and Planetary Health, London School of Hygiene & Tropical Medicine, London (LSHTM), London, United Kingdom
7. Department of Geography, University of Santiago de Compostela, Santiago de Compostela, Spain
8. CIBER of Epidemiology and Public Health (CIBERESP), Spain
9. Department of Public Health, Environments and Society, London School of Hygiene & Tropical Medicine, London United Kingdom
10. Centre for Statistical Methodology, London School of Hygiene & Tropical Medicine, London United Kingdom

Corresponding author:

Ana M. Vicedo-Cabrera (anamaria.vicedo@ispm.unibe.ch)

Acknowledgments, Samples, and Data

- We would like to thank Prof. Ben Armstrong from the London School of Hygiene and Tropical Medicine for providing detailed information of weather monitor data used in this article for England and Wales.
- We would like to thank the Swiss Federal Statistical Office (BFS) for providing data on the daily mortality in Switzerland used in this study.
- This work was generated using Copernicus Climate Change Service (C3S) information [1989 -2017]. The authors would like to thank the European Centre for Medium-Range Weather Forecasts (ECMWF) that implements the C3S on behalf of the European Union.
- This project has received funding from:
  The European Union's Horizon 2020 research and innovation program under the Marie Skłodowska-Curie grant agreement No 801076, through the SSPH+ Global PhD Fellowship Programme in Public Health Sciences (GlobalP3HS) of the Swiss School of Public Health

  The *Medical Research Council-UK (Grant ID: MR/M022625/1)*

  The Natural Environment Research Council UK (Grant ID: NE/R009384/1)

  The European Union's Horizon 2020 Project Exhaustion (Grant ID: 820655)

  The Joint Research Center of the EU (JRC/SVQ/2020/MVP/1654)

- The authors declare no conflicts of interest relevant to this study
- The data supporting the conclusions of this study can be found on the protected public repository "BORIS" of the University of Bern. The data is available after signing the data agreement and is attached in the DOI: **10.7892/boris.149875 (https://boris.unibe.ch/149875/)**

Table of contents

Figures

Figure S1. Elevation map for the 26 cantons of Switzerland and the 10 regions in England and Wales with the location of the weather stations illustrated in red.

Figure S2. Boxplots for the mean daily temperature distribution by exposure dataset in Switzerland between 1989 and 2017 by region

Figure S3. Boxplots for the mean daily temperature distribution by exposure dataset in England and Wales between 1993 and 2006 by region

Figure S4. Exposure-response curve for the local and global population-weighted GCD and weather station data by region for Switzerland

Figure S5. Exposure-response curve for the local population-weighted and unweighted GCD by region for Switzerland

Figure S6. Exposure-response curve for the global population-weighted and unweighted GCD by region for Switzerland

Figure S7. Exposure-response curve for the local and global population-weighted GCD and weather station data for the 10 regions in England and Wales

Figure S8. Exposure-response curve for the local population-weighted and unweighted GCD for the 10 regions in England and Wales

Figure S9. Exposure response curve for the global population-weighted and unweighted GCD and temperature monitor for the 10 regions in England and Wales

Figure S10. Goodness of fit per exposure dataset measured by the qAIC (quasi-Akaike's information criterion) averaged over the 10 regions in England and Wales

Figure S11. Goodness of fit per exposure dataset measured by the qAIC (quasi-Akaike's information criterion) averaged over the 26 regions in Switzerland

Figure S12. Absolute differences in mortality fractions for heat and cold estimated by the GCD exposure datasets and the weather station, plotted against regional characteristics for Switzerland

Figure S13. Absolute relative risk (RR) for heat and cold estimated by the GCD exposure datasets and the weather station, plotted against regional characteristics for Switzerland

Figure S14. Absolute differences in mortality fractions for heat and cold estimated by the GCD exposure datasets and the temperature monitor, plotted against regional characteristics for England and Wales

Figure S15. Absolute differences in relative risk (RR) for heat and cold estimated by the GCD exposure datasets and the weather station, plotted against regional characteristics for England and Wales

Tables

Table S1. Descriptive statistics of the mortality data and regional-level indicators (area, population) and characteristics of the temperature datasets (weather station, local and global gridded climate datasets (GCD)) in England and Wales (1993-2006) and Switzerland (1989-2017)

Table S2. Descriptive statistics of the observed temperature by exposure dataset for the 26 regions in Switzerland, 1989 - 2017

Table S3. Descriptive statistics of the observed temperature by exposure dataset for the 10 regions in England and Wales, 1993 – 2006

Table S4. Relative risk (RR) for all-cause mortality (95%CI) for heat (99th percentile) by exposure dataset for Switzerland

Table S5. Relative risk (RR) for all-cause mortality (95%CI) for cold (1st percentile) by exposure dataset for Switzerland

Table S6. Relative risk (RR) for all-cause mortality (95%CI) for heat (99th percentile) by exposure dataset for England and Wales

Table S7. Relative risk (RR) for all-cause mortality (95%CI) for cold (1st percentile) by exposure dataset for England & Wales

Table S8. Goodness of fit for each exposure dataset measured by the qAIC (quasi-Akaike's information criterion) averaged over the 10 regions in England and Wales

Table S9. Goodness of fit for each exposure dataset measured by the qAIC (quasi-Akaike's information criterion) averaged over the 26 regions in Switzerland

Table S10. Annual excess number of deaths and mortality fractions (%) for cold (≤10th , ≤25th percentile) and heat (≥75th and ≥90th percentile) in Switzerland between 1989 and 2017 for each exposure dataset

Table S11. Annual excess number of deaths and mortality fractions (%) for cold (≤10th , ≤25th percentile) and heat (≥75th and ≥90th percentile) in England & Wales between 1993 and 2006 for each exposure dataset

Table S12. Annual number of excess deaths and mortality fractions (%) for cold, (≤10th , ≤25th percentile) and heat (≥75th and ≥90th percentile) for the four selected regions for each exposure dataset

Table S13. Summary statistics for the annual excess number of deaths, mortality fractions and associated 95% CI for heat (≥ 90th percentile and ≥75th percentile) in Switzerland

Table S14. Summary statistics for the annual excess number of deaths, mortality fractions and associated 95% CI for cold (≤10th percentile and ≤25th percentile) in Switzerland

Table S15. Summary statistics for the annual excess number of deaths, mortality fractions and associated 95% CI for heat (≥ 90th percentile and ≥75th percentile) for England and Wales

Table S16. Summary statistics for the annual excess number of deaths, mortality fractions and associated 95% CI for heat (≤10th percentile and ≤25th percentile) for England and Wales

Missing data Switzerland

Missing data amounted to 0.09% of the days. For temperature series with one or two missing days, we used a 4-day moving average to impute the values. In the canton of Fribourg, a total of 250 days were missing for the year 2006 and we assigned the value of the corresponding TabsD-grid cell for these missing days.

Missing data England & Wales

Missing data amounted to 0.00% of the days.

GCD processing and population weighted series.

We extracted hourly (global GCD) or daily (local GCDs) mean temperatures for each grid cell for the corresponding period covering a specific region/canton. For the former, we aggregated hourly temperature observations for each region by day and created daily mean temperature averages for all grid cells throughout the regions and cantons. All cells that intersect the canton or region were included for the analysis.

We created two pairs of population-weighted and unweighted temperature series for each GCD and region. For the unweighted series (i.e. without accounting for population distribution), we estimated the average values across the cell-specific daily mean temperatures of those grid cells intersecting the boundaries of the corresponding region.

Additionally, we created a single population-weighted daily mean temperature for each region and GCD using EOSDIS gridded population data in 2000 on a 1x1 km grid resolution (UN WPP-Adjusted Population Count, v4.11 -2000) (Centre for International Earth Science Information Network - CIESIN - Columbia University. 2018). Population estimates have been created using national census and population registries based on the highest national administrative boundary available (which corresponds to the municipality level in Switzerland and Lower Super Output Areas level in the England and Wales). We summed the total population living in the region and additionally we summed the total population that residing within each grid cell using Geographic Information System methods. Then we computed the weights in each GCD-specific cell using the ratio between the population residing in the corresponding grid cell and the total population within that region. Therefore, the contribution of the grid cell towards the full time series for a region is dependent on the population residing within the grid cell relative to the overall- region specific population. Finally, we computed weighted-mean daily series for each region using mean daily temperatures of all cells in that region and the derived weights. Thus, the contribution of the cell-specific temperature data to the total region-specific daily mean temperature was dependent on population weight of the grid cell relative to the total population of a region.

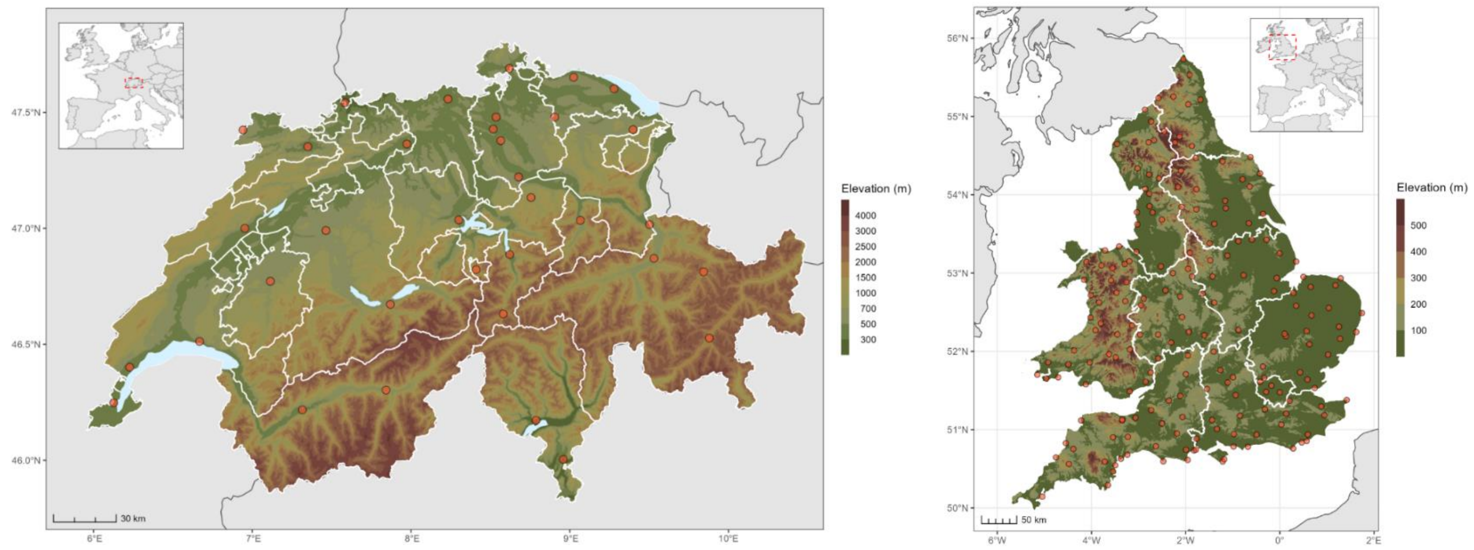

Figure S1. Elevation map for the 26 cantons of Switzerland and the 10 regions in England and Wales with the location of the weather stations illustrated in red.

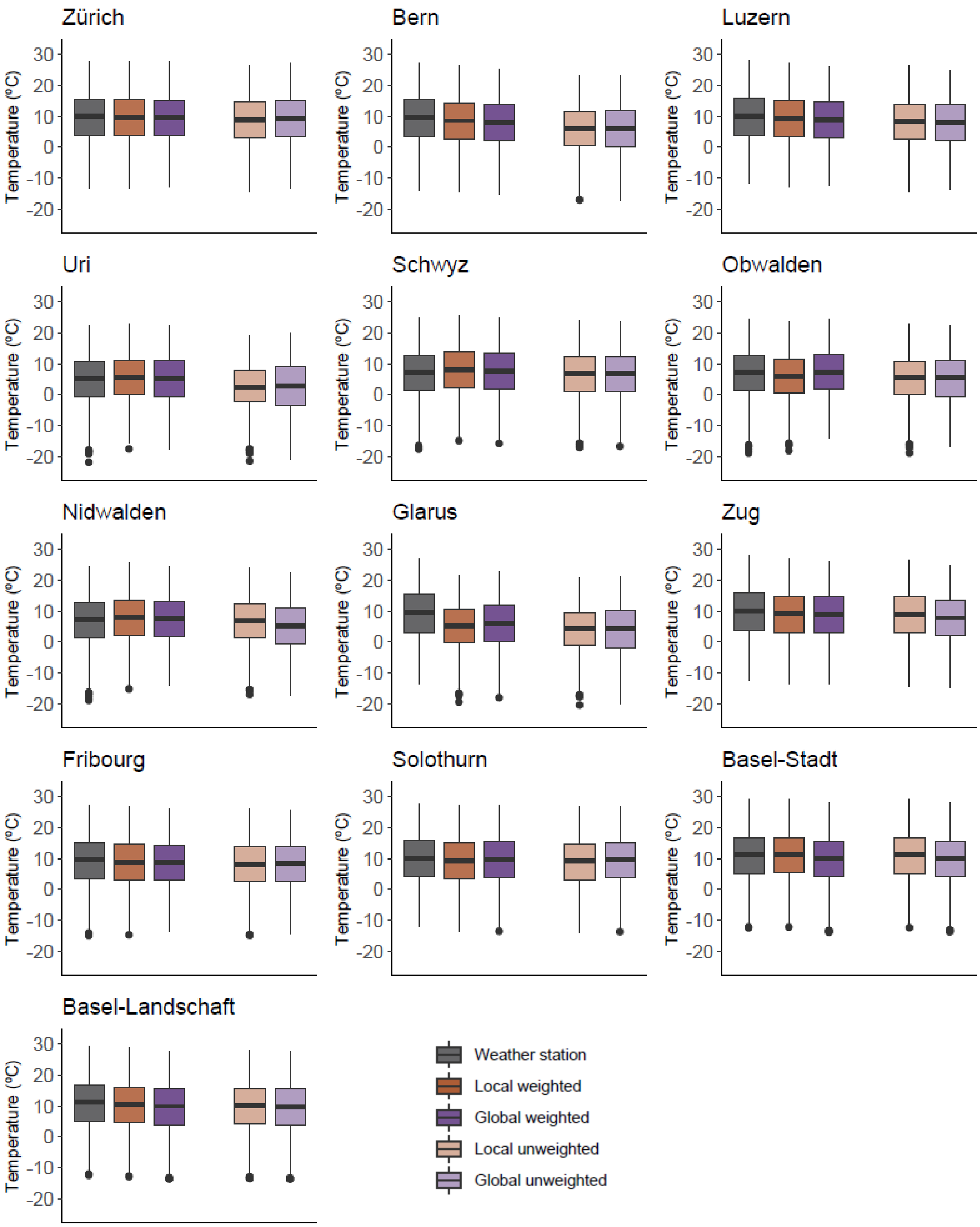

Figure S2. Boxplots for the mean daily temperature distribution by exposure dataset in Switzerland between 1989 and 2017 by region

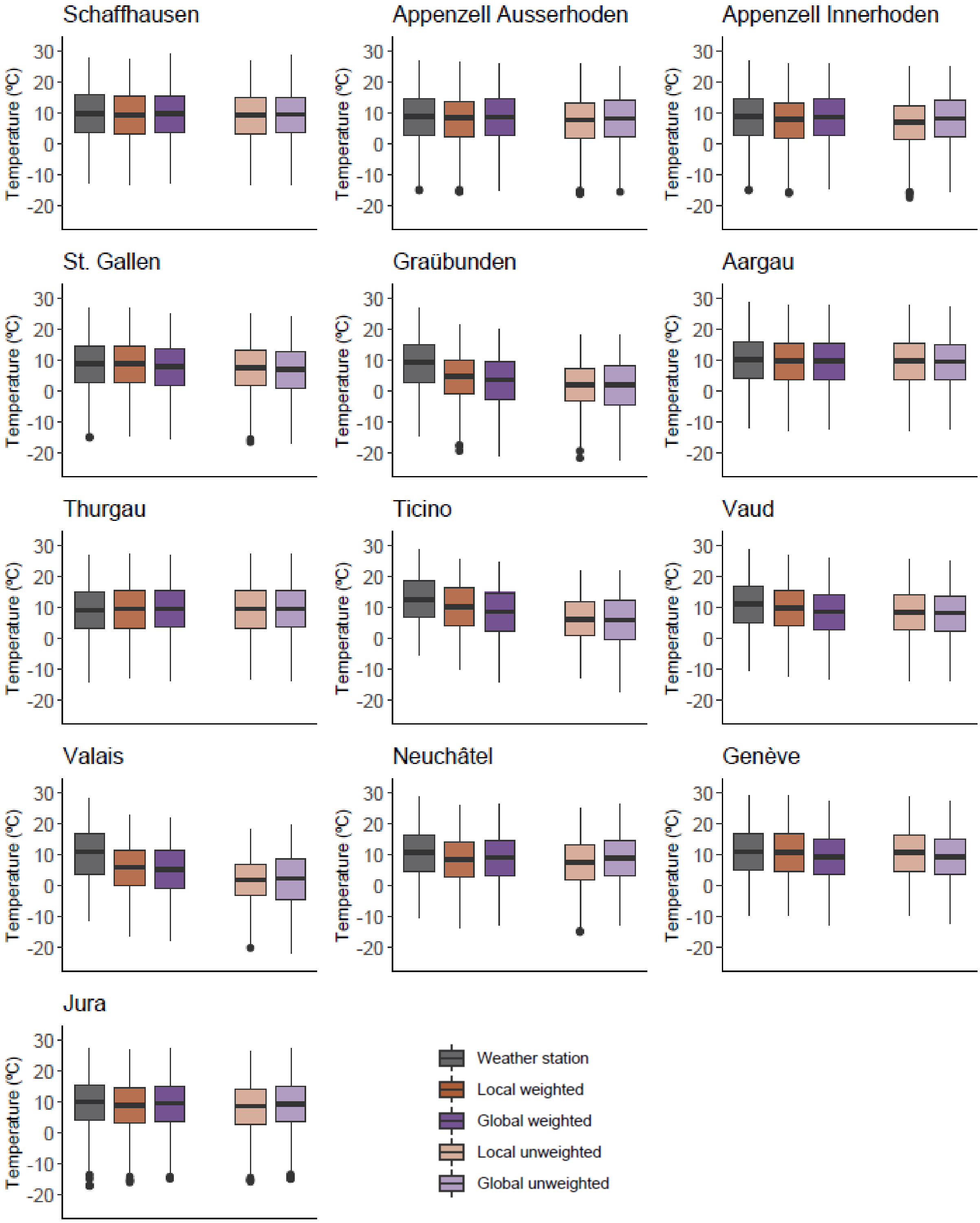

Figure S2. Boxplots for the mean daily temperature distribution by temperature exposure in Switzerland between 1989 and 2017 by region

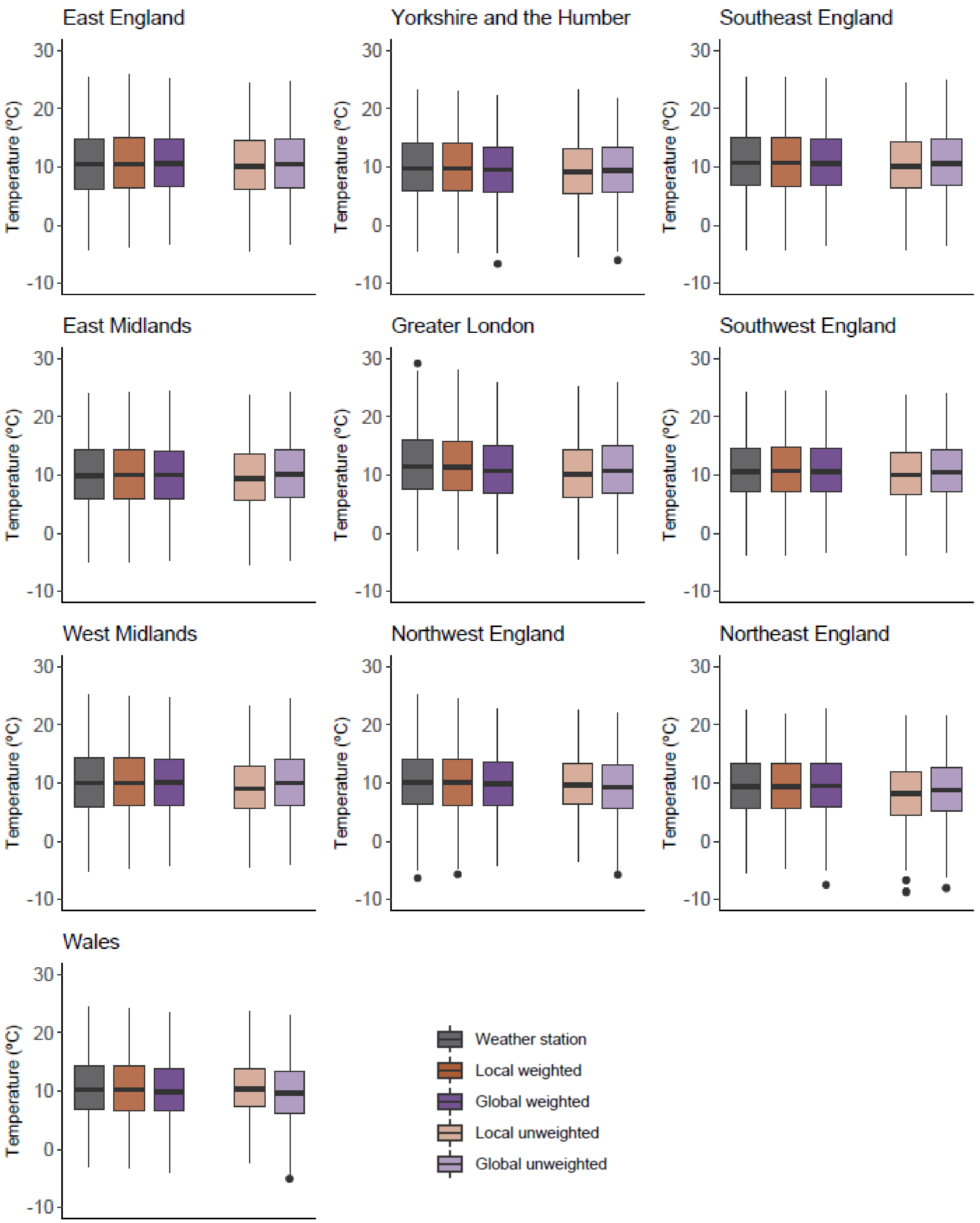

Figure S3. Boxplots for the mean daily temperature distribution by exposure dataset in England and Wales between 1993 and 2006 by region

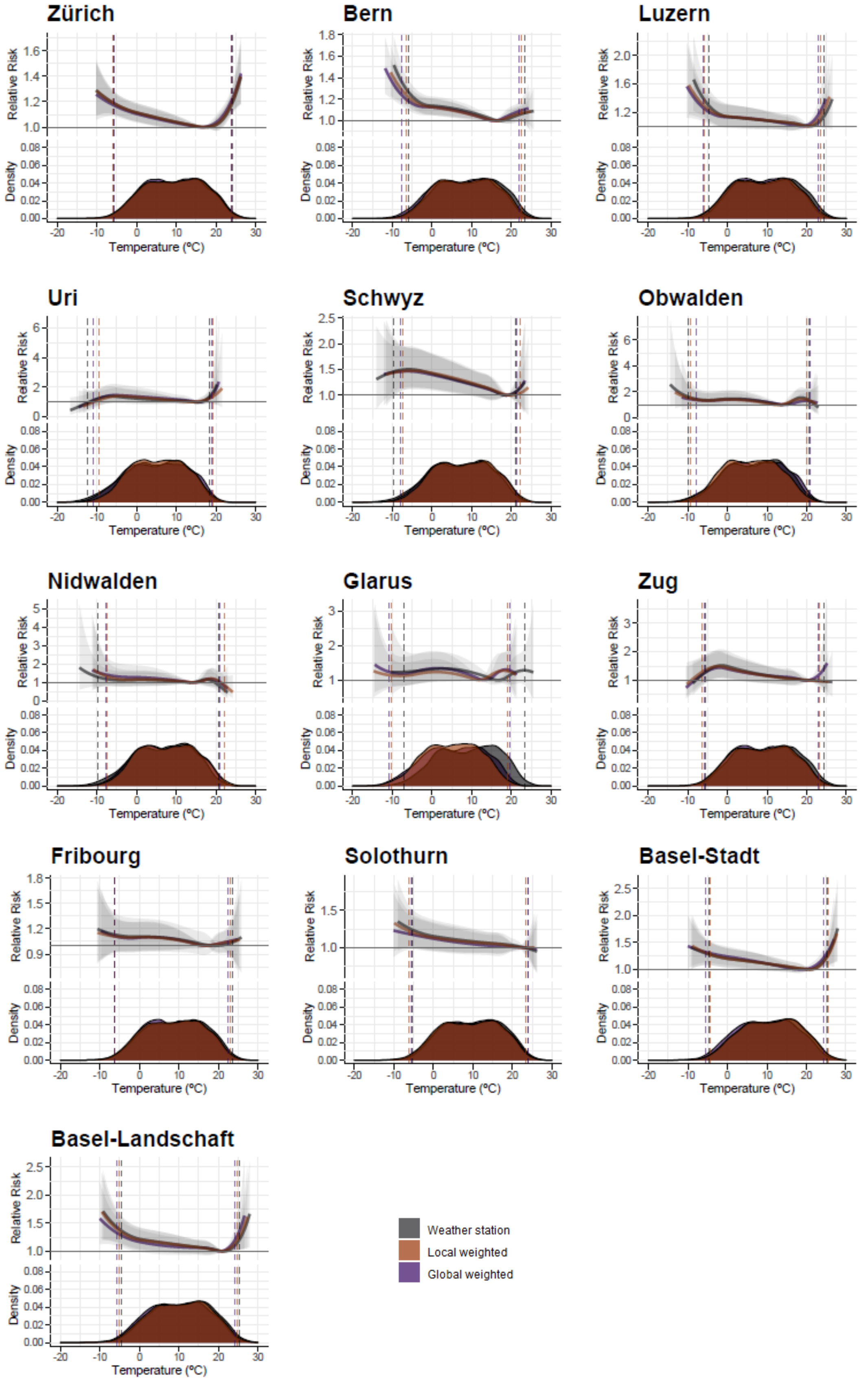

Figure S4. Exposure-response curve for the local and global population-weighted GCD and weather station data by region for Switzerland

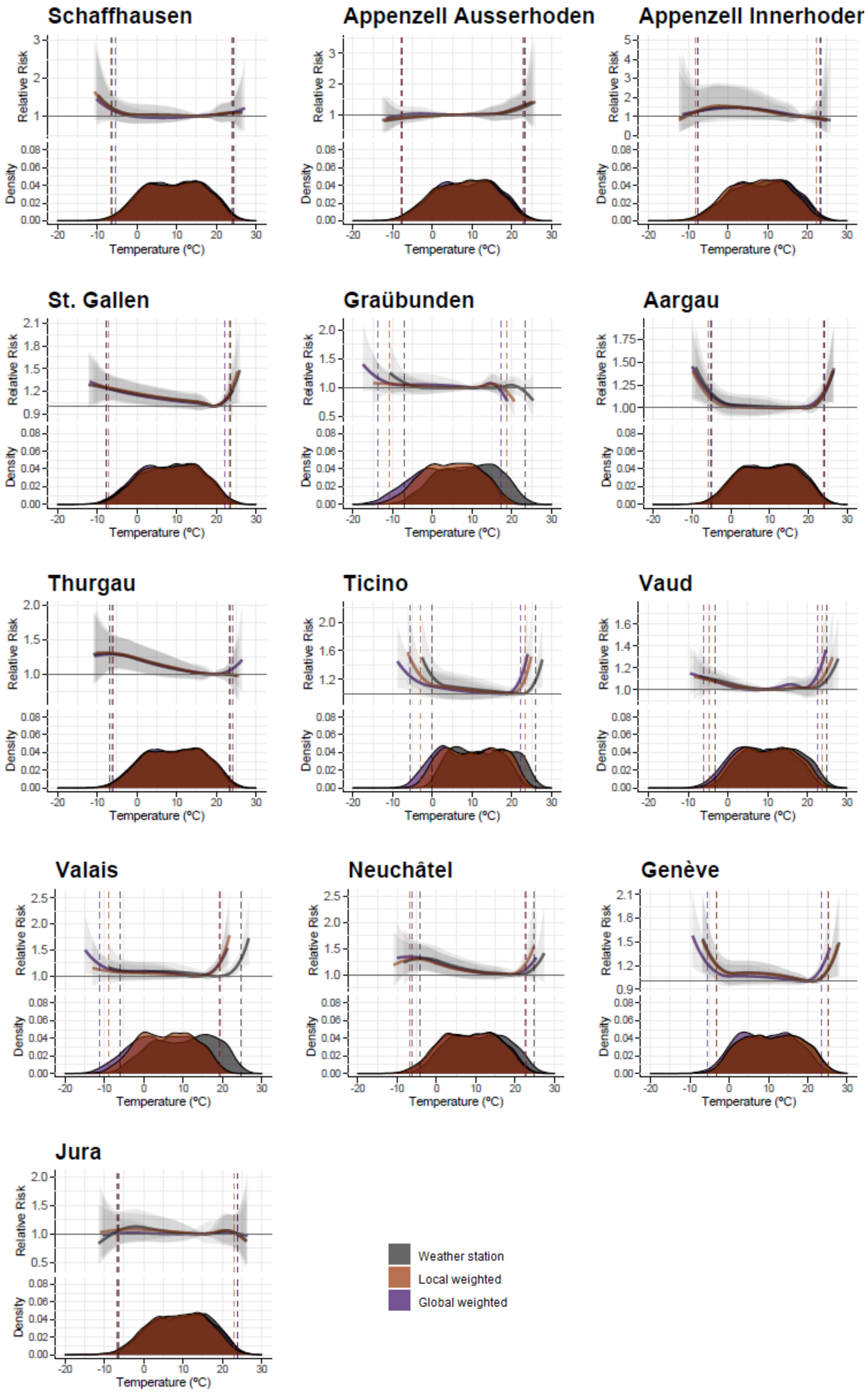

Figure S4. Exposure-response curve for the local and global population-weighted GCD and weather station data by region for Switzerland

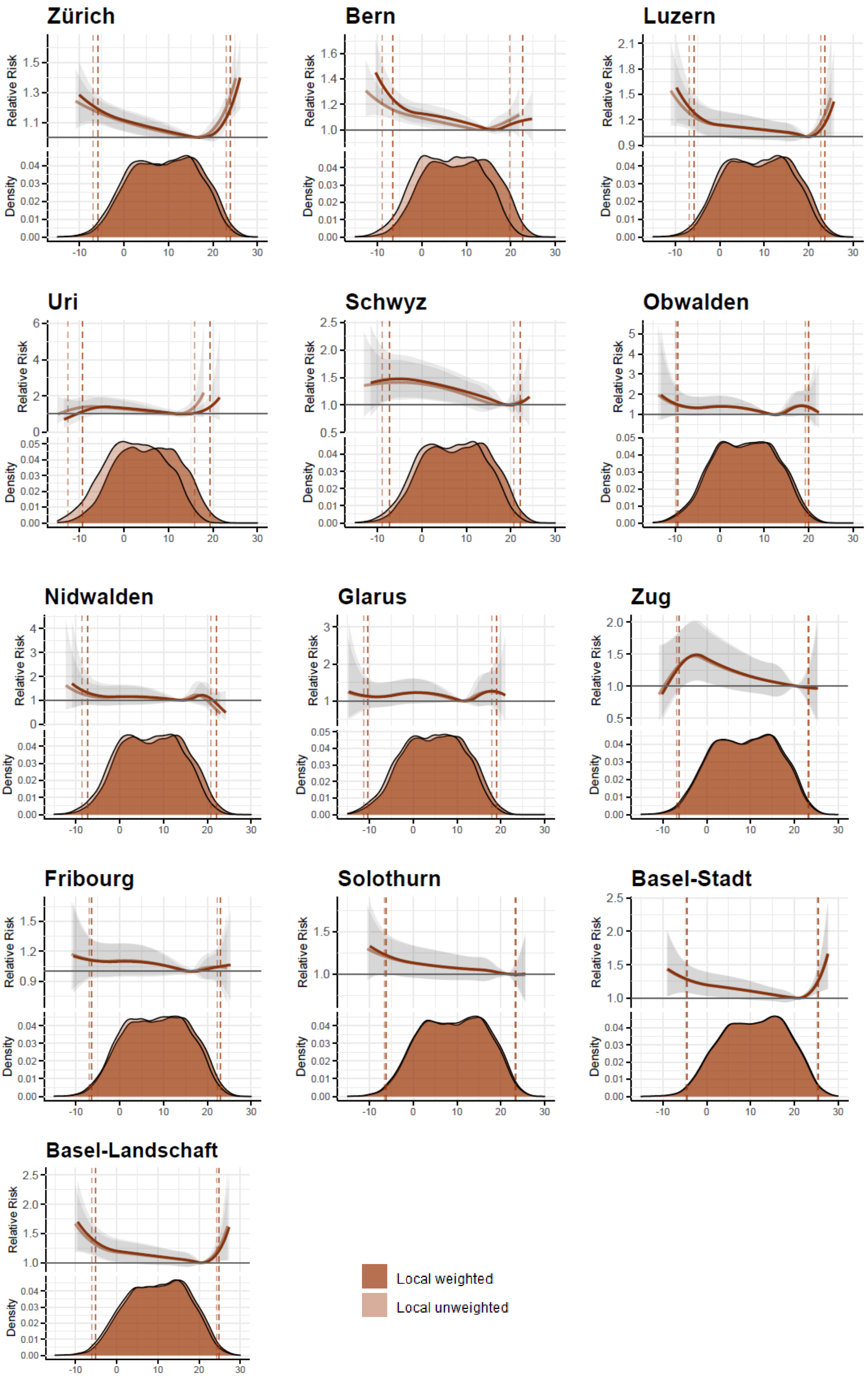

Figure S5. Exposure-response curve for the local population-weighted and unweighted GCD by region for Switzerland

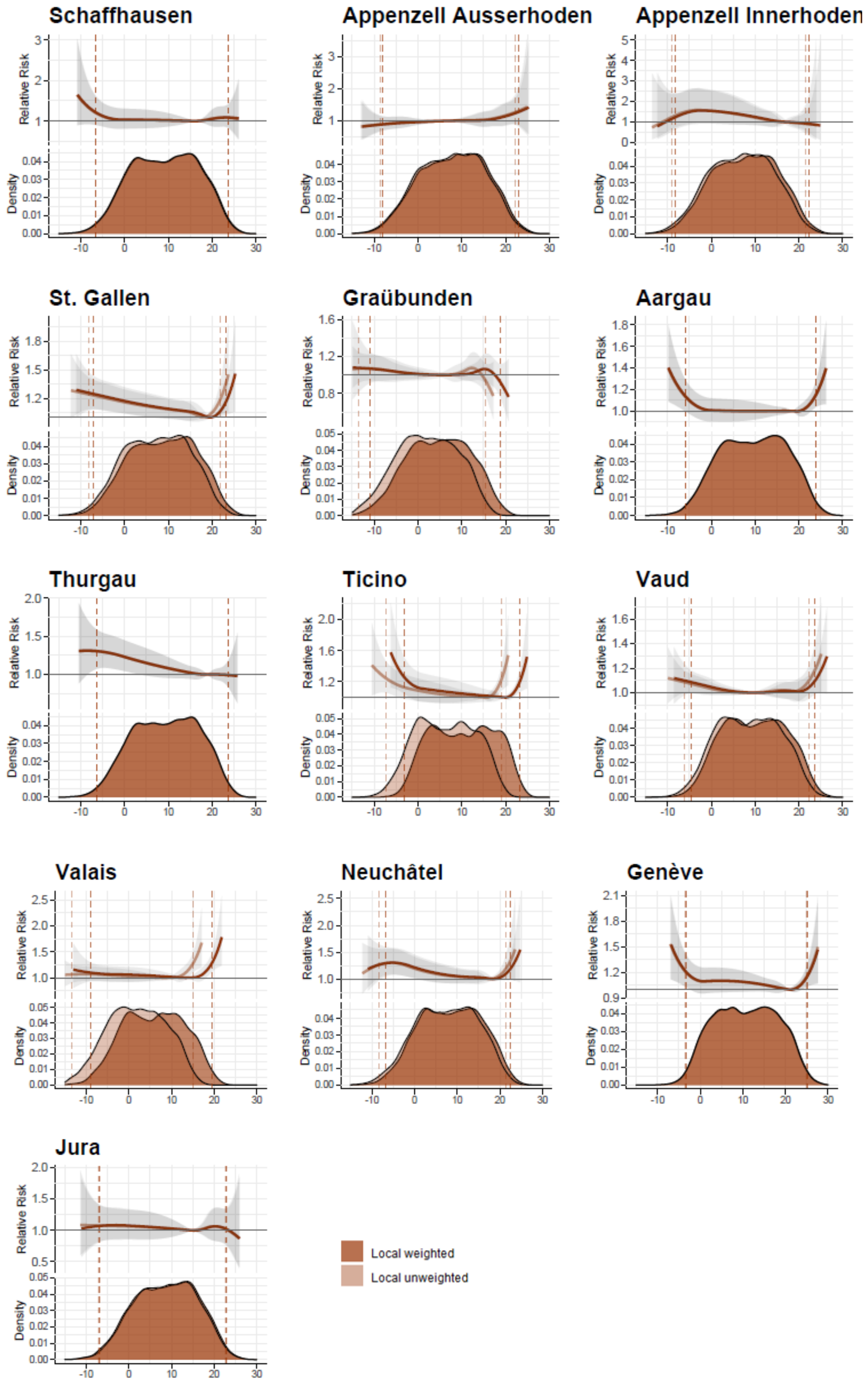

Figure S5. Exposure-response curve for the local population-weighted and unweighted GCD by region for Switzerland

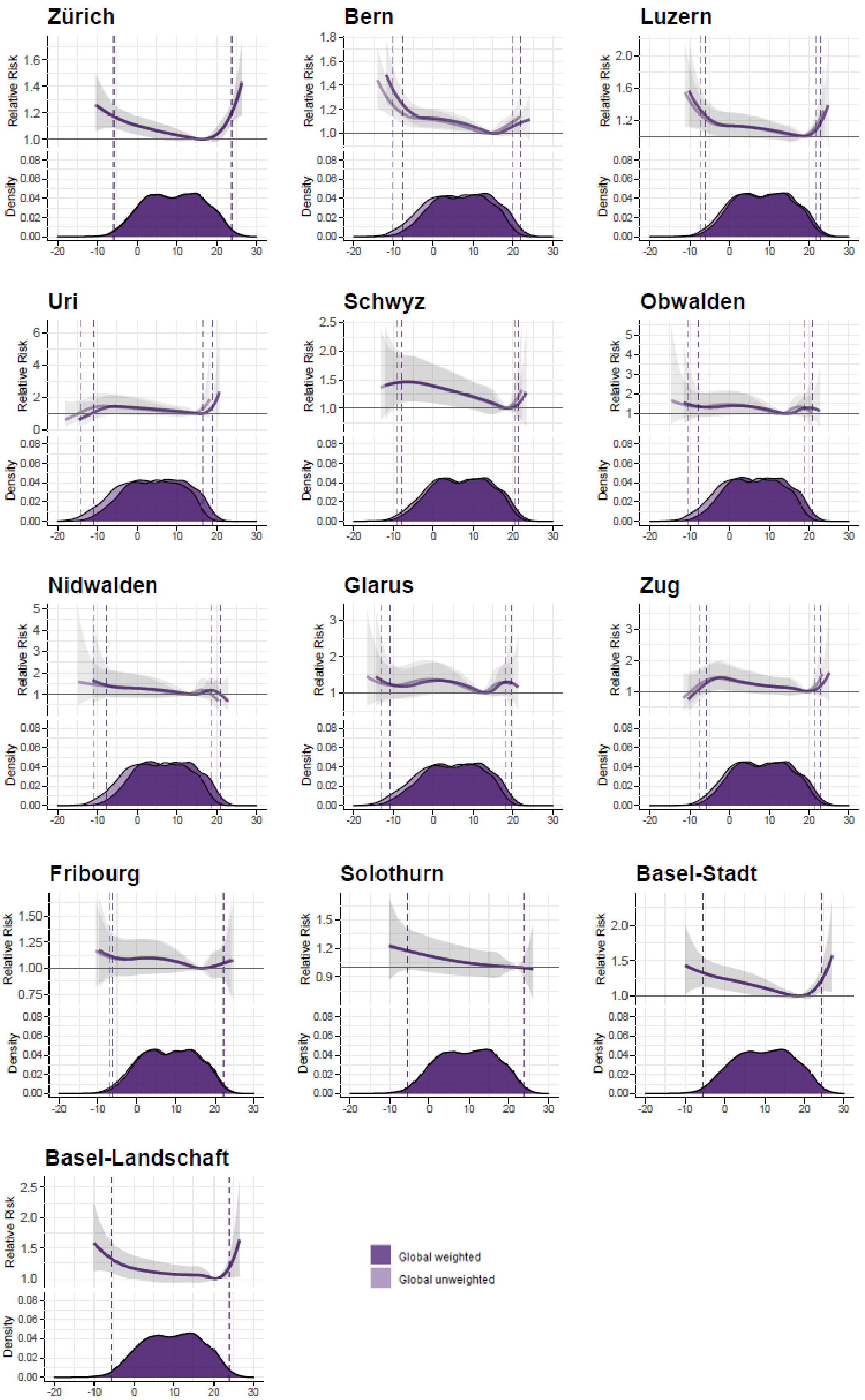

Figure S6. Exposure-response curve for the global population-weighted and unweighted GCD by region for Switzerland

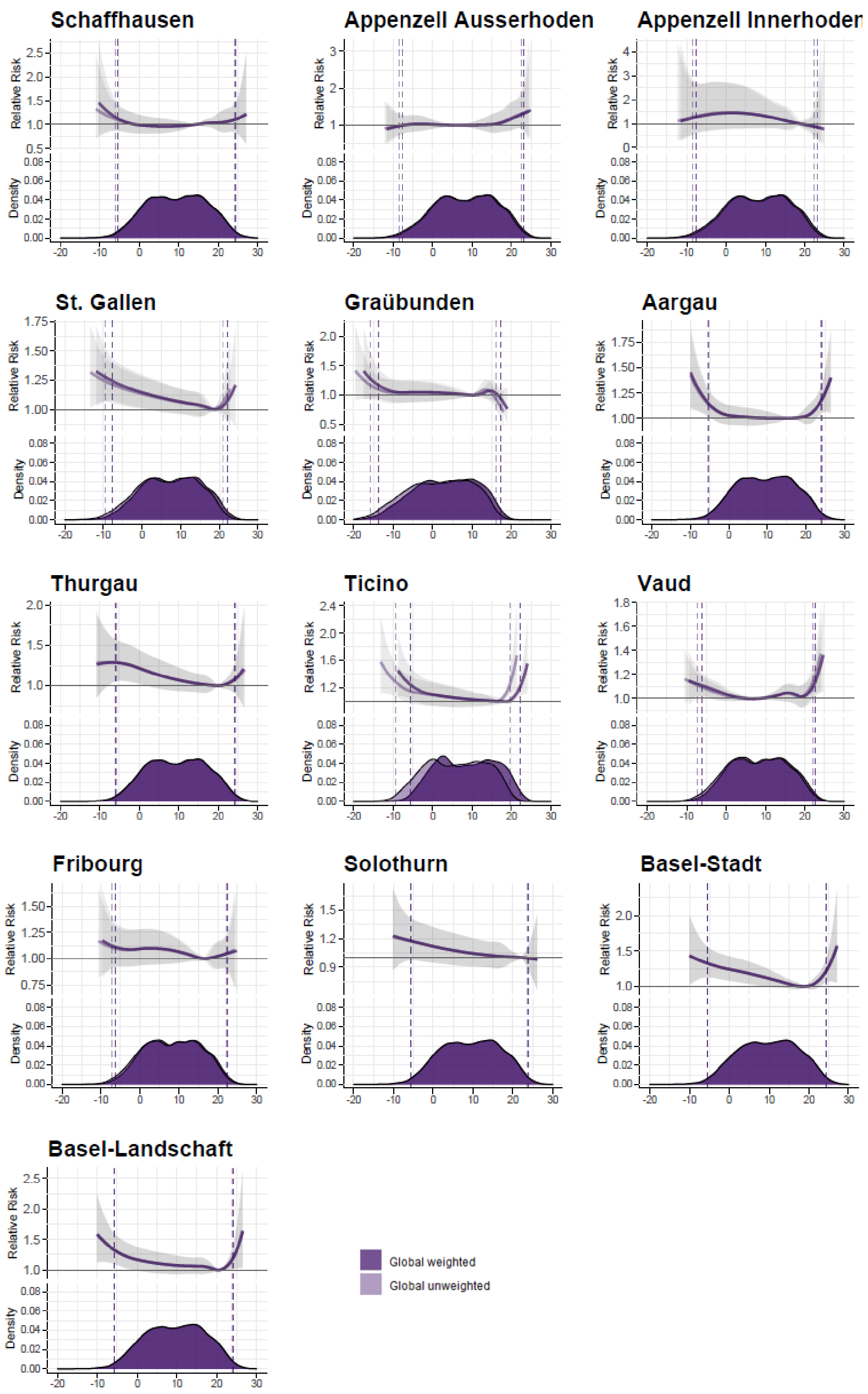

Figure S6. Exposure-response curve for the global population-weighted and unweighted GCD by region for Switzerland

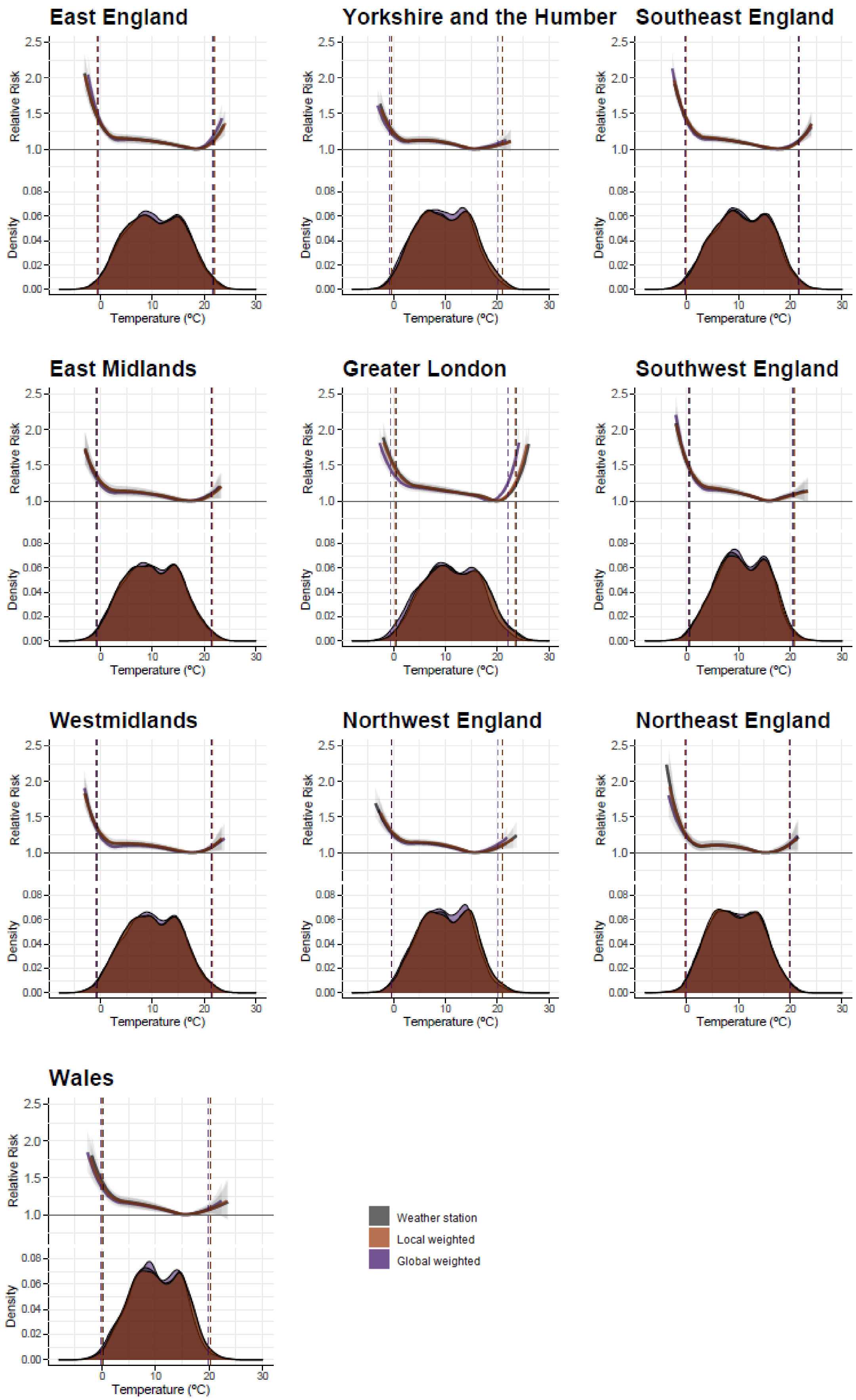

Figure S7. Exposure-response curve for the local and global population-weighted GCD and weather station data for the 10 regions in England and Wales

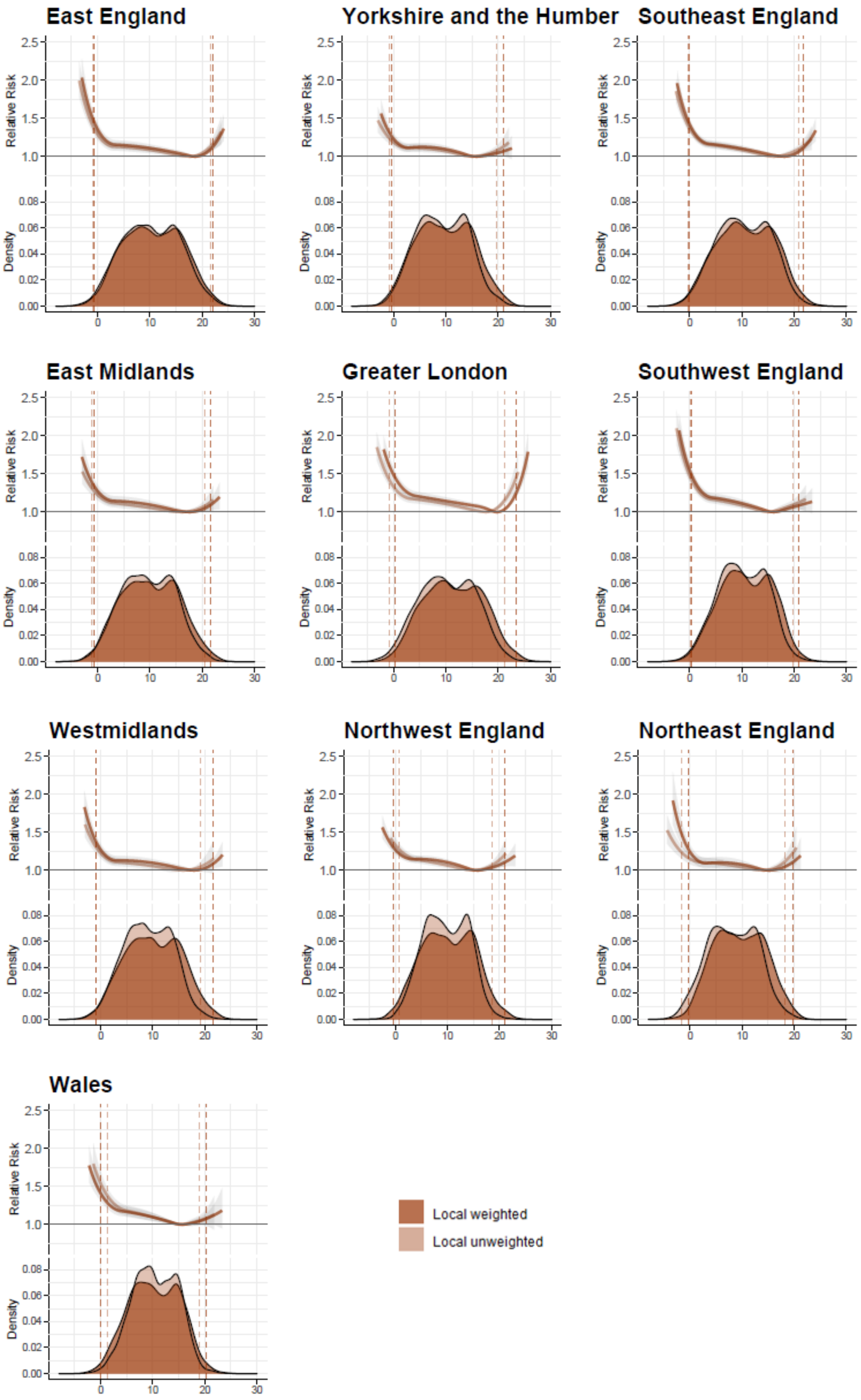

Figure S8. Exposure-response curve for the local population-weighted and unweighted GCD for the 10 regions in England and Wales

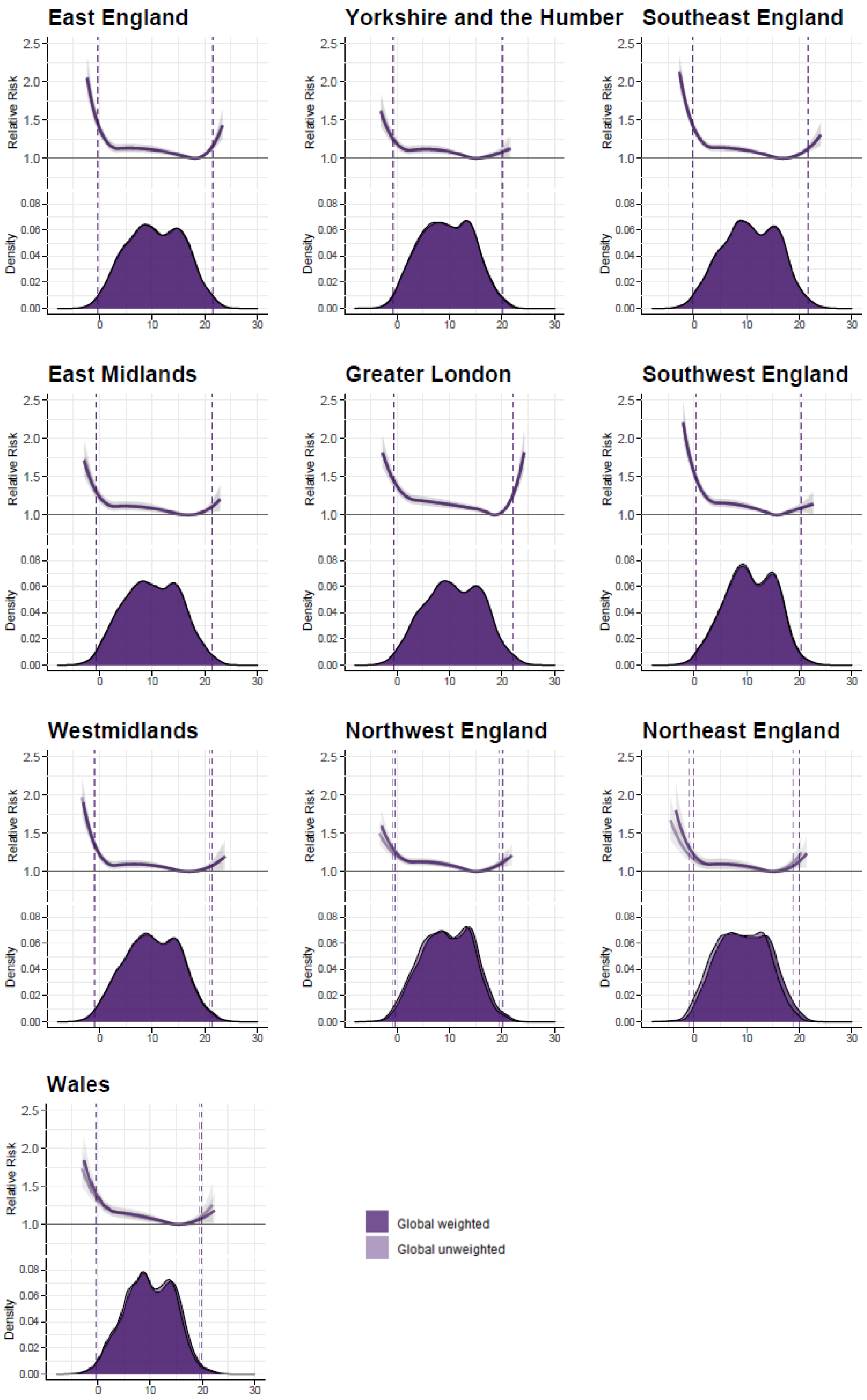

Figure S9. Exposure response curve for the global population-weighted and unweighted GCD and temperature monitor for the 10 regions in England and Wales

Figure S10. Goodness of fit per exposure dataset measured by the qAIC (quasi-Akaike's information criterion) averaged over the 10 regions in England and Wales

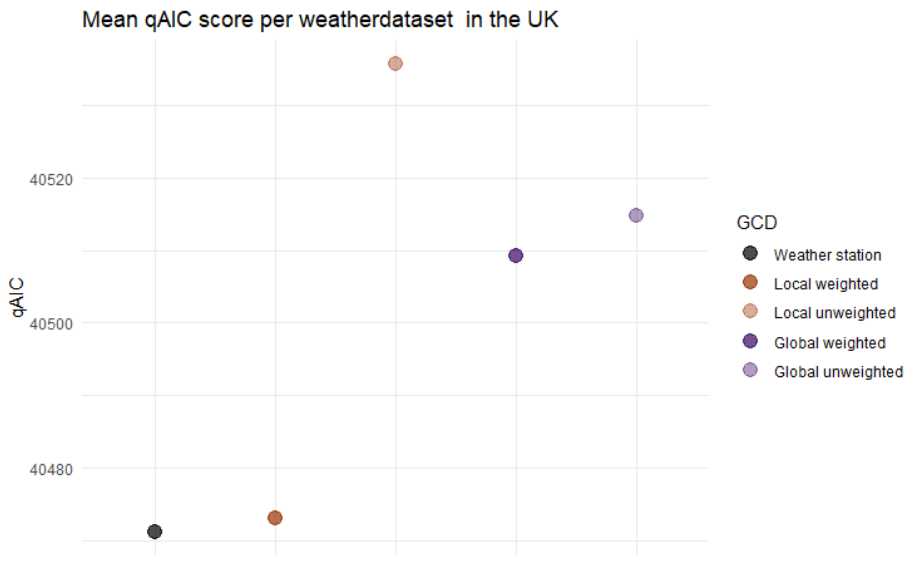

Figure S11. Goodness of fit per exposure dataset measured by the qAIC (quasi-Akaike's information criterion) averaged over the 26 regions in Switzerland

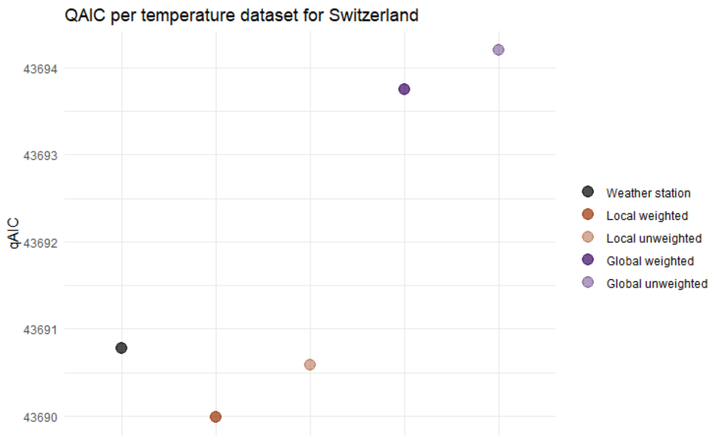

Figure S12. Absolute differences in mortality fractions for heat and cold estimated by the GCD exposure datasets and the weather station, plotted against regional characteristics for Switzerland

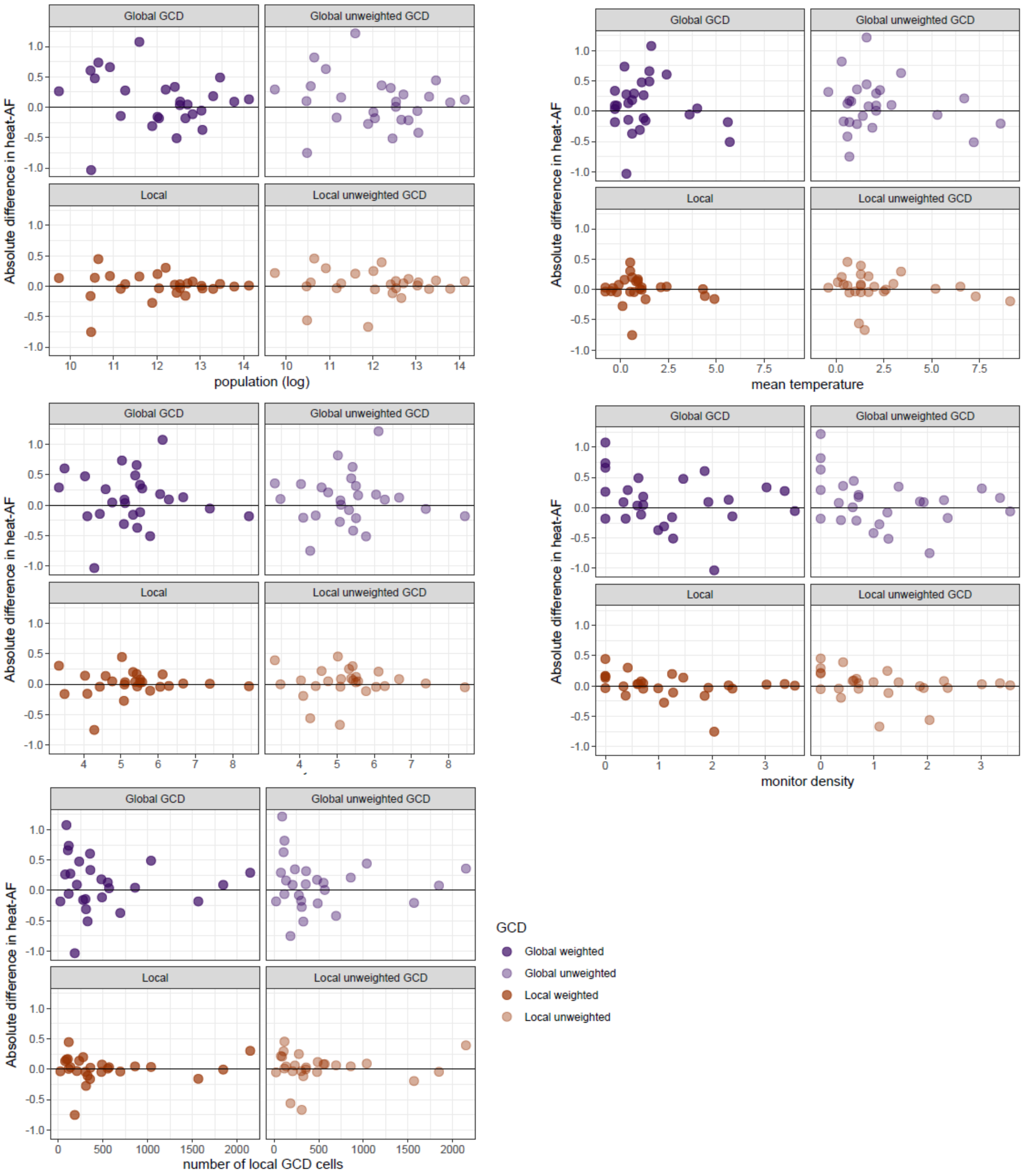

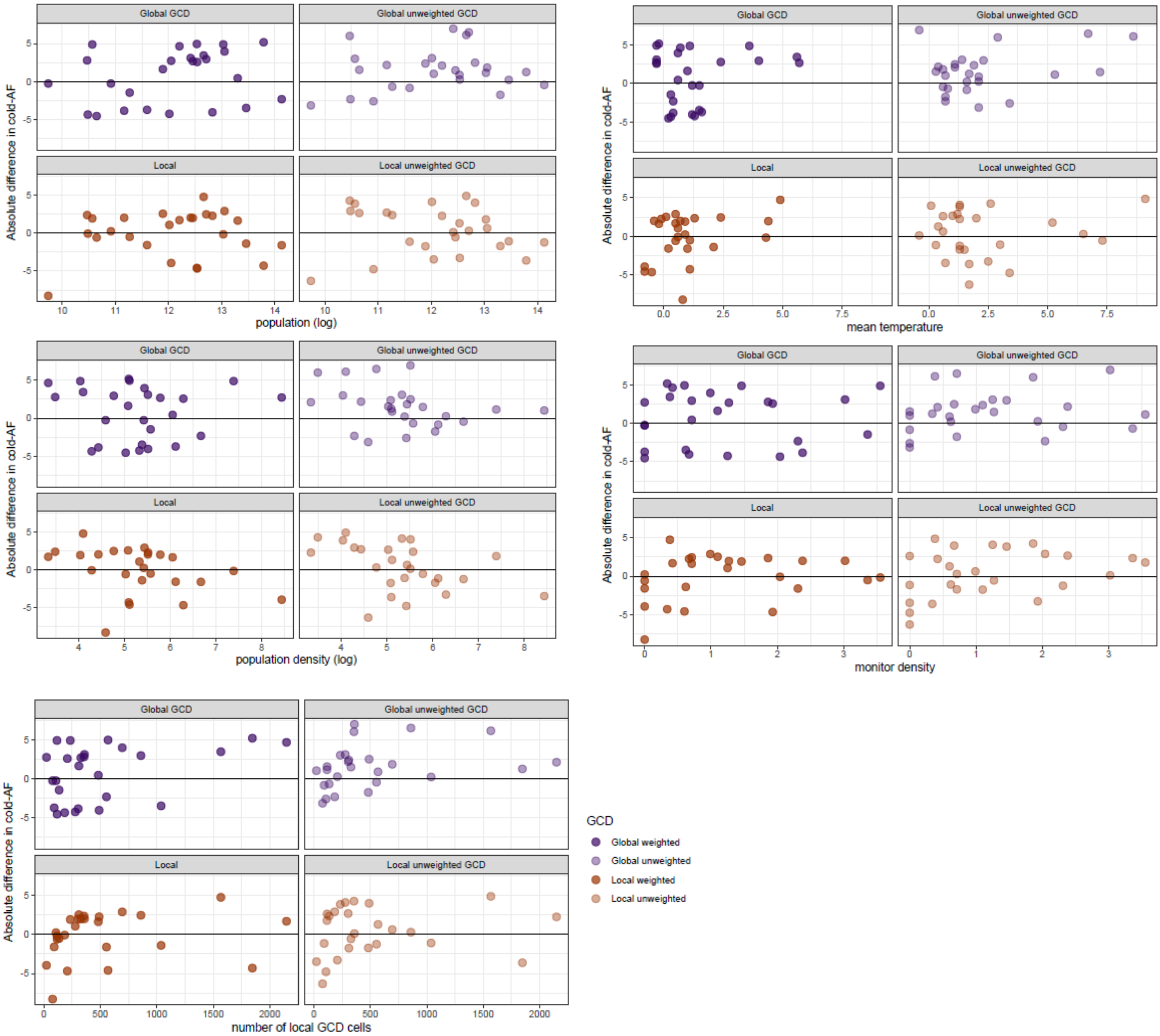
Global GCD
Global unweighted GCD
Local
Local unweighted GCD
Absolute difference in cold-AF
population (log)
mean temperature
population density (log)
monitor density
number of local GCD cells
GCD
Global weighted
Global unweighted
Local weighted
Local unweighted

Figure S13. Absolute relative risk (RR) for heat and cold estimated by the GCD exposure datasets and the weather station, plotted against regional characteristics for Switzerland

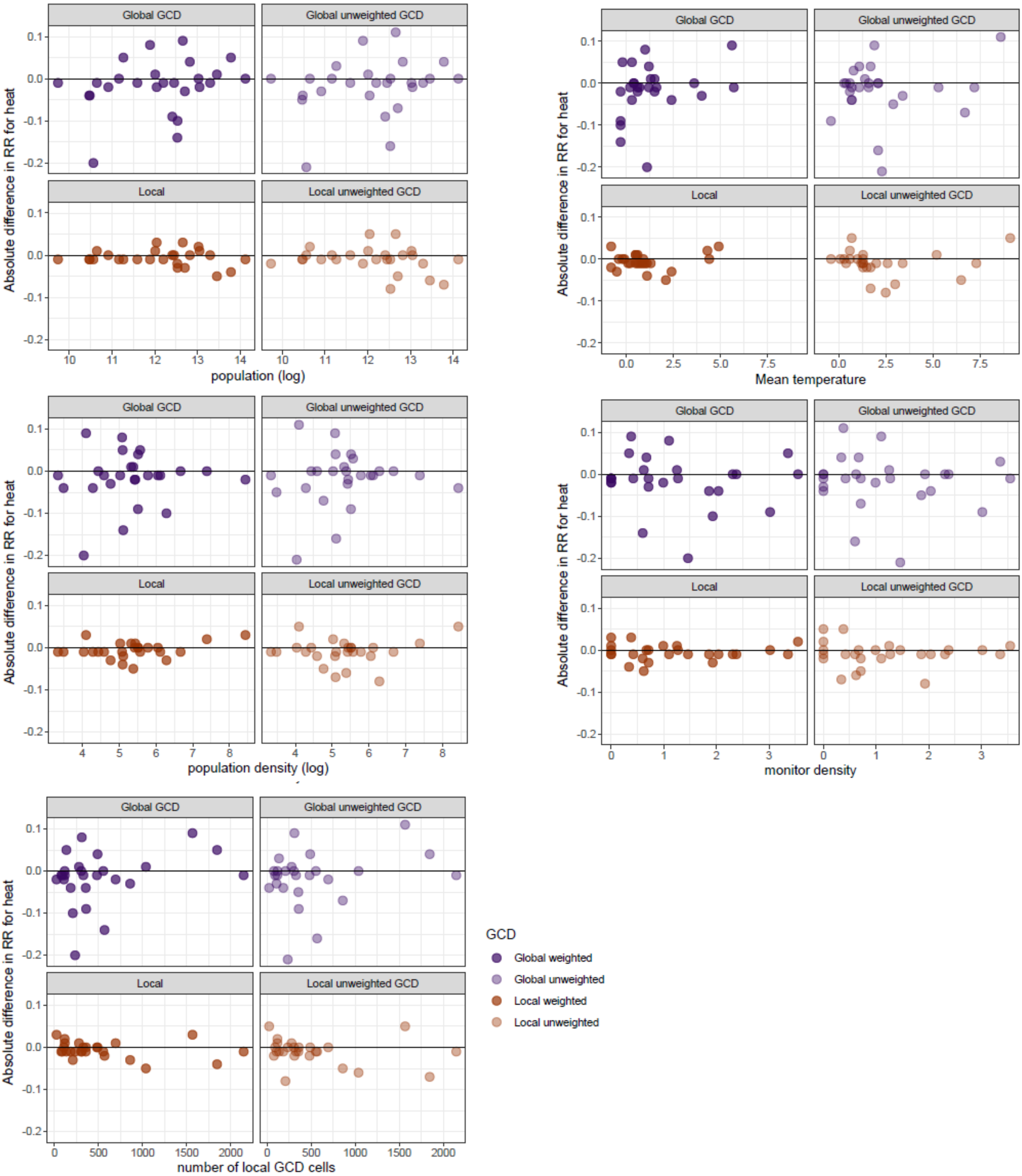

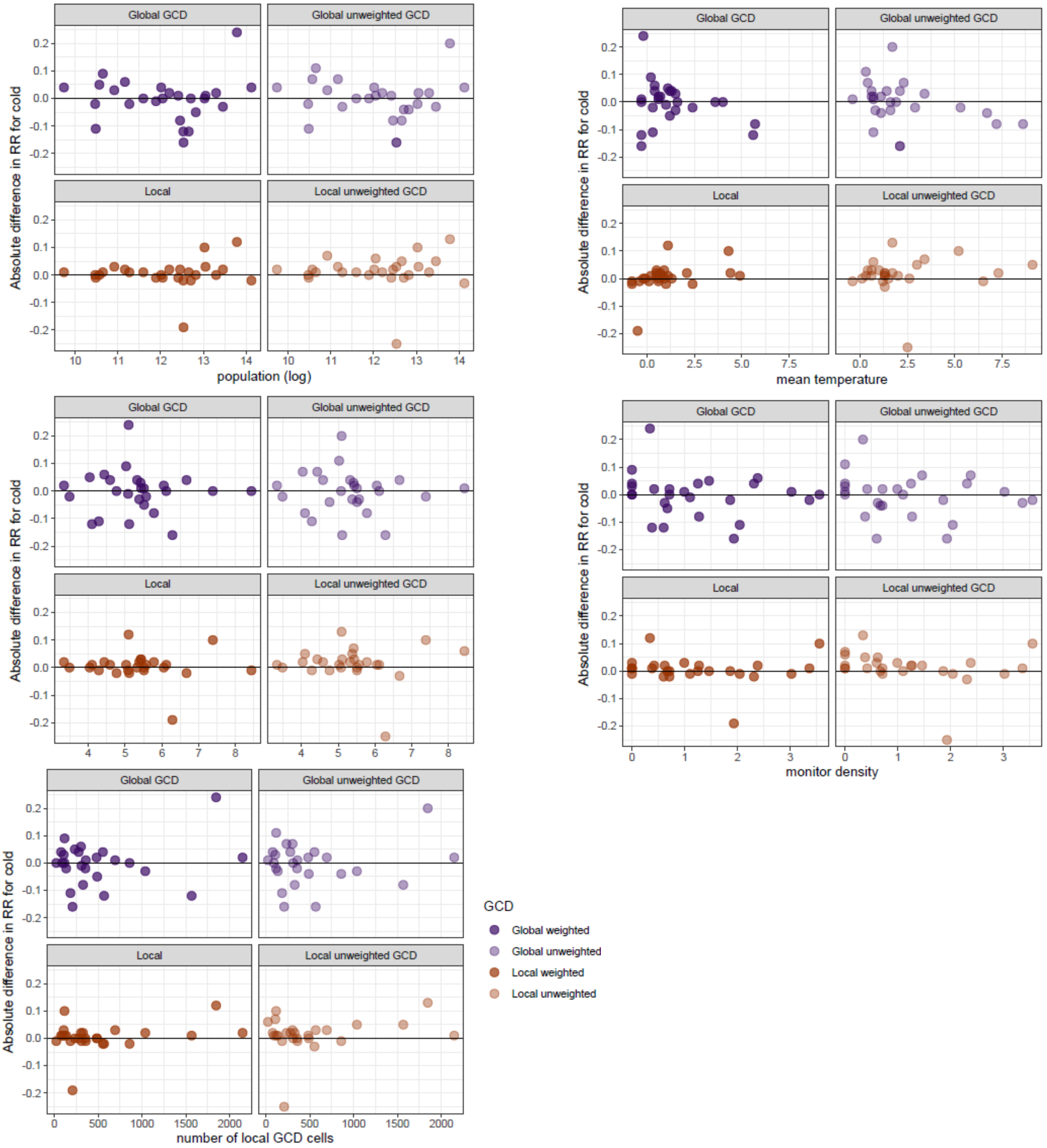
Global GCD
Global unweighted GCD
Local
Local unweighted GCD
Absolute difference in RR for cold
population (log)
mean temperature
monitor density
number of local GCD cells
GCD
Global weighted
Global unweighted
Local weighted
Local unweighted

Figure S14. Absolute differences in mortality fractions for heat and cold estimated by the GCD exposure datasets and the temperature monitor, plotted against regional characteristics for England and Wales

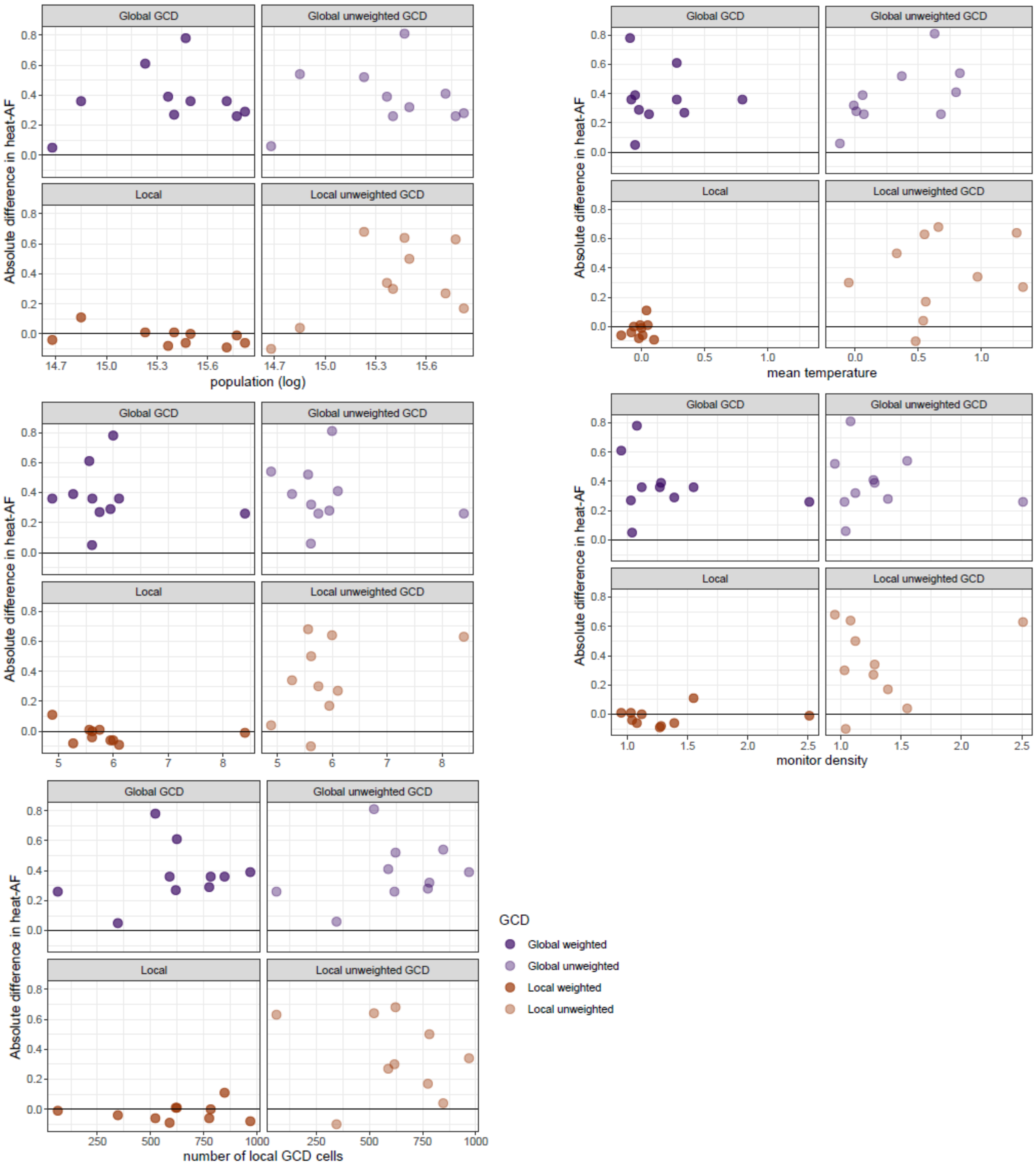

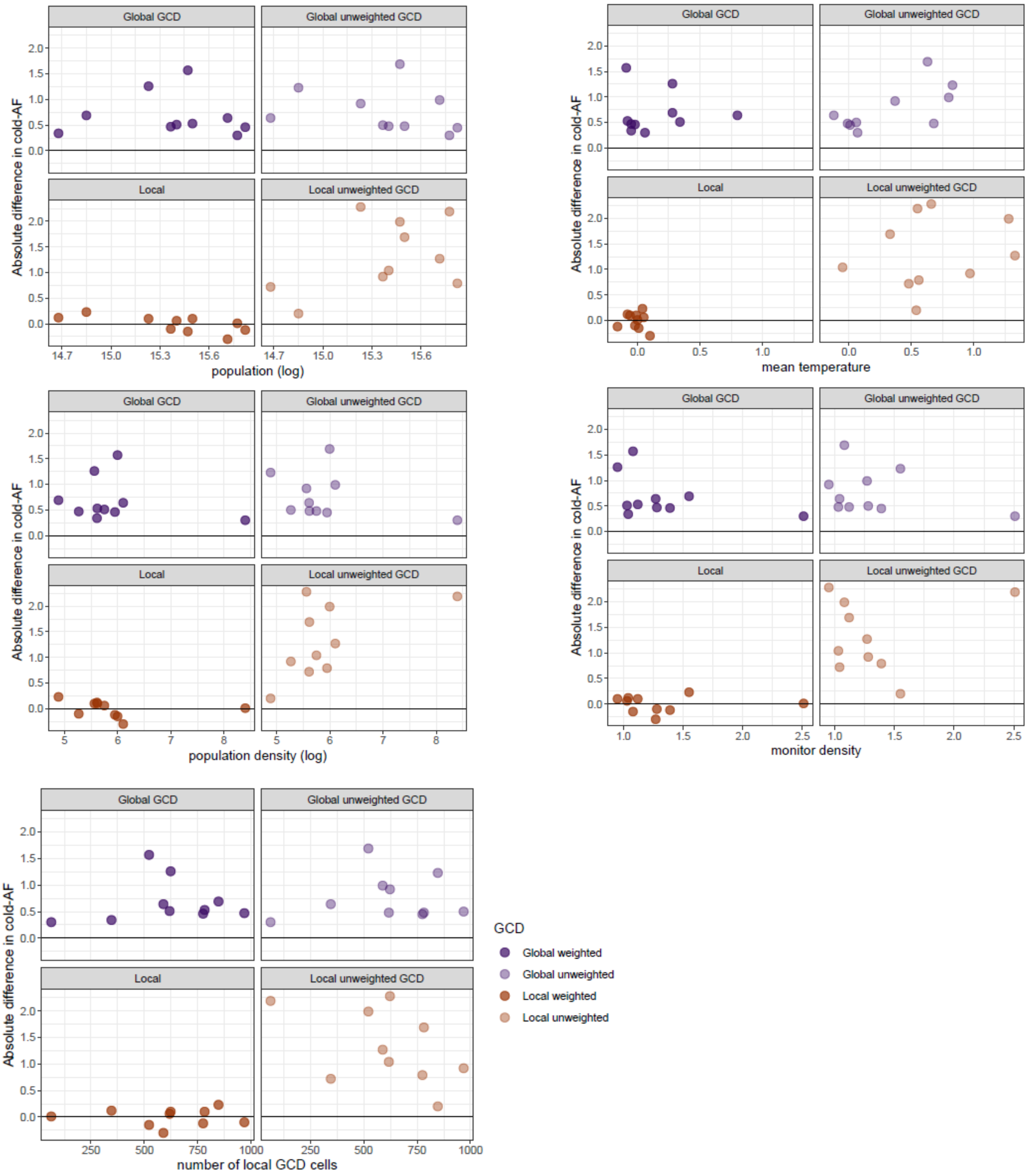

Global GCD
Global unweighted GCD
Local
Local unweighted GCD
Absolute difference in cold-AF
population (log)
mean temperature
population density (log)
monitor density
number of local GCD cells
GCD
Global weighted
Global unweighted
Local weighted
Local unweighted

Figure S15. Absolute differences in relative risk (RR) for heat and cold estimated by the GCD exposure datasets and the weather station, plotted against regional characteristics for England and Wales

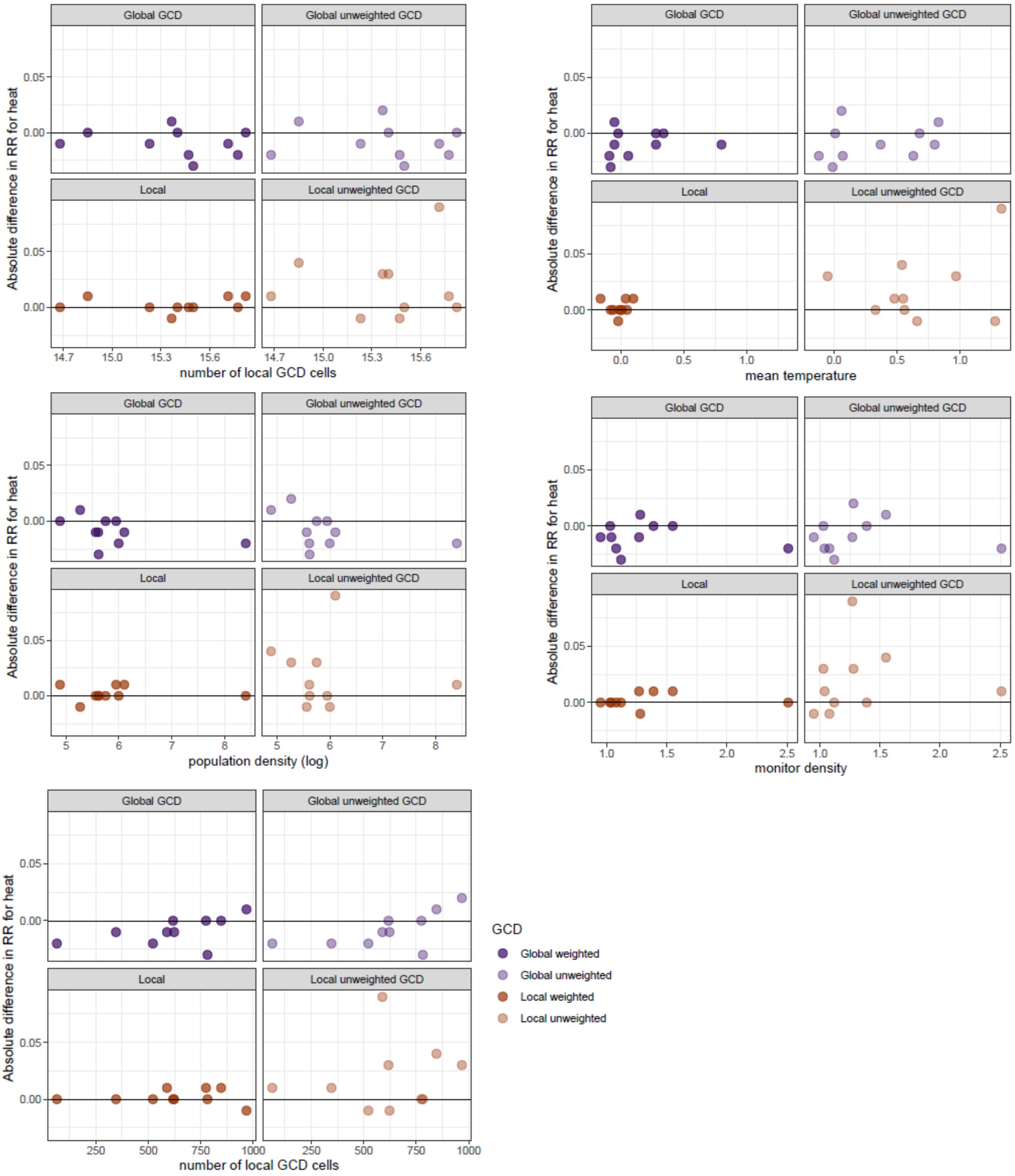

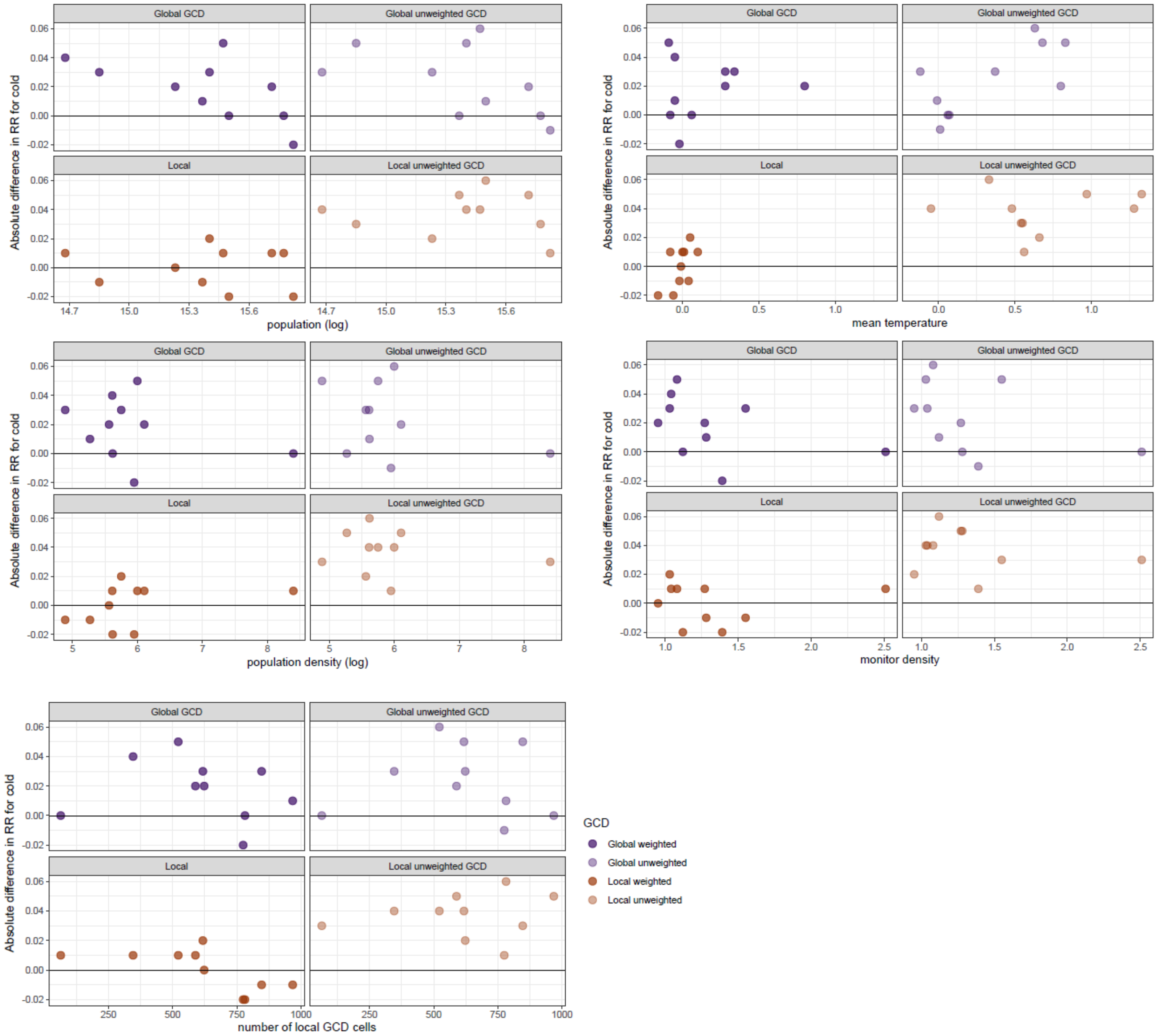
Global GCD
Global unweighted GCD
Local
Local unweighted GCD
Absolute difference in RR for cold
population (log)
mean temperature
population density (log)
monitor density
number of local GCD cells
GCD
Global weighted
Global unweighted
Local weighted
Local unweighted

Table S1

Descriptive statistics of the mortality data and regional-level indicators (area, population) and characteristics of the temperature datasets (weather station, local and global gridded climate datasets (GCD)) in England and Wales (1993-2006) and Switzerland (1989-2017).

Region	Total deaths (N)	Area (km²)	Population density (km²)	Weather stations	Station density (1,000 km²)	Local cells (N)	Global cells (N)
**East England**	**749,380**	19,575	275	32	1.6	782	40
**Eastmidlands**	**606,487**	15,815	260	21	1.3	623	35
**London**	**845,215**	1,595	4,441	5	3.1	64	2
**Northeast England**	**407,862**	8,676	273	37	4.3	346	18
**Northwest England**	**1,069,737**	14,915	447	27	1.8	589	33
**Southeast England**	**1,132,649**	19,412	383	37	1.9	775	37
**Southwest England**	**771,224**	24,293	194	42	1.7	968	52
**Wales**	**472,014**	21,224	133	33	1.6	847	44
**Westmidlands**	**771,704**	13,004	402	26	2.0	522	27
**Yorkshire & the Humber**	**747,424**	15,564	314	22	1.4	618	35
**Overall**	**7,573,716**	154,073	329	282	1.8	6134	323

Canton	Total deaths (N)	Area (km²)	Population density (km²)	Weather stations	Station density (1,000 km²)	Local cells (N)	Global cells (N)
**Aargau**	**121,044**	1,404	425	1	0.7	482	8
**Appenzel Ausserhoden**	**3,981**	243	226	0	0.0	107	5
**Appenzel Innerhoden**	**14,961**	172	98	0	0.0	77	4
**Basel-Land**	**60,618**	518	537	1	1.9	208	5
**Basel-Stadt**	**67,472**	37	4,607	0	0.0	23	2
**Bern**	**267,706**	5,959	163	2	0.3	1,846	25
**Fribourg**	**55,096**	1,671	165	1	0.6	568	10
**Geneve**	**91,455**	282	1,612	1	3.5	116	2
**Glarus**	**11,365**	685	57	1	1.5	234	7
**Graubunden**	**47,486**	7,105	28	3	0.4	2,148	29
**Jura**	**18,948**	839	84	2	2.4	304	7
**Luzern**	**81,882**	1,494	248	1	0.7	489	10
**Neuchatel**	**46,821**	802	205	1	1.2	278	7
**Nidwalden**	**7,779**	276	152	0	0.0	117	4
**Obwalden**	**7,580**	491	72	1	2.0	184	6
**Schaffhausen**	**21,443**	298	262	1	3.4	135	4
**Schwyz**	**28,432**	908	161	1	1.1	310	5
**Solothurn**	**64,368**	790	324	1	1.3	328	9
**St.Gallen**	**109,947**	2,028	229	2	1.0	694	12
**Thurgau**	**53,913**	994	247	3	3.0	359	8
**Ticino**	**82,056**	2,812	117	2	0.7	859	12
**Uri**	**9,070**	1,077	33	2	1.9	355	8
**Valais**	**66,356**	5,225	60	2	0.4	1,567	25
**Vaud**	**156,738**	3,212	218	2	0.6	1,038	16
**Zug**	**19,302**	239	453	0	0.0	91	4
**Zürich**	**306,758**	1,729	788	4	2.3	554	9
**Overall**	**1,822,622**	41,290	188	35	0.8	13,471	243

Table S2. Descriptive statistics of the observed temperature by exposure dataset for the 26 regions in Switzerland, 1989 - 2017

	GCD	Mean	IQR	min	1st	25th	50th	75th	99th	max
Zürich	Weather station	9.8	11.9	-13.1	-5.7	3.9	10.1	15.7	24.2	27.7
Bern	Weather station	9.4	12.1	-14.1	-6.0	3.4	9.6	15.4	23.5	27.3
Luzern	Weather station	10.1	12.1	-11.6	-4.8	4.0	10.2	16.1	24.3	28.0
Uri	Weather station	4.8	11.3	-21.8	-12.4	-0.6	5.1	10.7	18.3	22.3
Schwyz	Weather station	7.1	11.5	-17.7	-9.7	1.4	7.4	12.9	21.2	24.9
Obwalden	Weather station	6.7	11.4	-18.9	-9.8	1.2	7.2	12.6	20.7	24.3
Nidwalden	Weather station	6.7	11.4	-18.9	-9.8	1.2	7.2	12.6	20.7	24.3
Glarus	Weather station	9.2	12.3	-13.7	-7.0	3.1	9.6	15.4	23.3	26.9
Zug	Weather station	9.9	12.1	-12.2	-5.4	3.9	10.1	16.0	24.3	28.0
Fribourg	Weather station	9.3	11.7	-15.0	-6.3	3.5	9.5	15.2	23.6	27.4
Solothurn	Weather station	10.0	11.8	-11.9	-5.2	4.1	10.1	15.9	23.9	27.6
Basel-Stadt	Weather station	10.9	11.4	-12.4	-4.7	5.2	11.2	16.6	25.3	29.2
Basel-Landschaft	Weather station	10.9	11.4	-12.4	-4.7	5.2	11.2	16.6	25.3	29.2
Schaffhausen	Weather station	9.9	12.1	-12.6	-6.2	3.8	10.0	15.9	24.3	27.9
Appenzell Ausserhoden	Weather station	8.7	11.5	-14.9	-7.7	3.0	9.1	14.5	23.5	27.1
Appenzell Innerhoden	Weather station	8.7	11.5	-14.9	-7.7	3.0	9.1	14.5	23.5	27.1
St. Gallen	Weather station	8.7	11.5	-14.9	-7.7	3.0	9.1	14.5	23.5	27.1
Graübunden	Weather station	9.0	12.0	-14.5	-7.0	3.1	9.4	15.1	23.4	26.9
Aargau	Weather station	10.3	11.7	-11.7	-5.0	4.4	10.4	16.1	24.2	28.7
Thurgau	Weather station	9.1	11.9	-13.8	-6.8	3.2	9.3	15.1	23.2	27.2
Ticino	Weather station	12.9	12.0	-5.4	-0.1	6.8	12.8	18.8	26.0	28.8
Vaud	Weather station	11.1	11.6	-10.1	-3.2	5.4	11.2	16.9	24.9	28.8
Valais	Weather station	10.4	13.0	-11.4	-5.9	4.0	11.0	17.0	24.8	28.3
Neuchâtel	Weather station	10.7	11.8	-10.2	-4.2	4.8	10.7	16.6	24.8	28.8
Genève	Weather station	11.1	12.0	-9.2	-3.3	5.0	11.1	17.0	25.3	29.5
Jura	Weather station	9.8	11.5	-17.1	-6.6	4.1	10.1	15.6	23.8	27.6
overall mean		9.4	11.8	-13.6	-6.3	3.6	9.7	15.4	23.6	27.4
		Mean	IQR	min	1st	25th	50th	75th	99th	max
Zürich	Local-weighted	9.6	11.8	-13.2	-5.9	3.7	9.8	15.5	24.0	27.7
Bern	Local-weighted	8.6	11.8	-14.6	-6.6	2.7	8.7	14.5	22.6	26.3
Luzern	Local-weighted	9.2	11.9	-13.0	-5.8	3.3	9.4	15.2	23.6	27.3
Uri	Local-weighted	5.5	10.7	-17.6	-9.4	0.2	5.6	10.9	19.3	22.9
Schwyz	Local-weighted	8.0	11.5	-14.9	-7.3	2.3	8.2	13.8	22.2	25.6
Obwalden	Local-weighted	6.0	10.8	-18.2	-9.4	0.6	6.1	11.4	20.1	23.5
Nidwalden	Local-weighted	7.8	11.3	-15.2	-7.4	2.2	8.0	13.5	22.1	25.5
Glarus	Local-weighted	5.1	10.8	-19.4	-10.4	-0.2	5.3	10.6	19.0	21.8
Zug	Local-weighted	9.0	11.8	-13.5	-6.3	3.1	9.2	14.9	23.3	26.9
Fribourg	Local-weighted	8.8	11.6	-14.7	-6.4	3.0	9.0	14.7	23.0	26.9
Solothurn	Local-weighted	9.3	11.9	-13.7	-6.2	3.4	9.4	15.3	23.5	27.1
Basel-Stadt	Local-weighted	11.0	11.4	-12.1	-4.5	5.4	11.3	16.8	25.4	29.3

Basel-Landschaft	Local-weighted	10.3	11.4	-12.8	-5.2	4.7	10.6	16.1	24.7	28.7
Schaffhausen	Local-weighted	9.4	12.1	-13.0	-6.5	3.3	9.5	15.4	23.8	27.3
Appenzell Ausserhoden	Local-weighted	8.1	11.5	-15.4	-8.0	2.5	8.5	13.9	22.9	26.4
Appenzell Innerhoden	Local-weighted	7.8	11.3	-15.9	-8.2	2.1	8.0	13.5	22.4	25.9
St. Gallen	Local-weighted	8.8	11.6	-14.4	-7.1	3.0	9.0	14.6	23.3	26.9
Graübunden	Local-weighted	4.8	11.0	-19.3	-10.9	-0.6	5.0	10.4	18.7	21.4
Aargau	Local-weighted	9.7	11.9	-12.7	-5.8	3.7	9.9	15.6	23.9	27.9
Thurgau	Local-weighted	9.5	12.0	-12.8	-6.2	3.5	9.7	15.5	23.6	27.6
Ticino	Local-weighted	10.3	12.2	-9.9	-3.1	4.2	10.4	16.4	23.3	25.7
Vaud	Local-weighted	9.8	11.5	-12.0	-4.6	4.1	9.9	15.6	23.7	27.2
Valais	Local-weighted	5.9	11.2	-16.0	-8.8	0.3	6.1	11.5	19.6	23.0
Neuchâtel	Local-weighted	8.5	11.4	-13.5	-6.8	2.8	8.6	14.2	22.4	26.0
Genève	Local-weighted	10.9	11.9	-9.3	-3.3	4.9	10.9	16.8	25.2	29.3
Jura	Local-weighted	8.9	11.1	-15.9	-6.7	3.4	9.1	14.5	22.9	26.9
overall mean		8.5	11.5	-14.3	-6.8	2.8	8.7	14.3	22.6	26.2
		Mean	IQR	min	1st	25th	50th	75th	99th	max
Zürich	Local-unweighted	8.7	11.7	-14.5	-6.9	2.8	8.8	14.5	23.1	26.7
Bern	Local-unweighted	6.1	11.0	-17.1	-9.0	0.6	6.2	11.6	19.8	23.4
Luzern	Local-unweighted	8.3	11.5	-14.4	-6.9	2.6	8.5	14.1	22.7	26.4
Uri	Local-unweighted	2.5	10.1	-21.5	-12.7	-2.4	2.6	7.7	15.9	19.2
Schwyz	Local-unweighted	6.6	11.0	-17.1	-9.0	1.1	6.7	12.1	20.6	23.9
Obwalden	Local-unweighted	5.5	10.7	-18.9	-9.8	0.1	5.5	10.9	19.3	22.9
Nidwalden	Local-unweighted	6.7	11.0	-17.0	-8.7	1.2	6.8	12.2	20.8	24.2
Glarus	Local-unweighted	4.2	10.5	-20.5	-11.2	-0.9	4.4	9.6	17.9	20.7
Zug	Local-unweighted	8.7	11.7	-14.5	-6.9	2.8	8.8	14.5	23.1	26.7
Fribourg	Local-unweighted	8.1	11.4	-15.0	-7.0	2.5	8.2	13.8	22.2	26.1
Solothurn	Local-unweighted	9.0	11.7	-14.2	-6.5	3.2	9.1	14.9	23.3	26.9
Basel-Stadt	Local-unweighted	10.9	11.4	-12.3	-4.6	5.2	11.1	16.6	25.3	29.2
Basel-Landschaft	Local-unweighted	9.7	11.4	-13.5	-6.0	4.0	9.9	15.4	24.2	28.1
Schaffhausen	Local-unweighted	9.2	12.1	-13.1	-6.6	3.1	9.4	15.2	23.6	27.0
Appenzell Ausserhoden	Local-unweighted	7.7	11.3	-16.2	-8.5	2.1	7.9	13.4	22.3	25.9
Appenzell Innerhoden	Local-unweighted	6.9	11.1	-17.4	-9.0	1.4	7.1	12.4	21.5	25.0
St. Gallen	Local-unweighted	7.4	11.4	-16.4	-8.2	1.8	7.6	13.1	21.8	25.1
Graübunden	Local-unweighted	2.0	10.6	-21.6	-13.6	-3.1	2.1	7.5	15.5	18.4
Aargau	Local-unweighted	9.6	11.8	-12.7	-5.8	3.7	9.8	15.6	23.9	27.8

Thurgau	Local-unweighted	9.4	12.0	-12.9	-6.3	3.5	9.7	15.5	23.6	27.6
Ticino	Local-unweighted	6.3	10.9	-12.8	-7.3	0.8	6.3	11.8	19.1	22.2
Vaud	Local-unweighted	8.6	11.2	-13.4	-6.1	2.9	8.6	14.2	22.4	25.9
Valais	Local-unweighted	1.9	10.4	-20.1	-13.4	-3.2	1.9	7.1	15.1	18.5
Neuchâtel	Local-unweighted	7.6	11.1	-14.9	-8.3	2.1	7.7	13.2	21.4	25.1
Genève	Local-unweighted	10.8	11.8	-9.4	-3.4	4.8	10.8	16.7	25.0	29.0
Jura	Local-unweighted	8.5	11.1	-15.7	-7.1	3.1	8.8	14.1	22.8	26.7
overall mean		7.3	11.2	-15.6	-8.0	1.8	7.5	13.0	21.4	24.9
		Mean	IQR	min	1st	25th	50th	75th	99th	max
Zürich	Global-weighted	9.5	11.6	-13.0	-5.6	3.6	9.5	15.2	23.8	27.6
Bern	Global-weighted	7.9	11.7	-15.0	-7.8	2.2	8.1	13.9	22.0	25.2
Luzern	Global-weighted	8.9	11.4	-12.6	-6.0	3.2	9.0	14.6	22.8	25.9
Uri	Global-weighted	5.2	11.9	-17.5	-10.8	-0.6	5.4	11.3	19.0	22.5
Schwyz	Global-weighted	7.6	11.5	-15.8	-7.8	1.9	7.7	13.4	21.5	24.7
Obwalden	Global-weighted	7.2	11.4	-14.1	-8.0	1.6	7.4	13.0	20.9	24.3
Nidwalden	Global-weighted	7.4	11.3	-13.8	-7.7	1.8	7.5	13.2	21.1	24.5
Glarus	Global-weighted	5.7	11.9	-18.0	-10.8	0.0	6.0	11.8	19.7	22.8
Zug	Global-weighted	8.9	11.4	-13.5	-5.9	3.2	9.0	14.6	22.9	26.1
Fribourg	Global-weighted	8.7	11.4	-13.4	-6.3	3.1	8.8	14.5	22.5	25.9
Solothurn	Global-weighted	9.6	11.5	-13.5	-5.7	3.8	9.7	15.4	23.8	27.2
Basel-Stadt	Global-weighted	9.9	11.4	-13.8	-5.6	4.2	10.0	15.6	24.3	28.2
Basel-Landschaft	Global-weighted	9.7	11.5	-13.6	-5.7	4.0	9.9	15.4	24.1	27.6
Schaffhausen	Global-weighted	9.7	11.7	-12.6	-5.5	3.8	9.8	15.5	24.3	29.1
Appenzell Ausserhoden	Global-weighted	8.7	11.9	-14.7	-7.7	2.8	8.8	14.7	23.1	25.9
Appenzell Innerhoden	Global-weighted	8.7	11.9	-14.6	-7.6	2.8	8.8	14.7	23.1	26.0
St. Gallen	Global-weighted	8.0	11.8	-15.4	-7.9	2.2	8.1	14.0	22.2	25.2
Graübunden	Global-weighted	3.3	12.4	-20.6	-13.7	-2.6	3.7	9.8	17.3	20.2
Aargau	Global-weighted	9.7	11.5	-12.1	-5.2	4.0	9.8	15.4	24.0	27.7
Thurgau	Global-weighted	9.5	11.8	-13.5	-6.0	3.6	9.6	15.4	24.1	27.0
Ticino	Global-weighted	8.7	12.4	-13.9	-5.5	2.6	8.8	14.9	22.2	24.8
Vaud	Global-weighted	8.7	11.4	-13.0	-6.1	3.0	8.8	14.4	22.5	25.9
Valais	Global-weighted	5.1	12.2	-17.5	-11.4	-0.8	5.4	11.4	19.3	22.1
Neuchâtel	Global-weighted	9.0	11.3	-12.6	-6.3	3.3	9.2	14.6	22.9	26.7
Genève	Global-weighted	9.4	11.4	-12.8	-5.4	3.7	9.5	15.1	23.4	27.3
Jura	Global-weighted	9.5	11.4	-14.7	-6.3	3.8	9.7	15.2	23.7	27.7
overall mean		8.2	11.7	-14.5	-7.2	2.5	8.4	14.1	22.3	25.7
			IQR	min	1st	25th	50th	75th	99th	max
Zürich	Global-unweighted	9.3	11.6	-13.4	-6.0	3.4	9.4	15.1	23.7	27.4
Bern	Global-unweighted	5.9	11.8	-17.1	-10.4	0.1	6.2	11.9	20.0	23.0

Luzern	Global-unweighted	8.0	11.5	-13.6	-7.2	2.3	8.1	13.8	21.8	24.9
Uri	Global-unweighted	2.7	12.3	-20.7	-14.1	-3.3	3.0	9.0	16.7	19.9
Schwyz	Global-unweighted	6.5	11.7	-16.7	-9.2	0.8	6.7	12.5	20.4	23.7
Obwalden	Global-unweighted	5.3	11.7	-16.9	-10.4	-0.5	5.5	11.2	19.0	22.5
Nidwalden	Global-unweighted	4.9	11.8	-17.2	-10.8	-0.8	5.1	11.0	18.7	22.4
Glarus	Global-unweighted	4.0	12.1	-19.9	-13.1	-1.9	4.3	10.3	18.1	21.4
Zug	Global-unweighted	7.7	11.5	-14.9	-7.4	2.0	7.8	13.5	21.6	25.0
Fribourg	Global-unweighted	8.2	11.5	-14.3	-7.2	2.5	8.4	14.0	22.1	25.5
Solothurn	Global-unweighted	9.5	11.5	-13.7	-5.7	3.8	9.7	15.3	23.7	27.0
Basel-Stadt	Global-unweighted	9.9	11.4	-13.7	-5.6	4.2	10.1	15.6	24.3	28.1
Basel-Landschaft	Global-unweighted	9.6	11.5	-13.6	-5.8	3.9	9.8	15.4	24.0	27.5
Schaffhausen	Global-unweighted	9.5	11.8	-13.2	-6.1	3.6	9.7	15.4	24.3	28.7
Appenzell Ausserhoden	Global-unweighted	8.2	11.8	-15.5	-8.4	2.4	8.4	14.2	22.5	25.4
Appenzell Innerhoden	Global-unweighted	8.1	11.9	-15.3	-8.5	2.3	8.3	14.2	22.4	25.3
St. Gallen	Global-unweighted	6.9	11.9	-16.5	-9.6	1.2	7.2	13.0	21.1	24.1
Graübunden	Global-unweighted	1.8	12.8	-22.0	-15.8	-4.4	2.2	8.4	16.1	18.5
Aargau	Global-unweighted	9.7	11.5	-12.3	-5.4	3.9	9.8	15.4	24.0	27.3
Thurgau	Global-unweighted	9.6	11.9	-13.4	-5.9	3.7	9.7	15.6	24.4	27.3
Ticino	Global-unweighted	6.0	12.5	-17.0	-9.3	-0.2	6.1	12.3	19.6	22.0
Vaud	Global-unweighted	8.0	11.5	-13.6	-7.3	2.3	8.3	13.8	22.0	25.3
Valais	Global-unweighted	2.0	12.8	-21.6	-15.5	-4.2	2.4	8.6	16.5	19.6
Neuchâtel	Global-unweighted	8.9	11.2	-12.8	-6.6	3.3	9.1	14.5	22.9	26.5
Genève	Global-unweighted	9.5	11.4	-12.3	-5.3	3.7	9.5	15.2	23.5	27.4
Jura	Global-unweighted	9.3	11.3	-14.8	-6.5	3.7	9.5	15.0	23.6	27.2
overall mean		7.3	11.8	-15.6	-8.6	1.5	7.5	13.2	21.4	24.7

Table S3. Descriptive statistics of the observed temperature by exposure dataset for the 10 regions in England and Wales, 1993 – 2006

	GCD	Mean	IQR	min	1st	25th	50th	75th	99th	max
East England	Weather station	10.6	8.6	-4.15	-0.70	6.28	10.47	14.90	21.90	25.33
Yorkshire and the Humber	Weather station	9.9	8.1	-4.49	-0.34	5.92	9.76	14.02	20.94	23.19
Southeast England	Weather station	10.8	8.2	-4.12	-0.20	6.80	10.72	15.03	21.61	25.32
Eastmidlands	Weather station	10.1	8.3	-4.97	-0.79	5.93	9.95	14.26	21.34	24.02
Greater London	Weather station	11.7	8.5	-3.05	0.41	7.51	11.49	15.97	23.72	29.20
Southwest England	Weather station	10.7	7.6	-3.65	0.48	7.09	10.54	14.67	20.55	24.26
Westmidlands	Weather station	10.1	8.3	-5.18	-0.76	6.01	10.03	14.28	21.46	25.18
Northwest England	Weather station	10.2	7.8	-6.33	-0.45	6.37	10.13	14.21	21.11	25.13
Northeast England	Weather station	9.5	7.6	-5.30	-0.40	5.73	9.45	13.36	19.90	22.56
Wales	Weather station	10.4	7.5	-3.09	0.24	6.78	10.28	14.31	20.37	24.37
overall mean		10.4	8.1	-4.4	-0.3	6.4	10.3	14.5	21.3	24.9
East England	Local-weighted	10.7	8.7	-3.83	-0.73	6.32	10.53	15.03	22.04	25.83
Yorkshire and the Humber	Local-weighted	9.9	8.2	-4.73	-0.38	5.85	9.77	14.05	20.94	22.99
Southeast England	Local-weighted	10.8	8.4	-4.08	-0.10	6.75	10.72	15.12	21.74	25.39
Eastmidlands	Local-weighted	10.1	8.3	-4.92	-0.70	6.01	10.03	14.35	21.49	24.13
Greater London	Local-weighted	11.5	8.6	-2.84	0.27	7.27	11.39	15.85	23.35	28.05
Southwest England	Local-weighted	10.8	7.7	-3.60	0.35	7.11	10.70	14.83	20.82	24.44
Westmidlands	Local-weighted	10.2	8.3	-4.69	-0.82	6.07	10.05	14.34	21.61	24.89
Northwest England	Local-weighted	10.1	7.9	-5.68	-0.47	6.25	10.09	14.17	20.90	24.37
Northeast England	Local-weighted	9.5	7.7	-4.60	-0.25	5.71	9.44	13.40	19.69	21.90
Wales	Local-weighted	10.3	7.6	-3.34	0.07	6.64	10.23	14.23	20.38	24.28
overall mean		10.4	8.1	-4.2	-0.3	6.4	10.3	14.5	21.3	24.6
East England	Local-unweighted	10.2	8.4	-4.46	-0.84	6.07	10.14	14.49	21.59	24.40
Yorkshire and the Humber	Local-unweighted	9.2	7.7	-5.31	-0.91	5.47	9.10	13.15	19.72	23.24
Southeast England	Local-unweighted	10.3	7.9	-4.24	-0.40	6.45	10.17	14.38	20.78	24.34
Eastmidlands	Local-unweighted	9.6	7.9	-5.46	-1.11	5.73	9.47	13.59	20.40	23.80
Greater London	Local-unweighted	10.3	8.2	-4.32	-0.73	6.24	10.16	14.44	21.27	25.13
Southwest England	Local-unweighted	10.1	7.2	-3.66	0.10	6.66	9.98	13.84	19.71	23.66
Westmidlands	Local-unweighted	9.1	7.1	-4.35	-0.90	5.67	9.06	12.79	19.17	23.20

Northwest England	Local-unweighted	9.7	6.9	-3.46	0.79	6.41	9.59	13.31	18.53	22.47
Northeast England	Local-unweighted	8.2	7.4	-8.78	-1.73	4.56	8.17	11.96	18.31	21.55
Wales	Local-unweighted	10.5	6.7	-2.16	1.29	7.27	10.33	13.97	18.99	23.77
overall mean		9.7	7.5	-4.6	-0.4	6.1	9.6	13.6	19.8	23.6
East England	Global-weighted	10.6	8.3	-3.34	-0.37	6.62	10.55	14.88	21.54	25.07
Yorkshire and the Humber	Global-weighted	9.5	7.8	-6.64	-0.74	5.62	9.48	13.43	20.13	22.22
Southeast England	Global-weighted	10.7	8.0	-3.48	-0.39	6.89	10.66	14.92	21.70	25.16
Eastmidlands	Global-weighted	10.0	8.1	-4.63	-0.76	6.03	10.00	14.13	21.31	24.44
Greater London	Global-weighted	10.8	8.2	-3.58	-0.55	6.78	10.69	15.02	22.09	25.89
Southwest England	Global-weighted	10.7	7.4	-3.17	0.27	7.18	10.56	14.56	20.48	24.36
Westmidlands	Global-weighted	10.1	8.0	-4.13	-0.91	6.24	10.08	14.20	21.26	24.59
Northwest England	Global-weighted	9.8	7.5	-4.22	-0.48	6.15	9.85	13.65	20.06	22.82
Northeast England	Global-weighted	9.6	7.6	-7.49	-0.19	5.84	9.54	13.44	19.90	22.85
Wales	Global-weighted	10.0	7.2	-3.94	-0.15	6.58	9.94	13.80	19.84	23.58
overall mean		10.2	7.8	-4.5	-0.4	6.4	10.1	14.2	20.8	24.1
East England	Global-unweighted	10.6	8.3	-3.19	-0.25	6.52	10.48	14.82	21.47	24.63
Yorkshire and the Humber	Global-unweighted	9.4	7.7	-6.03	-0.58	5.58	9.39	13.30	19.80	21.84
Southeast England	Global-unweighted	10.7	8.0	-3.47	-0.31	6.93	10.65	14.93	21.59	24.98
Eastmidlands	Global-unweighted	10.1	8.2	-4.64	-0.66	6.07	10.07	14.23	21.27	24.29
Greater London	Global-unweighted	10.8	8.2	-3.58	-0.55	6.78	10.69	15.02	22.09	25.89
Southwest England	Global-unweighted	10.7	7.2	-3.18	0.43	7.24	10.53	14.45	20.22	24.09
Westmidlands	Global-unweighted	10.0	7.9	-4.00	-1.06	6.15	9.97	14.04	20.86	24.36
Northwest England	Global-unweighted	9.3	7.4	-5.75	-0.82	5.67	9.30	13.12	19.41	22.05
Northeast England	Global-unweighted	8.8	7.5	-8.08	-0.98	5.13	8.82	12.64	18.87	21.62
Wales	Global-unweighted	9.7	7.1	-5.05	-0.50	6.27	9.60	13.37	19.31	23.06
overall mean		10.0	7.8	-4.7	-0.5	6.2	10.0	14.0	20.5	23.7

Table S4. Relative risk (RR) for all-cause mortality (95%CI) for heat(99th) percentile) by exposure dataset for Switzerland

Canton	Weather station	Local-weighted	Local-unweighted	Global Weighted	Global Unweighted
**Zürich**	1.19 (1.11 , 1.28)	1.19 (1.11 , 1.28)	1.21 (1.12 , 1.30)	1.2 (1.12 , 1.30)	1.2 (1.12 , 1.30)
**Bern**	1.07 (1.00 , 1.16)	1.07 (0.99 , 1.15)	1.08 (1.00 , 1.16)	1.09 (1.01 , 1.17)	1.1 (1.02 , 1.18)
**Luzern**	1.14 (0.99 , 1.31)	1.15 (1.00 , 1.33)	1.16 (1.00 , 1.34)	1.15 (1.00 , 1.32)	1.14 (0.99 , 1.30)
**Uri**	1.34 (0.90 , 2.00)	1.37 (0.89 , 2.1)	1.42 (0.94 , 2.14)	1.44 (0.95 , 2.19)	1.34 (0.90 , 2.02)
**Schwyz**	1.07 (0.85 , 1.34)	1.04 (0.82 , 1.31)	1.02 (0.82 , 1.28)	1.09 (0.86 , 1.38)	1.11 (0.87 , 1.40)
**Obwalden**	1.32 (0.80 , 2.15)	1.36 (0.82 , 2.25)	1.39 (0.85 , 2.27)	1.27 (0.79 , 2.05)	1.28 (0.82 , 2.01)
**Nidwalden**	0.84 (0.53 , 1.36)	0.86 (0.53 , 1.39)	0.85 (0.53 , 1.38)	0.98 (0.61 , 1.55)	1 (0.65 , 1.54)
**Glarus**	1.27 (0.86 , 1.90)	1.25 (0.84 , 1.87)	1.26 (0.84 , 1.88)	1.27 (0.87 , 1.87)	1.28 (0.88 , 1.88)
**Zug**	0.97 (0.73 , 1.29)	0.98 (0.74 , 1.29)	0.97 (0.73 , 1.30)	1.17 (0.88 , 1.57)	1.18 (0.89 , 1.56)
**Fribourg**	1.04 (0.86 , 1.25)	1.05 (0.89 , 1.24)	1.05 (0.89 , 1.23)	1.05 (0.89 , 1.23)	1.05 (0.89 , 1.23)
**Solothurn**	0.99 (0.91 , 1.06)	1 (0.88 , 1.12)	0.99 (0.88 , 1.12)	0.99 (0.89 , 1.11)	0.99 (0.89 , 1.11)
**Basel-Stadt**	1.25 (1.09 , 1.45)	1.25 (1.08 , 1.45)	1.25 (1.08 , 1.45)	1.21 (1.06 , 1.39)	1.21 (1.06 , 1.39)
**Basel-Landschaft**	1.2 (1.03 , 1.40)	1.19 (1.03 , 1.39)	1.19 (1.02 , 1.39)	1.19 (1.02 , 1.39)	1.19 (1.02 , 1.39)
**Schaffhausen**	1.1 (0.84 , 1.43)	1.09 (0.84 , 1.42)	1.08 (0.83 , 1.42)	1.11 (0.85 , 1.45)	1.1 (0.84 , 1.44)
**Appenzell Ausserhoden**	1.27 (0.87 , 1.85)	1.28 (0.88 , 1.86)	1.28 (0.89 , 1.85)	1.31 (0.89 , 1.94)	1.31 (0.89 , 1.94)
**Appenzell Innerhoden**	0.9 (0.44 , 1.83)	0.91 (0.46 , 1.78)	0.91 (0.46 , 1.80)	0.85 (0.42 , 1.74)	0.87 (0.43 , 1.75)
**St. Gallen**	1.16 (1.02 , 1.33)	1.17 (1.03 , 1.34)	1.18 (1.03 , 1.35)	1.08 (0.94 , 1.23)	1.07 (0.94 , 1.22)
**Graübunden**	0.93 (0.76 , 1.12)	0.93 (0.77 , 1.12)	0.94 (0.78 , 1.12)	0.94 (0.79 , 1.12)	0.94 (0.79 , 1.11)
**Aargau**	1.16 (1.05 , 1.28)	1.15 (1.04 , 1.28)	1.16 (1.04 , 1.28)	1.18 (1.05 , 1.32)	1.18 (1.05 , 1.32)
**Thurgau**	0.99 (0.84 , 1.18)	0.99 (0.84 , 1.17)	0.99 (0.84 , 1.17)	1.08 (0.90 , 1.29)	1.08 (0.90 , 1.29)
**Ticino**	1.18 (1.06 , 1.33)	1.21 (1.07 , 1.36)	1.23 (1.09 , 1.40)	1.21 (1.08 , 1.37)	1.25 (1.10 , 1.41)
**Vaud**	1.09 (0.99 , 1.21)	1.1 (0.99 , 1.22)	1.1 (0.99 , 1.22)	1.13 (1.01 , 1.26)	1.14 (1.02 , 1.27)
**Valais**	1.33 (1.14 , 1.54)	1.3 (1.13 , 1.51)	1.28 (1.11 , 1.49)	1.24 (1.08 , 1.43)	1.22 (1.06 , 1.41)
**Neuchâtel**	1.13 (0.96 , 1.33)	1.18 (0.99 , 1.42)	1.19 (1.00 , 1.43)	1.12 (0.94 , 1.33)	1.13 (0.95 , 1.35)
**Genève**	1.15 (1.04 , 1.29)	1.16 (1.03 , 1.29)	1.15 (1.03 , 1.28)	1.16 (1.04 , 1.30)	1.16 (1.04 , 1.30)
**Jura**	1.01 (0.77 , 1.32)	1.02 (0.77 , 1.34)	1.02 (0.77 , 1.35)	1.01 (0.74 , 1.37)	1.01 (0.76 , 1.36)

Table S5. Relative risk (RR) for all-cause mortality (95%CI) for cold (1st percentile) by exposure dataset for Switzerland

Canton	Weather station	Local-weighted	Local-unweighted	Global Weighted	Global Unweighted
**Zürich**	1.19 (1.10 , 1.28)	1.19 (1.10 , 1.28)	1.18 (1.09 , 1.28)	1.17 (1.08 , 1.26)	1.17 (1.09 , 1.26)
**Bern**	1.27 (1.17 , 1.39)	1.24 (1.14 , 1.35)	1.2 (1.11 , 1.31)	1.24 (1.14 , 1.35)	1.24 (1.14 , 1.36)
**Luzern**	1.3 (1.10 , 1.54)	1.29 (1.10 , 1.51)	1.28 (1.09 , 1.50)	1.26 (1.07 , 1.48)	1.26 (1.07 , 1.48)
**Uri**	0.93 (0.57 , 1.52)	1.12 (0.73 , 1.72)	1.18 (0.77 , 1.81)	1.09 (0.66 , 1.81)	1.09 (0.65 , 1.82)
**Schwyz**	1.46 (1.08 , 1.97)	1.47 (1.10 , 1.95)	1.4 (1.05 , 1.86)	1.46 (1.09 , 1.96)	1.45 (1.08 , 1.95)
**Obwalden**	1.6 (1.01 , 2.52)	1.48 (0.95 , 2.30)	1.47 (0.95 , 2.29)	1.36 (0.8 , 2.31)	1.4 (0.83 , 2.36)
**Nidwalden**	1.3 (0.82 , 2.06)	1.32 (0.83 , 2.12)	1.27 (0.79 , 2.04)	1.42 (0.85 , 2.39)	1.46 (0.86 , 2.47)
**Glarus**	1.23 (0.82 , 1.85)	1.13 (0.78 , 1.63)	1.13 (0.78 , 1.63)	1.23 (0.81 , 1.87)	1.25 (0.81 , 1.93)
**Zug**	1.3 (0.91 , 1.86)	1.3 (0.92 , 1.83)	1.28 (0.91 , 1.81)	1.25 (0.88 , 1.76)	1.23 (0.87 , 1.75)
**Fribourg**	1.13 (0.92 , 1.39)	1.11 (0.93 , 1.33)	1.12 (0.94 , 1.34)	1.11 (0.92 , 1.35)	1.11 (0.91 , 1.34)
**Solothurn**	1.24 (1.01 , 1.51)	1.22 (1.01 , 1.49)	1.21 (1.00 , 1.46)	1.18 (0.97 , 1.42)	1.17 (0.97 , 1.42)
**Basel-Stadt**	1.28 (1.08 , 1.51)	1.28 (1.08 , 1.52)	1.28 (1.08 , 1.52)	1.33 (1.13 , 1.56)	1.32 (1.13 , 1.56)
**Basel-Landschaft**	1.37 (1.15 , 1.64)	1.37 (1.14 , 1.65)	1.35 (1.13 , 1.62)	1.33 (1.10 , 1.60)	1.33 (1.10 , 1.60)
**Schaffhausen**	1.22 (0.91 , 1.65)	1.21 (0.91 , 1.62)	1.21 (0.90 , 1.61)	1.13 (0.88 , 1.45)	1.11 (0.86 , 1.43)
**Appenzell Ausserhoden**	0.88 (0.69 , 1.13)	0.89 (0.70 , 1.14)	0.89 (0.69 , 1.15)	0.99 (0.76 , 1.29)	0.99 (0.76 , 1.29)
**Appenzell Innerhoden**	1.25 (0.63 , 2.48)	1.24 (0.64 , 2.40)	1.24 (0.65 , 2.36)	1.27 (0.59 , 2.73)	1.28 (0.60 , 2.74)
**St. Gallen**	1.24 (1.08 , 1.42)	1.25 (1.08 , 1.43)	1.24 (1.08 , 1.42)	1.25 (1.08 , 1.44)	1.24 (1.07 , 1.44)
**Graübunden**	1.09 (0.92 , 1.27)	1.07 (0.91 , 1.24)	1.07 (0.91 , 1.25)	1.17 (0.94 , 1.45)	1.17 (0.94 , 1.46)
**Aargau**	1.16 (1.02 , 1.33)	1.13 (1.00 ,1.29)	1.13 (1.00 , 1.28)	1.15 (1.02 , 1.29)	1.14 (1.02 , 1.28)
**Thurgau**	1.3 (1.07 , 1.58)	1.31 (1.08 , 1.58)	1.31 (1.08 , 1.58)	1.29 (1.05 , 1.57)	1.29 (1.05 , 1.57)
**Ticino**	1.24 (1.02 , 1.51)	1.26 (1.05 , 1.51)	1.25 (1.06 , 1.46)	1.24 (1.03 , 1.49)	1.28 (1.06 , 1.53)
**Vaud**	1.09 (1.00 , 1.19)	1.09 (1.00 , 1.18)	1.09 (1.00 , 1.18)	1.11 (1.02 , 1.21)	1.11 (1.02 , 1.21)
**Valais**	1.11 (0.91 , 1.35)	1.1 (0.93 , 1.29)	1.06 (0.90 , 1.26)	1.23 (1.02 , 1.49)	1.19 (0.98 , 1.44)
**Neuchâtel**	1.31 (1.06 , 1.63)	1.29 (1.05 , 1.60)	1.26 (1.02 , 1.56)	1.34 (1.09 , 1.65)	1.34 (1.08 , 1.66)
**Genève**	1.22 (1.04 , 1.43)	1.21 (1.03 , 1.41)	1.21 (1.03 , 1.41)	1.22 (1.05 , 1.41)	1.22 (1.05 , 1.42)
**Jura**	1.05 (0.80 , 1.39)	1.07 (0.82 , 1.40)	1.08 (0.82 , 1.42)	1.01 (0.80 , 1.28)	1.01 (0.79 , 1.30)

Table S6. Relative risk (RR) for all-cause mortality (95%CI) for heat(99th percentile) by exposure dataset for England and Wales

Region	Weather station	Local-weighted	Local-unweighted	Global-weighted	Global-unweighted
**East**	1.13 (1.08 , 1.18)	1.13 (1.08 , 1.18)	1.13 (1.07 , 1.19)	1.16 (1.11 , 1.22)	1.16 (1.11 , 1.22)
**York**	1.07 (1.01 , 1.13)	1.07 (1.01 , 1.13)	1.08 (1.02 , 1.14)	1.08 (1.02 , 1.15)	1.08 (1.02 , 1.15)
**Stheast**	1.11 (1.07 , 1.15)	1.11 (1.07 , 1.15)	1.1 (1.05 , 1.15)	1.13 (1.08 , 1.17)	1.13 (1.08 , 1.17)
**Eastmid**	1.09 (1.03 , 1.15)	1.09 (1.02 , 1.15)	1.08 (1.01 , 1.15)	1.1 (1.04 , 1.17)	1.11 (1.04 , 1.18)
**London**	1.25 (1.19 , 1.30)	1.24 (1.18 , 1.29)	1.16 (1.10 , 1.22)	1.26 (1.20 , 1.33)	1.26 (1.20 , 1.33)
**Sthwest**	1.09 (1.03 , 1.15)	1.08 (1.03 , 1.14)	1.09 (1.04 , 1.15)	1.09 (1.04 , 1.15)	1.09 (1.04 , 1.15)
**Westmid**	1.08 (1.02 , 1.14)	1.09 (1.03 , 1.15)	1.05 (0.99 , 1.11)	1.07 (1.02 , 1.13)	1.06 (1.01 , 1.12)
**Northwest**	1.11 (1.05 , 1.16)	1.1 (1.05 , 1.16)	1.07 (1.02 , 1.11)	1.11 (1.06 , 1.17)	1.1 (1.05 , 1.15)
**Northeast**	1.11 (1.04 , 1.20)	1.11 (1.03 , 1.19)	1.12 (1.05 , 1.20)	1.13 (1.05 , 1.21)	1.13 (1.05 , 1.21)
**Wales**	1.08 (1.01 , 1.15)	1.08 (1.01 , 1.15)	1.05 (0.99 , 1.12)	1.08 (1.01 , 1.15)	1.08 (1.02 , 1.15)

Table S7. Relative risk (RR) for all-cause mortality (95%CI) for cold (1st percentile) by exposure dataset for England & Wales

Region	Weather station	Local-weighted	Local-unweighted	Global-weighted	Global-unweighted
**East**	1.45 (1.37 , 1.55)	1.47 (1.38 , 1.56)	1.39 (1.31 , 1.48)	1.45 (1.36 , 1.55)	1.44 (1.36 , 1.54)
**York**	1.27 (1.20 , 1.35)	1.27 (1.19 , 1.34)	1.25 (1.17 , 1.32)	1.25 (1.18 , 1.32)	1.24 (1.18 , 1.32)
**Stheast**	1.45 (1.38 , 1.52)	1.44 (1.37 , 1.51)	1.42 (1.35 , 1.49)	1.45 (1.38 , 1.52)	1.45 (1.38 , 1.52)
**Eastmid**	1.35 (1.26 , 1.45)	1.34 (1.26 , 1.44)	1.31 (1.23 , 1.40)	1.31 (1.23 , 1.39)	1.32 (1.24 , 1.41)
**London**	1.46 (1.38 , 1.55)	1.45 (1.37 , 1.54)	1.41 (1.33 , 1.49)	1.44 (1.36 , 1.53)	1.44 (1.35 , 1.53)
**Sthwest**	1.47 (1.39 , 1.55)	1.49 (1.41 , 1.58)	1.46 (1.38 , 1.54)	1.49 (1.41 , 1.58)	1.48 (1.40 , 1.56)
**Westmid**	1.36 (1.28 , 1.44)	1.37 (1.29 , 1.46)	1.31 (1.23 , 1.39)	1.35 (1.27 , 1.43)	1.36 (1.28 , 1.44)
**Northwest**	1.3 (1.23 , 1.36)	1.31 (1.24 , 1.38)	1.27 (1.21 , 1.34)	1.27 (1.21 , 1.34)	1.25 (1.19 , 1.31)
**Northeast**	1.28 (1.19 , 1.38)	1.27 (1.18 , 1.37)	1.24 (1.16 , 1.33)	1.23 (1.15 , 1.32)	1.22 (1.14 , 1.30)
**Wales**	1.42 (1.32 , 1.53)	1.4 (1.31 , 1.50)	1.38 (1.29 , 1.48)	1.39 (1.29 , 1.49)	1.37 (1.28 , 1.47)

Table S8. Goodness of fit for each exposure dataset measured by the qAIC (quasi-Akaike's information criterion) averaged over the 10 regions in England and Wales

	Weather station	Local weighted	Local unweighted	Global weighted	Global unweighted
East England	40666.38	40654.22	40672.47	40640.14	40650.56
Yorkshire and the Humber	40738.83	40742.58	40766.43	40775.96	40782.92
South East England	43079.94	43071.49	43159.07	43133.06	43131.59
Eastmidlands	39675.26	39680.61	39735.33	39736.1	39734.49
Greater London	41196.32	41231.03	41412.47	41317.58	41317.59
Southwest England	40738.45	40745.02	40795.72	40784.0	40790.84
Westmidlands	40788.04	40787.33	40918.53	40823.9	40826.85
Northwest England	42875.75	42853.42	42921.26	42896.58	42920.16
Northeast England	37095.45	37106.52	37112.38	37119.98	37118.58
Wales	37857.14	37858.77	37864.01	37865.74	37873.73

Table S9. Goodness of fit for each exposure dataset measured by the qAIC (quasi-Akaike's information criterion) averaged over the 26 regions in Switzerland

	Weather station	Local-weighted	Local-unweighted	Global weighted	Global unweighted
Zürich	66042.0	66042.9	66052.7	66043.3	66043.3
Bern	64497.7	64499.5	64503.6	64509.0	64513.0
Luzern	51695.1	51698.2	51702.4	51699.0	51698.9
Uri	25960.8	25965.5	25964.0	25962.6	25965.5
Schwyz	39946.4	39946.6	39946.3	39945.1	39944.3
Obwalden	23840.5	23840.5	23840.5	23847.3	23846.3
Nidwalden	24133.3	24135.4	24136.8	24141.6	24143.4
Glarus	28561.6	28555.8	28555.8	28560.2	28561.0
Zug	35024.4	35024.8	35025.8	35031.5	35032.9
Fribourg	47351.1	47350.6	47350.8	47356.0	47356.0
Solothurn	49130.9	49130.5	49130.5	49141.2	49141.1
Basel-Stadt	49484.3	49484.3	49484.4	49473.8	49473.9
Basel-Landschaft	48436.3	48436.3	48437.5	48446.8	48446.6
Schaffhausen	36345.4	36346.2	36346.4	36350.3	36350.1
Appenzell Ausserhoden	31983.3	31983.4	31982.9	31973.5	31974.2
Appenzell Innerhoden	16933.9	16927.9	16925.1	16933.1	16933.4
St. Gallen	54716.0	54716.5	54718.9	54719.2	54719.4
Graübunden	45517.1	45525.1	45526.3	45520.8	45521.6
Aargau	55940.5	55939.6	55939.4	55953.8	55954.4
Thurgau	46974.1	46966.5	46966.7	46976.3	46976.0
Ticino	51926.3	51922.7	51924.5	51929.3	51930.3
Vaud	58818.2	58813.5	58810.1	58828.1	58826.8
Valais	49283.2	49273.5	49273.0	49279.3	49281.9
Neuchâtel	45559.3	45552.6	45549.9	45554.3	45554.2
Genève	52983.4	52984.9	52984.3	52979.1	52978.4
Jura	34875.1	34876.4	34876.6	34883.1	34882.4

Table S10. Annual excess number of deaths and mortality fractions (%) for cold (≤10th , ≤25th percentile) and heat (≥75th and ≥90th percentile) in Switzerland between 1989 and 2017 for each exposure dataset

	**Switzerland**				
**GCD**	**≤10th**	**≤25th**	**≥75th**	**≥90th**	**Total**
**Weather station**	964 (797 - 1136)	1,991 (1,557 – 2,365)	404 (245 - 547)	280 (191 - 368)	4,200 (3168 - 5142)
	1.53% (1.26 – 1.81)	3.17% (2.48 - 3.76)	0.64% (0.39 - 0.87)	0.45% (0.30 – 0.59)	6.68% (5.04 - 8.18)
**Local weighted**	931 (754 – 1,086)	1,905 (1,509 - 2,293)	409 (249 - 560)	278 (185 - 365)	4,038 (3,001 – 4,977)
	1.48% (1.20 – 1.73)	3.03% (2.40 - 3.3.65)	0.65% (0.40 - 0.89)	0.44% (0.29 - 0.58)	6.43% (4.77 - 7.92)
**Local unweighted**	888 (722 – 1,058)	1,815 (1,440 - 2,176)	423 (261 - 565)	287 (193 - 382)	3,782 (2,870 - 4,755)
	1.41 % (1.15 – 1.68)	2.89% (2.29 - 3.46)	0.67% (0.42 - 0.90)	0.46% (0.31 - 0.61)	6.02% (4.57 - 7.57)
**Global weighted**	932 (756 – 1,106)	1,853 (1,448 - 2,243)	439 (270 - 596)	311 (217 - 403)	3,953 (2,964 - 4,919)
	1.48% (1.20 – 1.76)	2.97% (2.34 - 3.65)	0.70% (0.43 - 0.95)	0.49% (0.35 - 0.64)	6.29% (4.72 - 7.83)
**Global unweighted**	925 (747 – 1,088)	1,871 (1,444 - 2,255)	437 (268- 603)	311 (216 - 408)	3,928 (2,884 - 4,947)
	1.47% (1.19 – 1.73)	2.97% (2.30 - 3.59)	0.69% (0.43 - 0.96)	0.49 (0.34 - 0.65)	6.25% (4.59 – 7.87)

Table S11. Annual excess number of deaths and mortality fractions (%) for cold (≤10th , ≤25th percentile) and heat (≥75th and ≥90th percentile) in England & Wales between 1993 and 2006 for each exposure dataset

	**England & Wales**				
**GCD**	**≤10th**	**≤25th**	**≥75th**	**≥90th**	**total**
**Weather station**	11,654 (10,788 – 12,393)	23,036 (21,091 – 25,015)	2,979 (2,419 - 3,493)	1,776 (1,450 - 2,111)	49,277(45,094 – 54,721))
	2.15% (1.99 – 2.29)	4.26% (3.90 - 4.62)	0.55% (0.45 - 0.65)	0.33 (0.27 – 0.38)	9.11% (8.33 - 10.03)
**Local weighted**	11,714 (10,942 - 12,477)	23,204 (21,216 - 25,093)	2,943 (2,404 - 3,465)	1,765 (1,450 – 2,060)	49775 (44,552 - 54,289)
	2.16% (2.02-2.31)	4.29 (3.92 - 4.64)	0.54% (0.44 - 0.64)	0.33% (0.27-0.38)	9.20% (8.24 - 10.03)
**Local unweighted**	10,958 (10,104 – 11,755)	21,033 (19,092 - 22,799)	2,064 (1,550 - 2,559)	1,483 (1,092 – 1,823)	41,962 (37,235 - 46,990)
	2.03% (1.87 – 2.17)	3.88% (3.55 - 4.24)	0.38% (0.29 - 0.47)	0.27% (0.20 – 0.34)	7.76% (6.88 - 8.69)
**Global weighted**	10,764 (10,009 – 11,531)	20,941 (19,008 - 22,822)	2,906 (2,425 - 3,401)	1,952 (1,581 – 2,292)	46,008(41,180 - 50,871)
	1.99% (1.85 – 2.13)	3.87% (3.51 - 4.22)	0.53% (0.45 - 0.63)	0.36% (0.29 - 0.42)	8.50% (7.61 - 9.40)
**Global unweighted**	10,750 (10,010 – 11,509)	20,899 (19,013 - 22,770)	2,842 (2,349 - 3,350)	1,892 (1,566 – 2,251)	45,485 (40,871 - 50,459)
	1.99% (1.85 – 2.13)	3.86% (3.51 - 4.21)	0.53% (0.43 - 0.62)	0.35% (0.29 – 0.42)	8.41% (7.55 - 9.33)

Table S12. Annual number of deaths and mortality fractions (%) for cold, ≤10th , ≤25th percentile, ≥75th and ≥90th percentile for the four selected regions for each exposure dataset

	**Zürich**			
**GCD**	**≤10th**	**≤25th**	**≥75th**	**≥90th**
**Weather station**	155 (89 , 220)	305 (139 , 458)	74 (16 , 129)	71 (22 , 113)
	1.47 (0.84 , 2.08)	2.89% (1.31, 4.33)	0.70% (0.15 , 1.22)	0.67% (0.21 , 1.07)
**Local weighted**	156 (90 , 223)	311 (152 , 465)	78 (22 , 130)	70 (25 , 112)
	1.48% (0.85 , 2.11)	2.94% (1.44 , 4.39)	0.74% (0.21 , 1.23)	0.66% (0.24 , 1.06)
**Local unweighted**	150 (81 , 212)	298 (136 , 445)	81 (25 , 135)	77 (29 , 123)
	1.41% (0.77 , 2.00)	2.81% (1.29 , 4.21)	0.77% (0.23 , 1.28)	0.73% (0.27 , 1.17)
**Global weighted**	141 (69 , 209)	276 (108 ,427)	88 (24 , 144)	81 (32 , 126)
	1.34% (0.65 , 1.97)	2.61% (1.03 , 4.03)	0.83% (0.22 , 1.36)	0.77% (0.30 , 1.19)
**Global unweighted**	144 (72 , 208)	283 (112 , 437)	88 (24 , 148)	79 (35 , 126)
	1.36% (0.68 , 1.97)	2.67% (1.06 , 4.13)	0.83% (0.23 , 1.40)	0.75% (0.33 , 1.19)
	**Ticino**			
	**≤10th**	**≤25th**	**≥75th**	**≥90th**
**Weather station**	46 (-12 , 88)	86 (-49 , 191)	18 (-2 , 36)	14 (5 , 24)
	1.62% (-0.43 , 3.10)	3.03% (-1.74 , 6.76)	0.62% (-0.06 , 1.27)	0.51% (0.18 , 0.83)
**Local weighted**	46 (-6 , 88)	89 (-20 , 188)	19 (3 , 33)	15 (6 , 26)
	1.61% (-0.21 , 3.13)	3.14 (-0.70 , 6.63)	0.66% (0.12 , 1.15)	0.54% (0.2 , 0.92)
**Local unweighted**	46 (6 , 80)	87 (-10 , 176)	19 (7 , 29)	16 (3 , 29)
	1.64% (0.22 , 2.82)	3.09% (-0.37 , 6.21)	0.66% (0.25 , 1.03)	0.57% (0.11 , 1.01)
**Global weighted**	46 (-2 , 92)	82 (-41 , 193)	18 (3 , 33)	18 (5 , 29)
	1.63% (-0.07 , 3.27)	2.89% (-1.43 , 6.81)	0.65% (0.11 , 1.15)	0.62 (0.19 , 1.01)
**Global unweighted**	50 (5 , 94)	99 (-18 , 197)	23 (1 , 43)	19 (9 , 29)
	1.75% (0.17 , 3.31)	3.49% (-0.65 , 6.97)	0.80 % (0.04 , 1.54)	0.69 (0.32 , 1.02)

	London			
GCD	≤ 10th	≤ 25th	≥75th	≥90th
Weather station	1,602 (1,322 , 1,851)	3,200 (2,566 , 3,816)	760 (488 , 1,003)	338 (280 , 396)
	2.65% (2.19 , 3.06)	5.30% (4.25-6.32)	1.23% (0.81 , 1.66)	0.56% (0.46 , 0.66)
Local weighted	1,600 (1,358 , 1,863)	3,199 (2,544 , 3,836)	716 (505 , 930)	309 (250 , 363)
	2.65 (2.25 , 3.08)	5.30% (4.21 , 6.35)	1.19% (0.84 , 1.54)	0.51% (0.41 , 0.60)
Local unweighted	1,454 (1176 , 1706)	2,799 (2,161 , 3,447)	394 (203 , 572)	230 (148 , 303)
	2.40 (1.94 , 2.80)	4.64% (3.58 , 5.71)	0.66% (0.34 , 0.95)	0.38% (0.25 , 0.51)
Global weighted	1,531 (1,244 , 1,780)	3,033 (2,336 , 3,679)	756 (502 , 993)	344 (279 , 407)
	2.54 (2.06 , 2.95)	5.02% (4.05 , 6.08)	1.25% (0.83 , 1.65)	0.57% (0.46 , 0.67)
Global unweighted	1,531 (1,245 , 1,771)	3,048 (2,307 , 3,651)	769 (523 , 989)	342 (277 , 402)
	2.54 (2.06 , 2.93)	5.05% (3.82 , 6.05)	1.27% (0.87 , 1.64)	0.35% (0.46 , 0.66)
	Northeast England			
	≤10th	≤25th	≥75th	≥90th
Weather station	435 (256 , 601)	856 (421 , 1291)	132 (44 , 221)	109 (10 , 202)
	1.49% (0.88 , 2.06)	2.94% (1.46 , 4.43)	0.45% (0.15 , 0.76)	0.37% (0.03 , 0.69)
Local weighted	445 (266 , 610)	892 (390 , 1302)	119 (35 , 199)	98 (6 , 193)
	1.53% (0.91 , 2.09)	3.06% (1.34 , 4.47)	0.41% (0.12 , 0.68)	0.34% (0.02 , 0.66)
Local unweighted	458 (289 , 631)	902 (453 , 1311)	116 (52 , 172)	92 (19 , 165)
	1.59% (0.97 , 2.17)	3.10% (1.56 , 4.50)	0.40% (0.18 , 0.59)	0.32% (0.06 , 0.57)
Global weighted	424 (233 , 589)	852 (405 , 1,254)	127 (59 , 186)	111 (35 , 175)
	1.46% (0.80 , 2.02)	2.92% (2.89 , 4.31)	0.44% (0.41 , 0.64)	0.38% (0.12 , 0.60)
Global unweighted	414 (236 , 583)	843 (424 , 1264)	119 (56 , 181)	100 (24 , 175)
	1.42% (0.81 , 2.00)	2.89% (1.46 , 4.34)	0.41% (0.19 , 0.62)	0.34% (0.08 , 0.60)

Table S13. Summary statistics for the annual excess number of deaths, mortality fractions and associated 95% CI for heat (≤ 90th percentile and ≤ 75th percentile) in Switzerland

		≥ 90th percentile		≥ 75th percentile	
**Canton**	**GCD**	**AN**	**AF**	**AN**	**AF**
Aargau	Weather station	20 (7;32)	0.48% (0.16;0.76)	20 (-14;54)	0.49% (-0.34;1.28)
Aargau	Local-weighted	18 (-3;37)	0.43% (-0.06;0.88)	18 (1;33)	0.43% (0.02;0.79)
Aargau	Local-unweighted	17 (-4;39)	0.42% (-0.08;0.93)	19 (1;35)	0.45% (0.02;0.84)
Aargau	Global-weighted	24 (-4;53)	0.58% (-0.1;1.27)	28 (-12;68)	0.68% (-0.28;1.63)
Aargau	Global-unweighted	26 (-6;54)	0.62% (-0.14;1.29)	28 (-11;66)	0.68% (-0.27;1.58)
Appenzell.Ausserhoden	Weather station	6 (-6;15)	1.08% (-1.16;2.99)	7 (-18;29)	1.39% (-3.44;5.63)
Appenzell.Ausserhoden	Local-weighted	6 (-6;15)	1.14% (-1.21;2.99)	8 (-18;28)	1.56% (-3.54;5.43)
Appenzell.Ausserhoden	Local-unweighted	6 (-4;15)	1.13% (-0.79;2.9)	9 (-17;28)	1.74% (-3.38;5.34)
Appenzell.Ausserhoden	Global-weighted	8 (-5;17)	1.48% (-0.94;3.37)	10 (-18;32)	1.91% (-3.44;6.29)
Appenzell.Ausserhoden	Global-unweighted	8 (-5;17)	1.5% (-0.97;3.26)	12 (-19;34)	2.24% (-3.62;6.65)
Appenzell.Innerhoden	Weather station	-1 (-6;2)	-0.41% (-4.55;1.26)	0 (-10;5)	-0.23% (-7.54;3.84)
Appenzell.Innerhoden	Local-weighted	-1 (-6;2)	-0.5% (-4.03;1.54)	0 (-7;4)	0.12% (-4.75;2.91)
Appenzell.Innerhoden	Local-unweighted	-1 (-6;2)	-0.47% (-4.46;1.51)	0 (-7;4)	0.04% (-4.83;2.87)
Appenzell.Innerhoden	Global-weighted	-1 (-6;2)	-0.62% (-4.39;1.14)	0 (-11;6)	0.1% (-7.82;4.35)
Appenzell.Innerhoden	Global-unweighted	-1 (-6;2)	-0.8% (-4.69;1.16)	0 (-8;5)	0.33% (-6.03;3.42)
Basel.Landschaft	Weather station	10 (1;18)	0.49% (0.04;0.84)	21 (-3;42)	0.99% (-0.14;2)
Basel.Landschaft	Local-weighted	11 (2;18)	0.51% (0.09;0.87)	20 (-14;49)	0.95% (-0.66;2.33)
Basel.Landschaft	Local-unweighted	10 (2;18)	0.5% (0.09;0.85)	21 (-12;51)	0.98% (-0.59;2.45)
Basel.Landschaft	Global-weighted	10 (1;17)	0.48% (0.05;0.84)	24 (-9;53)	1.13% (-0.44;2.54)
Basel.Landschaft	Global-unweighted	10 (2;18)	0.49% (0.09;0.85)	24 (-10;55)	1.14% (-0.49;2.63)
Basel.Stadt	Weather station	16 (6;24)	0.67% (0.26;1.02)	22 (-2;43)	0.95% (-0.09;1.87)
Basel.Stadt	Local-weighted	15 (5;23)	0.65% (0.23;0.99)	21 (-3;44)	0.9% (-0.12;1.91)
Basel.Stadt	Local-unweighted	15 (5;23)	0.65% (0.22;1)	20 (-3;41)	0.84% (-0.12;1.76)
Basel.Stadt	Global-weighted	15 (2;28)	0.65% (0.07;1.22)	17 (6;27)	0.73% (0.27;1.16)
Basel.Stadt	Global-unweighted	15 (0;28)	0.65% (0.02;1.22)	17 (6;27)	0.74% (0.26;1.16)
Bern	Weather station	36 (-6;77)	0.39% (-0.06;0.83)	41 (-12;89)	0.44% (-0.13;0.96)
Bern	Local-weighted	36 (-10;77)	0.39% (-0.11;0.83)	40 (-15;95)	0.43% (-0.16;1.02)
Bern	Local-unweighted	35 (-9;75)	0.38% (-0.09;0.82)	38 (-21;90)	0.41% (-0.23;0.98)
Bern	Global-weighted	42 (-4;87)	0.45% (-0.04;0.95)	50 (-4;101)	0.54% (-0.05;1.09)

Bern	Global-unweighted	44 (1;84)	0.48% (0.01;0.92)	48 (-3;98)	0.52% (-0.03;1.07)
Fribourg	Weather station	4 (-16;22)	0.2% (-0.82;1.15)	5 (-15;23)	0.27% (-0.78;1.23)
Fribourg	Local-weighted	5 (-11;19)	0.24% (-0.57;1.02)	5 (-8;18)	0.29% (-0.44;0.93)
Fribourg	Local-unweighted	5 (-12;21)	0.25% (-0.65;1.09)	7 (-10;21)	0.36% (-0.53;1.09)
Fribourg	Global-weighted	4 (-13;19)	0.23% (-0.7;1.02)	6 (-11;20)	0.3% (-0.58;1.07)
Fribourg	Global-unweighted	4 (-16;20)	0.22% (-0.86;1.05)	5 (-14;22)	0.26% (-0.76;1.15)
Genève	Weather station	14 (2;26)	0.46% (0.06;0.81)	21 (1;40)	0.67% (0.03;1.26)
Genève	Local-weighted	13 (1;25)	0.42% (0.03;0.78)	22 (2;40)	0.69% (0.06;1.27)
Genève	Local-unweighted	14 (2;25)	0.43% (0.06;0.78)	22 (0;40)	0.7% (0.01;1.27)
Genève	Global-weighted	15 (3;27)	0.47% (0.11;0.84)	19 (-3;39)	0.59% (-0.1;1.23)
Genève	Global-unweighted	15 (3;27)	0.46% (0.09;0.86)	20 (-2;39)	0.63% (-0.06;1.25)
Glarus	Weather station	5 (-5;12)	1.35% (-1.15;3.18)	6 (-7;15)	1.48% (-1.75;3.93)
Glarus	Local-weighted	6 (-4;12)	1.41% (-0.94;3.07)	7 (-5;16)	1.74% (-1.18;3.98)
Glarus	Local-unweighted	5 (-4;12)	1.36% (-0.94;3.11)	6 (-5;15)	1.59% (-1.39;3.9)
Glarus	Global-weighted	7 (-2;13)	1.68% (-0.62;3.32)	7 (-4;17)	1.91% (-1.02;4.22)
Glarus	Global-unweighted	6 (-3;13)	1.62% (-0.66;3.24)	7 (-2;15)	1.85% (-0.4;3.75)
Graübunden	Weather station	0 (-21;17)	0.01% (-1.31;1.05)	2 (-30;30)	0.12% (-1.86;1.84)
Graübunden	Local-weighted	2 (-18;19)	0.12% (-1.07;1.15)	8 (-28;38)	0.48% (-1.7;2.31)
Graübunden	Local-unweighted	4 (-17;24)	0.22% (-1.01;1.47)	10 (-28;41)	0.62% (-1.71;2.53)
Graübunden	Global-weighted	4 (-17;21)	0.25% (-1.02;1.28)	7 (-17;29)	0.43% (-1.06;1.78)
Graübunden	Global-unweighted	5 (-14;23)	0.31% (-0.83;1.39)	8 (-16;28)	0.51% (-1;1.73)
Jura	Weather station	2 (-12;13)	0.31% (-1.8;1.92)	3 (-14;17)	0.41% (-2.08;2.57)
Jura	Local-weighted	2 (-10;12)	0.38% (-1.58;1.88)	2 (-13;16)	0.38% (-2.02;2.52)
Jura	Local-unweighted	2 (-10;12)	0.34% (-1.55;1.86)	3 (-13;16)	0.45% (-1.97;2.51)
Jura	Global-weighted	0 (-15;13)	0.07% (-2.23;1.94)	1 (-26;27)	0.22% (-3.99;4.11)
Jura	Global-unweighted	1 (-13;13)	0.16% (-2.05;1.93)	2 (-19;20)	0.24% (-2.94;3.13)
Luzern	Weather station	10 (-1;20)	0.36% (-0.02;0.69)	22 (-17;55)	0.78% (-0.59;1.95)
Luzern	Local-weighted	11 (0;20)	0.38% (0.01;0.71)	24 (-10;54)	0.85% (-0.35;1.93)
Luzern	Local-unweighted	12 (0;21)	0.41% (0;0.73)	26 (-14;60)	0.93% (-0.49;2.14)

Luzern	Global-weighted	12 (0;22)	0.41% (0.01;0.77)	18 (-12;46)	0.65% (-0.42;1.61)
Luzern	Global-unweighted	11 (0;21)	0.4% (0.01;0.75)	17 (-16;44)	0.59% (-0.56;1.57)
Neuchâtel	Weather station	6 (-2;13)	0.37% (-0.1;0.81)	9 (-10;27)	0.53% (-0.64;1.7)
Neuchâtel	Local-weighted	8 (-1;14)	0.47% (-0.03;0.89)	12 (-13;36)	0.76% (-0.83;2.23)
Neuchâtel	Local-unweighted	8 (0;15)	0.5% (-0.02;0.95)	13 (-7;32)	0.82% (-0.43;1.99)
Neuchâtel	Global-weighted	5 (-5;15)	0.32% (-0.34;0.93)	6 (-6;16)	0.4% (-0.34;1.01)
Neuchâtel	Global-unweighted	6 (-3;13)	0.36% (-0.17;0.82)	9 (-15;29)	0.54% (-0.93;1.81)
Nidwalden	Weather station	0 (-11;7)	0.08% (-4.14;2.65)	1 (-10;9)	0.25% (-3.88;3.41)
Nidwalden	Local-weighted	1 (-9;8)	0.35% (-3.43;2.94)	2 (-9;10)	0.72% (-3.37;3.7)
Nidwalden	Local-unweighted	1 (-9;8)	0.4% (-3.33;2.89)	2 (-8;11)	0.83% (-3.15;3.96)
Nidwalden	Global-weighted	2 (-6;9)	0.86% (-2.32;3.21)	3 (-7;11)	1.25% (-2.66;4.02)
Nidwalden	Global-unweighted	2 (-6;8)	0.89% (-2.13;3.03)	3 (-7;11)	1.22% (-2.48;3.98)
Obwalden	Weather station	6 (0;12)	2.45% (-0.13;4.48)	8 (-1;15)	3.12% (-0.32;5.59)
Obwalden	Local-weighted	5 (-2;11)	2.09% (-0.93;4.09)	6 (-3;13)	2.2% (-1.26;4.94)
Obwalden	Local-unweighted	6 (-3;11)	2.14% (-1.05;4.2)	6 (-4;13)	2.42% (-1.35;5.07)
Obwalden	Global-weighted	4 (-3;9)	1.67% (-0.98;3.59)	5 (-3;11)	2.06% (-1.06;4.16)
Obwalden	Global-unweighted	5 (-2;10)	1.94% (-0.62;3.9)	6 (-2;11)	2.27% (-0.86;4.33)
Schaffhausen	Weather station	4 (-9;14)	0.54% (-1.17;1.85)	5 (-12;18)	0.63% (-1.61;2.46)
Schaffhausen	Local-weighted	4 (-10;15)	0.53% (-1.33;1.99)	5 (-10;18)	0.73% (-1.41;2.44)
Schaffhausen	Local-unweighted	4 (-9;15)	0.58% (-1.18;2.05)	5 (-11;19)	0.72% (-1.42;2.55)
Schaffhausen	Global-weighted	4 (-9;15)	0.56% (-1.28;2.04)	7 (-11;22)	0.93% (-1.54;2.94)
Schaffhausen	Global-unweighted	3 (-10;14)	0.44% (-1.3;1.86)	6 (-12;21)	0.86% (-1.66;2.79)
Schwyz	Weather station	2 (-3;6)	0.24% (-0.35;0.64)	16 (-12;40)	1.64% (-1.27;4.07)
Schwyz	Local-weighted	2 (-6;7)	0.16% (-0.59;0.66)	13 (-13;37)	1.36% (-1.29;3.74)
Schwyz	Local-unweighted	1 (-5;6)	0.11% (-0.49;0.58)	10 (-19;34)	1.02% (-1.89;3.51)
Schwyz	Global-weighted	2 (-5;7)	0.25% (-0.46;0.74)	13 (-16;36)	1.28% (-1.66;3.72)
Schwyz	Global-unweighted	3 (-3;7)	0.27% (-0.34;0.75)	13 (-13;38)	1.28% (-1.36;3.84)
Solothurn	Weather station	2 (-5;8)	0.11% (-0.21;0.37)	17 (-28;54)	0.79% (-1.27;2.45)
Solothurn	Local-weighted	1 (-5;7)	0.06% (-0.2;0.3)	13 (-24;48)	0.59% (-1.08;2.16)

Solothurn	Local-unweighted	1 (-6;6)	0.06% (-0.25;0.29)	13 (-25;50)	0.6% (-1.11;2.24)
Solothurn	Global-weighted	0 (-6;6)	0.01% (-0.28;0.27)	6 (-38;43)	0.27% (-1.7;1.96)
Solothurn	Global-unweighted	0 (-6;6)	0.01% (-0.28;0.26)	3 (-40;43)	0.12% (-1.8;1.94)
St..Gallen	Weather station	15 (2;26)	0.39% (0.06;0.68)	36 (-15;86)	0.96% (-0.41;2.27)
St..Gallen	Local-weighted	15 (2;25)	0.39% (0.07;0.65)	36 (-15;78)	0.95% (-0.39;2.06)
St..Gallen	Local-unweighted	16 (3;27)	0.41% (0.08;0.7)	40 (-6;82)	1.05% (-0.17;2.16)
St..Gallen	Global-weighted	8 (-6;21)	0.21% (-0.17;0.55)	22 (-27;66)	0.57% (-0.71;1.75)
St..Gallen	Global-unweighted	7 (-9;19)	0.19% (-0.23;0.51)	21 (-33;69)	0.55% (-0.88;1.81)
Thurgau	Weather station	-1 (-11;9)	-0.03% (-0.61;0.5)	0 (-20;19)	0.02% (-1.06;1.02)
Thurgau	Local-weighted	-1 (-12;10)	-0.03% (-0.66;0.56)	1 (-16;14)	0.06% (-0.84;0.76)
Thurgau	Local-unweighted	0 (-12;10)	-0.02% (-0.65;0.54)	1 (-14;14)	0.06% (-0.75;0.76)
Thurgau	Global-weighted	4 (-5;13)	0.23% (-0.3;0.69)	7 (-18;28)	0.37% (-0.99;1.51)
Thurgau	Global-unweighted	4 (-6;13)	0.22% (-0.32;0.68)	6 (-21;30)	0.33% (-1.11;1.59)
Ticino	Weather station	14 (5;24)	0.51% (0.18;0.83)	18 (-2;36)	0.62% (-0.06;1.27)
Ticino	Local-weighted	15 (6;26)	0.54% (0.2;0.92)	19 (3;33)	0.66% (0.12;1.15)
Ticino	Local-unweighted	16 (3;29)	0.57% (0.11;1.01)	19 (7;29)	0.66% (0.25;1.03)
Ticino	Global-weighted	18 (5;29)	0.62% (0.19;1.01)	18 (3;33)	0.65% (0.11;1.15)
Ticino	Global-unweighted	19 (9;29)	0.69% (0.32;1.02)	23 (1;43)	0.8% (0.04;1.54)
Uri	Weather station	2 (-3;6)	0.55% (-0.86;1.84)	4 (-2;8)	1.26% (-0.54;2.57)
Uri	Local-weighted	3 (-2;8)	0.97% (-0.72;2.47)	3 (-1;7)	1.04% (-0.4;2.14)
Uri	Local-unweighted	3 (-3;7)	0.87% (-0.85;2.22)	4 (-1;7)	1.17% (-0.25;2.29)
Uri	Global-weighted	3 (-1;6)	0.82% (-0.33;1.81)	6 (-2;12)	1.91% (-0.54;3.82)
Uri	Global-unweighted	3 (-1;5)	0.82% (-0.45;1.73)	4 (-5;10)	1.29% (-1.74;3.35)
Valais	Weather station	23 (6;41)	0.99% (0.26;1.78)	24 (6;41)	1.05% (0.25;1.8)
Valais	Local-weighted	20 (1;39)	0.89% (0.04;1.69)	22 (0;38)	0.95% (0;1.67)
Valais	Local-unweighted	19 (-1;39)	0.84% (-0.06;1.71)	20 (-3;41)	0.88% (-0.13;1.8)
Valais	Global-weighted	19 (1;35)	0.82% (0.03;1.55)	21 (3;36)	0.91% (0.13;1.56)
Valais	Global-unweighted	18 (-4;37)	0.8% (-0.17;1.62)	20 (-6;45)	0.86% (-0.27;1.95)
Vaud	Weather station	19 (-21;54)	0.35% (-0.38;1)	25 (-51;98)	0.46% (-0.94;1.8)

Vaud	Local-weighted	19 (-22;55)	0.35% (-0.41;1.02)	31 (-50;106)	0.57% (-0.92;1.96)
Vaud	Local-unweighted	19 (-21;54)	0.36% (-0.39;0.99)	34 (-48;106)	0.64% (-0.89;1.97)
Vaud	Global-weighted	23 (-19;62)	0.42% (-0.35;1.14)	54 (-35;134)	1% (-0.65;2.48)
Vaud	Global-unweighted	23 (-16;58)	0.42% (-0.29;1.08)	47 (-32;133)	0.87% (-0.59;2.47)
Zug	Weather station	0 (-7;4)	-0.03% (-1.08;0.61)	3 (-22;22)	0.42% (-3.36;3.29)
Zug	Local-weighted	0 (-8;4)	-0.05% (-1.17;0.65)	4 (-20;22)	0.61% (-3.01;3.35)
Zug	Local-unweighted	0 (-7;4)	-0.01% (-0.99;0.64)	4 (-20;24)	0.63% (-3.04;3.65)
Zug	Global-weighted	3 (-2;6)	0.41% (-0.31;0.92)	10 (-13;27)	1.5% (-1.93;4)
Zug	Global-unweighted	3 (-2;7)	0.42% (-0.31;1)	12 (-12;31)	1.74% (-1.81;4.59)
Zürich	Weather station	71 (22;113)	0.67% (0.21;1.07)	74 (16;129)	0.7% (0.15;1.22)
Zürich	Local-weighted	70 (25;112)	0.66% (0.24;1.06)	78 (22;130)	0.74% (0.21;1.23)
Zürich	Local-unweighted	77 (29;123)	0.73% (0.27;1.17)	81 (25;135)	0.77% (0.23;1.28)
Zürich	Global-weighted	81 (32;126)	0.77% (0.3;1.19)	88 (24;144)	0.83% (0.22;1.36)
Zürich	Global-unweighted	79 (35;126)	0.75% (0.33;1.19)	88 (24;148)	0.83% (0.23;1.4)

Table S14. Summary statistics for the annual excess number of deaths, mortality fractions and associated 95% CI for cold (≤ 10th percentile and ≤ 25th percentile) in Switzerland

		≤ 10th percentile		≤ 25th percentile	
**Canton**	**GCD**	**AN**	**AF**	**AN**	**AF**
Aargau	Weather station	34 (-22;86)	0.81% (-0.52;2.05)	50 (-71;164)	1.2% (-1.7;3.93)
Aargau	Local-weighted	20 (-31;69)	0.48% (-0.75;1.66)	25 (-100;138)	0.59% (-2.4;3.3)
Aargau	Local-unweighted	23 (-30;65)	0.56% (-0.73;1.56)	22 (-95;128)	0.52% (-2.27;3.08)
Aargau	Global-weighted	31 (-14;72)	0.75% (-0.34;1.73)	41 (-75;143)	0.99% (-1.8;3.43)
Aargau	Global-unweighted	31 (-10;70)	0.74% (-0.24;1.68)	41 (-61;137)	0.99% (-1.47;3.29)
Appenzell.Ausserhoden	Weather station	-5 (-18;5)	-0.93% (-3.47;1.05)	-7 (-26;8)	-1.44% (-5.08;1.63)
Appenzell.Ausserhoden	Local-weighted	-5 (-16;5)	-1% (-3.12;1.03)	-7 (-26;10)	-1.4% (-5.13;1.86)
Appenzell.Ausserhoden	Local-unweighted	-5 (-17;6)	-0.88% (-3.28;1.07)	-7 (-26;10)	-1.3% (-5.12;1.9)
Appenzell.Ausserhoden	Global-weighted	1 (-12;11)	0.15% (-2.28;2.18)	2 (-18;20)	0.41% (-3.52;3.79)
Appenzell.Ausserhoden	Global-unweighted	1 (-10;11)	0.11% (-1.97;2.05)	1 (-18;18)	0.22% (-3.52;3.54)
Appenzell.Innerhoden	Weather station	4 (-5;9)	3.13% (-3.32;6.82)	12 (-8;22)	8.55% (-6.03;16.34)
Appenzell.Innerhoden	Local-weighted	4 (-4;9)	3.22% (-2.94;6.71)	12 (-7;23)	8.93% (-5.1;16.58)
Appenzell.Innerhoden	Local-unweighted	5 (-4;9)	3.36% (-2.56;6.91)	12 (-5;23)	8.98% (-3.7;16.51)
Appenzell.Innerhoden	Global-weighted	4 (-8;10)	2.91% (-5.73;7.28)	11 (-14;24)	8.09% (-9.92;17.8)
Appenzell.Innerhoden	Global-unweighted	4 (-8;10)	3.18% (-5.56;7.32)	11 (-13;24)	8.1% (-9.15;17.57)
Basel.Landschaft	Weather station	51 (18;78)	2.45% (0.84;3.74)	102 (26;166)	4.9% (1.26;7.96)
Basel.Landschaft	Local-weighted	49 (15;77)	2.34% (0.71;3.71)	99 (22;167)	4.73% (1.03;7.99)
Basel.Landschaft	Local-unweighted	47 (13;75)	2.23% (0.62;3.6)	94 (18;161)	4.48% (0.85;7.69)
Basel.Landschaft	Global-weighted	46 (11;77)	2.19% (0.53;3.68)	85 (-5;158)	4.08% (-0.24;7.55)
Basel.Landschaft	Global-unweighted	46 (12;73)	2.21% (0.57;3.51)	87 (4;159)	4.17% (0.18;7.6)
Basel.Stadt	Weather station	50 (15;79)	2.17% (0.66;3.41)	102 (28;171)	4.4% (1.2;7.37)
Basel.Stadt	Local-weighted	49 (14;79)	2.11% (0.62;3.38)	102 (24;172)	4.39% (1.02;7.38)
Basel.Stadt	Local-unweighted	47 (15;77)	2.03% (0.65;3.32)	99 (11;168)	4.24% (0.46;7.2)
Basel.Stadt	Global-weighted	57 (27;84)	2.46% (1.17;3.61)	117 (48;186)	5.03% (2.05;8.01)
Basel.Stadt	Global-unweighted	56 (25;83)	2.42% (1.07;3.56)	123 (48;185)	5.29% (2.07;7.94)

Bern	Weather station	167 (98;233)	1.81% (1.06;2.52)	347 (178;495)	3.76% (1.93;5.36)
Bern	Local-weighted	156 (90;216)	1.69% (0.98;2.34)	319 (160;464)	3.46% (1.73;5.03)
Bern	Local-unweighted	137 (77;196)	1.48% (0.83;2.13)	269 (118;403)	2.92% (1.27;4.37)
Bern	Global-weighted	151 (83;215)	1.64% (0.9;2.32)	319 (167;458)	3.46% (1.8;4.96)
Bern	Global-unweighted	149 (79;215)	1.61% (0.85;2.33)	313 (146;459)	3.39% (1.58;4.98)
Fribourg	Weather station	20 (-15;46)	1.03% (-0.77;2.44)	45 (-25;115)	2.38% (-1.32;6.04)
Fribourg	Local-weighted	20 (-13;46)	1.04% (-0.68;2.44)	46 (-26;113)	2.44% (-1.35;5.95)
Fribourg	Local-unweighted	21 (-12;48)	1.1% (-0.61;2.5)	51 (-19;113)	2.71% (-0.98;5.93)
Fribourg	Global-weighted	18 (-16;46)	0.94% (-0.83;2.42)	44 (-36;120)	2.34% (-1.91;6.31)
Fribourg	Global-unweighted	18 (-16;47)	0.94% (-0.83;2.47)	44 (-40;111)	2.3% (-2.09;5.83)
Genève	Weather station	43 (-4;84)	1.37% (-0.11;2.65)	95 (-18;190)	3.01% (-0.56;6.04)
Genève	Local-weighted	42 (-3;82)	1.33% (-0.08;2.61)	88 (-27;187)	2.8% (-0.86;5.92)
Genève	Local-unweighted	41 (-6;79)	1.3% (-0.21;2.49)	87 (-19;178)	2.75% (-0.61;5.66)
Genève	Global-weighted	36 (-9;74)	1.14% (-0.28;2.33)	65 (-40;157)	2.07% (-1.27;4.99)
Genève	Global-unweighted	34 (-13;74)	1.08% (-0.4;2.33)	67 (-37;158)	2.14% (-1.16;5.02)
Glarus	Weather station	10 (-3;19)	2.49% (-0.86;4.96)	25 (-6;46)	6.3% (-1.46;11.86)
Glarus	Local-weighted	5 (-7;16)	1.25% (-1.9;4.02)	15 (-11;37)	3.9% (-2.86;9.51)
Glarus	Local-unweighted	5 (-6;15)	1.38% (-1.6;3.74)	14 (-14;39)	3.65% (-3.59;9.91)
Glarus	Global-weighted	8 (-8;19)	1.92% (-2.03;4.82)	22 (-11;46)	5.62% (-2.73;11.86)
Glarus	Global-unweighted	8 (-7;20)	2.03% (-1.71;5.04)	23 (-12;49)	5.99% (-3.04;12.45)
Graübunden	Weather station	6 (-18;27)	0.34% (-1.12;1.66)	5 (-45;49)	0.31% (-2.73;2.99)
Graübunden	Local-weighted	8 (-13;26)	0.51% (-0.79;1.59)	14 (-27;53)	0.84% (-1.67;3.24)
Graübunden	Local-unweighted	10 (-9;28)	0.62% (-0.52;1.71)	19 (-22;57)	1.18% (-1.34;3.45)
Graübunden	Global-weighted	15 (-18;41)	0.9% (-1.13;2.53)	27 (-47;93)	1.66% (-2.86;5.67)
Graübunden	Global-unweighted	13 (-21;41)	0.81% (-1.27;2.51)	24 (-53;91)	1.49% (-3.25;5.54)
Jura	Weather station	6 (-11;20)	0.93% (-1.71;3.14)	15 (-26;47)	2.31% (-3.95;7.2)
Jura	Local-weighted	5 (-12;19)	0.74% (-1.9;2.97)	11 (-28;44)	1.71% (-4.26;6.69)
Jura	Local-unweighted	6 (-11;19)	0.85% (-1.71;2.96)	11 (-29;43)	1.74% (-4.39;6.52)
Jura	Global-weighted	2 (-13;13)	0.24% (-2.01;1.97)	2 (-26;24)	0.29% (-4;3.67)

Jura	Global-unweighted	1 (-15;13)	0.11% (-2.22;2.04)	2 (-26;28)	0.26% (-4.04;4.34)
Luzern	Weather station	53 (12;91)	1.89% (0.41;3.22)	103 (-3;192)	3.63% (-0.09;6.81)
Luzern	Local-weighted	52 (14;87)	1.85% (0.48;3.08)	107 (18;192)	3.81% (0.65;6.8)
Luzern	Local-unweighted	51 (10;87)	1.82% (0.34;3.1)	105 (9;189)	3.72% (0.31;6.7)
Luzern	Global-weighted	48 (7;84)	1.69% (0.23;2.97)	99 (1;184)	3.52% (0.05;6.51)
Luzern	Global-unweighted	48 (5;84)	1.72% (0.19;2.97)	100 (6;188)	3.55% (0.23;6.66)
Neuchâtel	Weather station	42 (9;65)	2.59% (0.57;4.05)	86 (7;147)	5.31% (0.46;9.08)
Neuchâtel	Local-weighted	40 (8;63)	2.46% (0.5;3.9)	83 (12;141)	5.15% (0.75;8.71)
Neuchâtel	Local-unweighted	38 (14;62)	2.36% (0.86;3.85)	79 (22;136)	4.92% (1.37;8.45)
Neuchâtel	Global-weighted	42 (16;65)	2.61% (0.98;4.03)	84 (20;137)	5.21% (1.27;8.51)
Neuchâtel	Global-unweighted	41 (16;63)	2.55% (1;3.92)	84 (13;141)	5.2% (0.78;8.75)
Nidwalden	Weather station	4 (-7;12)	1.48% (-2.62;4.42)	9 (-16;28)	3.5% (-5.81;10.49)
Nidwalden	Local-weighted	5 (-6;13)	1.9% (-2.17;4.78)	11 (-16;29)	4.24% (-5.98;11)
Nidwalden	Local-unweighted	4 (-7;11)	1.48% (-2.48;4.2)	9 (-16;27)	3.42% (-5.94;10.2)
Nidwalden	Global-weighted	7 (-5;15)	2.78% (-1.83;5.73)	17 (-12;34)	6.16% (-4.37;12.82)
Nidwalden	Global-unweighted	9 (-2;16)	3.31% (-0.91;6.1)	19 (-8;38)	7.1% (-2.85;14.14)
Obwalden	Weather station	8 (0;14)	3.19% (-0.14;5.48)	21 (2;36)	8.11% (0.61;13.76)
Obwalden	Local-weighted	7 (0;13)	2.8% (-0.14;4.93)	19 (0;32)	7.12% (-0.1;12.32)
Obwalden	Local-unweighted	7 (-2;13)	2.8% (-0.75;5.13)	19 (-2;33)	7.14% (-0.66;12.73)
Obwalden	Global-weighted	7 (-5;14)	2.55% (-1.85;5.44)	18 (-9;36)	6.9% (-3.5;13.64)
Obwalden	Global-unweighted	8 (-5;15)	3.02% (-1.87;5.74)	20 (-5;37)	7.83% (-2.05;14.15)
Schaffhausen	Weather station	7 (-15;25)	1.01% (-2.05;3.33)	11 (-41;53)	1.55% (-5.49;7.21)
Schaffhausen	Local-weighted	8 (-15;24)	1.02% (-2.02;3.28)	11 (-37;52)	1.5% (-5.06;7.03)
Schaffhausen	Local-unweighted	7 (-13;24)	0.93% (-1.81;3.3)	12 (-42;51)	1.56% (-5.63;6.89)
Schaffhausen	Global-weighted	3 (-15;18)	0.46% (-2.06;2.46)	1 (-38;35)	0.13% (-5.08;4.68)
Schaffhausen	Global-unweighted	4 (-16;19)	0.53% (-2.12;2.53)	0 (-44;35)	0.06% (-6.01;4.68)
Schwyz	Weather station	37 (15;55)	3.8% (1.51;5.61)	84 (33;121)	8.56% (3.39;12.39)
Schwyz	Local-weighted	35 (14;51)	3.54% (1.41;5.23)	80 (28;120)	8.17% (2.85;12.26)
Schwyz	Local-unweighted	31 (8;48)	3.14% (0.83;4.85)	74 (29;114)	7.53% (2.96;11.62)

Schwyz	Global-weighted	34 (11;51)	3.51% (1.16;5.23)	80 (23;119)	8.14% (2.33;12.1)
Schwyz	Global-unweighted	33 (10;51)	3.38% (1.06;5.17)	78 (27;119)	7.99% (2.75;12.18)
Solothurn	Weather station	41 (0;74)	1.84% (0.01;3.33)	84 (-15;170)	3.8% (-0.69;7.66)
Solothurn	Local-weighted	37 (-3;73)	1.65% (-0.15;3.31)	75 (-24;155)	3.39% (-1.1;6.99)
Solothurn	Local-unweighted	35 (-4;70)	1.59% (-0.17;3.14)	74 (-28;151)	3.33% (-1.24;6.8)
Solothurn	Global-weighted	34 (-8;68)	1.54% (-0.37;3.08)	62 (-32;149)	2.81% (-1.43;6.69)
Solothurn	Global-unweighted	32 (-10;67)	1.45% (-0.45;3)	64 (-40;154)	2.9% (-1.79;6.92)
St..Gallen	Weather station	74 (25;112)	1.95% (0.65;2.97)	154 (47;250)	4.06% (1.25;6.59)
St..Gallen	Local-weighted	74 (30;118)	1.96% (0.79;3.1)	157 (39;250)	4.15% (1.03;6.6)
St..Gallen	Local-unweighted	75 (30;112)	1.97% (0.78;2.96)	156 (58;244)	4.12% (1.54;6.43)
St..Gallen	Global-weighted	74 (23;115)	1.94% (0.61;3.04)	151 (31;257)	3.99% (0.82;6.78)
St..Gallen	Global-unweighted	72 (24;116)	1.89% (0.63;3.05)	148 (28;260)	3.9% (0.75;6.86)
Thurgau	Weather station	46 (14;73)	2.46% (0.76;3.91)	96 (21;157)	5.19% (1.14;8.43)
Thurgau	Local-weighted	45 (14;71)	2.42% (0.76;3.81)	98 (28;159)	5.28% (1.49;8.53)
Thurgau	Local-unweighted	45 (18;70)	2.44% (0.96;3.76)	100 (29;162)	5.36% (1.55;8.69)
Thurgau	Global-weighted	44 (13;72)	2.38% (0.72;3.87)	91 (8;158)	4.89% (0.42;8.48)
Thurgau	Global-unweighted	43 (12;71)	2.33% (0.66;3.81)	87 (2;154)	4.68% (0.13;8.3)
Ticino	Weather station	46 (-12;88)	1.62% (-0.43;3.1)	86 (-49;191)	3.03% (-1.74;6.76)
Ticino	Local-weighted	46 (-6;88)	1.61% (-0.21;3.13)	89 (-20;188)	3.14% (-0.7;6.63)
Ticino	Local-unweighted	46 (6;80)	1.64% (0.22;2.82)	87 (-10;176)	3.09% (-0.37;6.21)
Ticino	Global-weighted	46 (-2;92)	1.63% (-0.07;3.27)	82 (-41;193)	2.89% (-1.43;6.81)
Ticino	Global-unweighted	50 (5;94)	1.75% (0.17;3.31)	99 (-18;197)	3.49% (-0.65;6.97)
Uri	Weather station	4 (-13;14)	1.37% (-4.03;4.5)	16 (-18;37)	5.23% (-5.81;11.84)
Uri	Local-weighted	8 (-3;16)	2.43% (-1.1;4.98)	20 (-3;37)	6.53% (-1.06;11.92)
Uri	Local-unweighted	8 (-4;16)	2.59% (-1.17;5.07)	21 (0;38)	6.82% (-0.06;12.02)
Uri	Global-weighted	7 (-7;17)	2.37% (-2.39;5.4)	23 (-9;42)	7.26% (-2.93;13.52)
Uri	Global-unweighted	8 (-6;17)	2.42% (-1.9;5.54)	23 (-10;44)	7.22% (-3.23;13.96)
Valais	Weather station	19 (-21;58)	0.83% (-0.9;2.54)	52 (-49;130)	2.27% (-2.14;5.69)
Valais	Local-weighted	18 (-19;49)	0.8% (-0.84;2.13)	40 (-41;114)	1.76% (-1.79;5)

Valais	Local-unweighted	16 (-15;44)	0.69% (-0.68;1.94)	32 (-44;103)	1.38% (-1.91;4.51)
Valais	Global-weighted	31 (-3;63)	1.34% (-0.15;2.77)	61 (-29;142)	2.68% (-1.29;6.19)
Valais	Global-unweighted	25 (-12;59)	1.1% (-0.5;2.58)	53 (-40;131)	2.31% (-1.75;5.73)
Vaud	Weather station	38 (-5;76)	0.7% (-0.1;1.41)	67 (-15;144)	1.24% (-0.27;2.67)
Vaud	Local-weighted	37 (-2;73)	0.68% (-0.04;1.35)	62 (-12;129)	1.15% (-0.22;2.38)
Vaud	Local-unweighted	33 (-2;68)	0.61% (-0.04;1.25)	54 (-15;117)	1% (-0.28;2.17)
Vaud	Global-weighted	43 (3;77)	0.79% (0.05;1.42)	63 (-9;130)	1.16% (-0.17;2.41)
Vaud	Global-unweighted	39 (3;74)	0.73% (0.05;1.37)	61 (-16;128)	1.13% (-0.29;2.36)
Zug	Weather station	23 (2;36)	3.4% (0.35;5.47)	54 (5;86)	8.12% (0.72;12.94)
Zug	Local-weighted	21 (3;35)	3.09% (0.4;5.26)	51 (6;85)	7.61% (0.93;12.83)
Zug	Local-unweighted	20 (1;35)	3.07% (0.18;5.25)	49 (6;80)	7.38% (0.84;12.07)
Zug	Global-weighted	19 (-2;33)	2.83% (-0.29;4.97)	46 (1;81)	6.98% (0.19;12.23)
Zug	Global-unweighted	19 (-2;34)	2.85% (-0.36;5.13)	48 (-3;80)	7.2% (-0.51;11.95)
Zürich	Weather station	155 (89;220)	1.47% (0.84;2.08)	305 (139;458)	2.89% (1.31;4.33)
Zürich	Local-weighted	156 (90;223)	1.48% (0.85;2.11)	311 (152;465)	2.94% (1.44;4.39)
Zürich	Local-unweighted	150 (81;212)	1.41% (0.77;2)	298 (136;445)	2.81% (1.29;4.21)
Zürich	Global-weighted	141 (69;209)	1.34% (0.65;1.97)	276 (108;427)	2.61% (1.03;4.03)
Zürich	Global-unweighted	144 (72;208)	1.36% (0.68;1.97)	283 (112;437)	2.67% (1.06;4.13)

Table S15. Summary statistics for the annual excess number of deaths, mortality fractions and associated 95% CI for heat (≥ 90th percentile and ≥75th percentile) for England and Wales

		≥ 90th percentile		≥ 75th percentile	
Region	GCD	AN	AF	AN	AF
East England	Weather station	170 (112;232)	0.32% (0.21;0.43)	388 (164;622)	0.72% (0.31;1.16)
East England	Local-weighted	176 (110;240)	0.33% (0.21;0.45)	386 (177;594)	0.72% (0.33;1.11)
East England	Local-unweighted	175 (101;253)	0.33% (0.19;0.47)	279 (117;448)	0.52% (0.22;0.84)
East England	Global-weighted	222 (158;284)	0.41% (0.4;0.53)	395 (204;581)	0.74% (0.77;1.09)
East England	Global-unweighted	216 (148;286)	0.4% (0.28;0.53)	412 (202;620)	0.77% (0.38;1.16)
Yorkshire and the Humber	Weather station	141 (19;259)	0.26% (0.04;0.48)	181 (74;282)	0.34% (0.14;0.53)
Yorkshire and the Humber	Local-weighted	136 (18;254)	0.25% (0.03;0.48)	183 (84;284)	0.34% (0.16;0.53)
Yorkshire and the Humber	Local-unweighted	125 (8;235)	0.23% (0.01;0.44)	171 (76;248)	0.32% (0.14;0.46)
Yorkshire and the Humber	Global-weighted	162 (37;299)	0.3% (0.29;0.56)	201 (72;320)	0.38% (0.37;0.6)
Yorkshire and the Humber	Global-unweighted	156 (38;274)	0.29% (0.07;0.51)	195 (80;308)	0.37% (0.15;0.58)
Southeast England	Weather station	252 (163;336)	0.31% (0.2;0.42)	387 (189;577)	0.48% (0.23;0.71)
Southeast England	Local-weighted	251 (155;340)	0.31% (0.19;0.42)	399 (219;566)	0.49% (0.27;0.7)
Southeast England	Local-unweighted	255 (95;415)	0.32% (0.12;0.51)	297 (145;449)	0.37% (0.18;0.55)
Southeast England	Global-weighted	307 (172;449)	0.38% (0.38;0.56)	367 (262;462)	0.45% (0.45;0.57)
Southeast England	Global-unweighted	304 (174;438)	0.38% (0.22;0.54)	361 (259;455)	0.45% (0.32;0.56)
Eastmidlands	Weather station	105 (35;175)	0.24% (0.08;0.4)	199 (-6;393)	0.46% (-0.01;0.91)
Eastmidlands	Local-weighted	101 (29;173)	0.23% (0.07;0.4)	196 (11;362)	0.45% (0.02;0.84)
Eastmidlands	Local-unweighted	85 (8;171)	0.2% (0.02;0.39)	122 (10;222)	0.28% (0.02;0.51)
Eastmidlands	Global-weighted	123 (47;194)	0.28% (0.28;0.45)	175 (32;309)	0.4% (0.43;0.71)
Eastmidlands	Global-unweighted	122 (48;196)	0.28% (0.11;0.45)	187 (54;315)	0.43% (0.13;0.73)
Greater London	Weather station	338 (280;397)	0.56% (0.46;0.66)	760 (504;1000)	1.26% (0.83;1.66)
Greater London	Local-weighted	309 (250;363)	0.51% (0.41;0.6)	716 (505;930)	1.19% (0.84;1.54)
Greater London	Local-unweighted	231 (149;309)	0.38% (0.25;0.51)	394 (206;580)	0.66% (0.34;0.96)
Greater London	Global-weighted	344 (278;407)	0.57% (0.57;0.67)	769 (523;993)	1.27% (1.26;1.64)
Greater London	Global-unweighted	342 (277;402)	0.57% (0.46;0.67)	758 (491;1033)	1.26% (0.81;1.71)
Southwest England	Weather station	220 (73;352)	0.4% (0.13;0.64)	253 (114;401)	0.46% (0.21;0.73)
Southwest England	Local-weighted	210 (55;358)	0.38% (0.1;0.65)	242 (68;414)	0.44% (0.12;0.75)

Southwest England	Local-unweighted	205 (68;361)	0.37% (0.12;0.66)	242 (78;408)	0.44% (0.14;0.74)
Southwest England	Global-weighted	210 (85;340)	0.38% (0.38;0.62)	247 (108;377)	0.45% (0.44;0.68)
Southwest England	Global-unweighted	209 (66;363)	0.38% (0.12;0.66)	241 (93;412)	0.44% (0.17;0.75)
Westmidlands	Weather station	102 (27;176)	0.18% (0.05;0.32)	254 (35;462)	0.46% (0.06;0.84)
Westmidlands	Local-weighted	116 (36;189)	0.21% (0.07;0.34)	281 (6;529)	0.51% (0.01;0.96)
Westmidlands	Local-unweighted	67 (-23;142)	0.12% (-0.04;0.26)	160 (-147;441)	0.29% (-0.27;0.8)
Westmidlands	Global-weighted	109 (26;188)	0.2% (0.18;0.34)	191 (52;334)	0.35% (0.3;0.61)
Westmidlands	Global-unweighted	102 (-2;197)	0.18% (0;0.36)	166 (73;260)	0.3% (0.13;0.47)
Northwest England	Weather station	243 (92;388)	0.32% (0.12;0.51)	305 (180;427)	0.4% (0.24;0.56)
Northwest England	Local-weighted	267 (124;401)	0.35% (0.16;0.52)	315 (197;431)	0.41% (0.26;0.56)
Northwest England	Local-unweighted	148 (24;269)	0.19% (0.03;0.35)	197 (102;294)	0.26% (0.13;0.38)
Northwest England	Global-weighted	279 (103;446)	0.36% (0.31;0.58)	331 (167;484)	0.43% (0.38;0.63)
Northwest England	Global-unweighted	240 (76;413)	0.31% (0.1;0.54)	292 (121;457)	0.38% (0.16;0.6)
Northeast England	Weather station	109 (10;202)	0.37% (0.03;0.69)	132 (44;221)	0.45% (0.15;0.76)
Northeast England	Local-weighted	98 (6;193)	0.34% (0.02;0.66)	119 (35;199)	0.41% (0.12;0.68)
Northeast England	Local-unweighted	92 (19;165)	0.32% (0.06;0.57)	116 (52;172)	0.4% (0.18;0.59)
Northeast England	Global-weighted	111 (35;175)	0.38% (0.34;0.6)	127 (59;186)	0.44% (0.41;0.64)
Northeast England	Global-unweighted	100 (24;175)	0.34% (0.08;0.6)	119 (56;181)	0.41% (0.19;0.62)
Wales	Weather station	95 (-5;191)	0.28% (-0.01;0.57)	117 (32;197)	0.35% (0.1;0.58)
Wales	Local-weighted	93 (0;189)	0.28% (0;0.56)	115 (32;191)	0.34% (0.1;0.57)
Wales	Local-unweighted	74 (-21;170)	0.22% (-0.06;0.5)	89 (19;168)	0.26% (0.06;0.5)
Wales	Global-weighted	86 (-19;174)	0.26% (0.23;0.52)	111 (31;186)	0.33% (0.34;0.55)
Wales	Global-unweighted	79 (5;145)	0.23% (0.01;0.43)	114 (30;182)	0.34% (0.09;0.54)

Table S16. Summary statistics for the annual excess number of deaths, excess mortality fractions and associated 95% CI for cold (≤10th percentile and ≤25th percentile) for England and Wales

		≤ 10th percentile		≤ 25th percentile	
Region	GCD	AN	AF	AN	AF
East England	Weather station	1209 (932;1451)	2.26% (0.2 ; 0.43)	2348 (1701;2967)	4.39% (3.18;5.54)
East England	Local-weighted	1215 (957;1466)	2.27% (0.2 ; 0.44)	2354 (1697;2912)	4.4% (3.17;5.44)
East England	Local-unweighted	1115 (880;1361)	2.08% (0.19 ; 0.47)	2065 (1425;2704)	3.86% (2.66;5.05)
East England	Global-weighted	1138 (878;1364)	2.13% (0.28 ; 0.53)	2163 (1516;2743)	4.04% (4.06;5.12)
East England	Global-unweighted	1149 (878;1396)	2.15% (0.27 ; 0.53)	2172 (1486;2785)	4.06% (2.78;5.2)
Yorkshire and the Humber	Weather station	898 (623;1151)	1.68% (0.05 ; 0.47)	1855 (1233;2424)	3.47% (2.31;4.54)
Yorkshire and the Humber	Local-weighted	891 (626;1130)	1.67% (0.03 ; 0.5)	1863 (1231;2481)	3.49% (2.31;4.65)
Yorkshire and the Humber	Local-unweighted	828 (587;1078)	1.55% (0.03 ; 0.45)	1682 (1092;2287)	3.15% (2.05;4.28)
Yorkshire and the Humber	Global-weighted	821 (549;1050)	1.54% (0.03 ; 0.56)	1712 (1063;2283)	3.21% (3.24;4.28)
Yorkshire and the Humber	Global-unweighted	813 (557;1049)	1.52% (0.05 ; 0.55)	1729 (1075;2313)	3.24% (2.01;4.33)
Southeast England	Weather station	1911 (1596;2219)	2.36% (0.21 ; 0.42)	3651 (2919;4427)	4.51% (3.61;5.47)
Southeast England	Local-weighted	1961 (1658;2246)	2.42% (0.2 ; 0.42)	3711 (2987;4502)	4.59% (3.69;5.56)
Southeast England	Local-unweighted	1879 (1570;2162)	2.32% (0.1 ; 0.55)	3528 (2798;4200)	4.36% (3.46;5.19)
Southeast England	Global-weighted	1829 (1520;2133)	2.26% (0.22 ; 0.54)	3439 (2639;4172)	4.25% (4.28;5.16)
Southeast England	Global-unweighted	1844 (1532;2141)	2.28% (0.21 ; 0.55)	3461 (2673;4150)	4.28% (3.3;5.13)
Eastmidlands	Weather station	877 (627;1130)	2.02% (0.09 ; 0.4)	1752 (1166;2277)	4.04% (2.69;5.26)
Eastmidlands	Local-weighted	889 (637;1113)	2.05% (0.08 ; 0.38)	1731 (1165;2261)	4% (2.69;5.22)
Eastmidlands	Local-unweighted	779 (561;991)	1.8% (0.01 ; 0.39)	1464 (893;1965)	3.38% (2.06;4.54)
Eastmidlands	Global-weighted	759 (532;984)	1.75% (0.12 ; 0.44)	1485 (919;1995)	3.43% (3.51;4.61)
Eastmidlands	Global-unweighted	782 (553;989)	1.81% (0.11 ; 0.45)	1520 (1010;2067)	3.51% (2.33;4.77)
Greater London	Weather station	1603 (1322;1851)	2.65% (0.44 ; 0.64)	3200 (2566;3816)	5.3% (4.25;6.32)
Greater London	Local-weighted	1600 (1359;1863)	2.65% (0.4 ; 0.6)	3199 (2544;3836)	5.3% (4.21;6.35)
Greater London	Local-unweighted	1454 (1175;1688)	2.4% (0.26 ; 0.5)	2799 (2161;3447)	4.64% (3.58;5.71)
Greater London	Global-weighted	1531 (1244;1781)	2.54% (0.45 ; 0.67)	3033 (2328;3672)	5.02% (5.05;6.08)
Greater London	Global-unweighted	1531 (1245;1771)	2.54% (0.45 ; 0.65)	3048 (2307;3651)	5.05% (3.82;6.05)

Southwest England	Weather station	1417 (1191;1632)	2.57% (0.12 ; 0.68)	2724 (2142;3322)	4.95% (3.89;6.03)
Southwest England	Local-weighted	1438 (1205;1660)	2.61% (0.07 ; 0.7)	2773 (2182;3325)	5.03% (3.96;6.04)
Southwest England	Local-unweighted	1348 (1132;1585)	2.45% (0.09 ; 0.69)	2525 (1955;3092)	4.58% (3.55;5.61)
Southwest England	Global-weighted	1327 (1106;1545)	2.41% (0.15 ; 0.67)	2486 (1903;3063)	4.51% (4.52;5.56)
Southwest England	Global-unweighted	1328 (1109;1550)	2.41% (0.13 ; 0.74)	2490 (1946;3088)	4.52% (3.53;5.6)
Westmidlands	Weather station	1100 (827;1343)	1.99% (0.03 ; 0.32)	2111 (1435;2686)	3.83% (2.6;4.87)
Westmidlands	Local-weighted	1100 (833;1380)	2% (0.06 ; 0.33)	2128 (1454;2765)	3.86% (2.64;5.02)
Westmidlands	Local-unweighted	926 (652;1196)	1.68% (-0.04 ; 0.26)	1749 (1108;2385)	3.17% (2.01;4.33)
Westmidlands	Global-weighted	891 (629;1145)	1.62% (0.04 ; 0.36)	1660 (1058;2280)	3.01% (2.98;4.14)
Westmidlands	Global-unweighted	898 (642;1128)	1.63% (0.01 ; 0.36)	1642 (986;2228)	2.98% (1.79;4.04)
Northwest England	Weather station	1425 (1138;1692)	1.87% (0.13 ; 0.53)	2997 (2306;3652)	3.92% (3.02;4.78)
Northwest England	Local-weighted	1449 (1138;1725)	1.9% (0.15 ; 0.54)	3021 (2333;3675)	3.95% (3.05;4.81)
Northwest England	Local-unweighted	1377 (1070;1697)	1.8% (0.02 ; 0.35)	2776 (1969;3503)	3.63% (2.58;4.58)
Northwest England	Global-weighted	1279 (957;1584)	1.67% (0.11 ; 0.59)	2697 (2003;3418)	3.53% (3.48;4.47)
Northwest England	Global-unweighted	1274 (981;1560)	1.67% (0.08 ; 0.56)	2658 (1916;3334)	3.48% (2.51;4.36)
Northeast England	Weather station	435 (256;601)	1.49% (0.01 ; 0.72)	856 (424;1291)	2.94% (1.46;4.43)
Northeast England	Local-weighted	446 (267;610)	1.53% (-0.03 ; 0.65)	892 (390;1302)	3.06% (1.34;4.47)
Northeast England	Local-unweighted	464 (283;631)	1.59% (0.06 ; 0.54)	902 (453;1311)	3.1% (1.56;4.5)
Northeast England	Global-weighted	425 (233;590)	1.46% (0.12 ; 0.62)	852 (405;1254)	2.92% (2.89;4.31)
Northeast England	Global-unweighted	415 (236;583)	1.42% (0.06 ; 0.64)	843 (424;1264)	2.89% (1.46;4.34)
Wales	Weather station	802 (631;975)	2.38% (-0.04 ; 0.59)	1589 (1142;2000)	4.71% (3.39;5.93)
Wales	Local-weighted	789 (601;960)	2.34% (-0.02 ; 0.59)	1545 (1095;1972)	4.58% (3.25;5.85)
Wales	Local-unweighted	800 (613;970)	2.37% (-0.07 ; 0.5)	1565 (1107;1976)	4.64% (3.28;5.86)
Wales	Global-weighted	760 (573;922)	2.25% (-0.04 ; 0.55)	1471 (982;1875)	4.36% (4.11;5.56)
Wales	Global-unweighted	732 (564;897)	2.17% (0.03 ; 0.45)	1384 (962;1804)	4.11% (2.85;5.35)
